# Supplementary material for: Do general practitioners working in or alongside the emergency department improve clinical outcomes or experience? A mixed-methods study
Source: BMJ Open. 2022 Sep 20;12(9):e063495. doi: 10.1136/bmjopen-2022-063495 (PMC9490584; doi:10.1136/bmjopen-2022-063495)
Supplement: Supplementary data [file bmjopen-2022-063495supp001.pdf]

GP ED

UWE Bristol University of the West of England

Setting: Established Case Sites

Participants: Staff in ED/GPED/KI

What is your current role in the GPED?

What model of working with GPs/primary care operates in your ED currently?

Were you involved (and in what way) in the design or initial implementation of GPED?

- only if indicate were involved, ask planning/implementation questions

Planning/implementation stage:

What can you tell us about the initial process of design and implementation of this service

- Key staff involved
- Structural/organisational changes
- Decision making/service design
- Consultation with staff/patients/external bodies

What was expected to be achieved by the change?

What were the key barriers/facilitators?

What were the key issues for staff before the introduction?

What was the attitude/approach to change from management?

Impact:

How do you think the GPED model is working?

- Process of selecting patients to be seen by the GP/streaming/getting the 'right' patients
- Key advantages/disadvantages
- Any safety issues

How has it impacted on overall workings of the ED?

- Has there been any impact on performance (e.g. 4 hours, hospital admission rate)
- Resources

Do you think any improvements could be made to the GPED model (aware of different service configurations in other places)?

GPED Topic Guide Established Sites/Staff in ED/GPED/KI (v1.0) 13-07-2017

What feedback have you had from patients about the GPED model (are they satisfied etc)?

Do you think the availability of this GPED model is likely to change the way the public decide how, where and when to seek care?

For emergency care staff:

How has GPED impacted on your own everyday working?

- Clinically (type of patients/presenting conditions)
- Working relationships with other staff (e.g. the staff who select patients to be seen by GP, the GP staff)
- Service provided to patients
- Administratively/organizationally
- Any surprises

For general practice staff in GPED:

How is care organised within GP component of GPED?

How does practice within GPED compare to other services (GP practice, walk-in centres):

- Clinically (types of patients/presenting conditions)
- Patient 'outcomes' (e.g. referrals, requests for testing, transfer back to ED)
- Interaction with other professional groups within GP component/ED staff
- Workload
- Any surprises

Discussion around who is employer, professional indemnity, clinical supervision/support around clinical decision making in role as GP in ED

Do you feel you act differently as a practitioner following time in ED (probe – both back in primary care and over time within ED)

Satisfaction with role of GP in ED

- Met with expectations
- Plan to continue in role
- Career plans

How do you think patients have responded to the service?

Scantlebury A, *et al. BMJ Open* 2022; 12:e063495. doi: 10.1136/bmjopen-2022-063495

GPED

- Why they came to AE rather than GP practice
- Satisfaction with GPED

Any other comments to add about GPED
