# Supplementary material for: Do general practitioners working in or alongside the emergency department improve clinical outcomes or experience? A mixed-methods study
Source: BMJ Open. 2022 Sep 20;12(9):e063495. doi: 10.1136/bmjopen-2022-063495 (PMC9490584; doi:10.1136/bmjopen-2022-063495)
Supplement: Supplementary data [file bmjopen-2022-063495supp002.pdf]

Setting: Existing Case Sites

Participants: Patients

What brought you to the ED on this occasion?

Tell us about what happened after you arrived?

- Who did you see first/what happened next
- Description of being selected to be seen by the GP

Did you know it was possible to be sent to a GP after coming to ED?

- Was this communicated to you
- Did you understand the process/reason you were selected for the GP
- How did you feel about being seen by a GP
- Have you any previous experience of this service (give example)

Explore reason behind attendance at ED for this consultation – why did they use ED over other potential services (walk-in centres, GP surgery)

- Knowledge of different ways to access health services and what they consider the 'appropriate' ways to use them

Would their experiences on this visit change their consultation choice in the future?

Explore awareness of increased demand on EDs/government funding made available to increase GPs in EDs

- Do they think GPs in ED good idea in principle
- What impact do they think it might have on reducing pressure on EDs
- Do they think it will change what patients do

How does practice within GPED compare to other GP services?

How satisfied are they with the visit?

- How long did you have to wait
- How satisfied are you with the outcome
- Can you think of any ways you could improve the service?
- Opportunity to provide feedback

Any other comments to add about GPED.

GPED Topic Guide Existing Sites/Patients (v.10) 13-07-2017
