# Supplementary material for: Do general practitioners working in or alongside the emergency department improve clinical outcomes or experience? A mixed-methods study
Source: BMJ Open. 2022 Sep 20;12(9):e063495. doi: 10.1136/bmjopen-2022-063495 (PMC9490584; doi:10.1136/bmjopen-2022-063495)
Supplement: Supplementary data [file bmjopen-2022-063495supp003.pdf]

GPED –Coding Framework

NATIONAL CONTEXT	National - policy, pressures
LOCAL CONTEXT	Local – service landscape and population/specific local needs/considerations
TRUST ED & UC CULTURE	RESPOND OR RESIST whether staff are actively redirecting patients away from the ED to resist the flow or providing a service in response and recognition that patients have attended with health concerns. Where staff feel they must see patients and responsibility rests with them to provide health care. UCC/GP in ED vs primary care (differences to traditional primary care role.
PEN PORTRAIT DATA	Explanation of current system, patient journey through the ED, Layout, History of GPED, future plans,
PATIENTS REASONS FOR ATTENDING ED	Patient and staff explanations of why patients attend ED/Previous use of services e.g. have they seen/contacted service before ED
SERVICE LITERACY	Any discussions around appropriate/inappropriate attendances, perceived impact of service literacy and actual patient service literacy on use of GPED/ED
IMPLEMENTATION	Perceived Challenges and Facilitators to Implementation
PERCEIVED IMPACT	Perceived impact of GPED on patient safety, workforce and skills mix, staff interactions, performance/targets, views of GPED
EXPECTATIONS OF GPED (T1)	'hypothesis' from stakeholders at all levels regarding their expectations of what would be the outcome of introduction to GPED. From T1 data, only prospective?
OTHER/MISCELLANEOUS INSIGHTS	Potential emerging insights which are outside the current framework but may be significant/to be reviewed with the WPC team regular meetings.
