# Supplementary material for: Do general practitioners working in or alongside the emergency department improve clinical outcomes or experience? A mixed-methods study
Source: BMJ Open. 2022 Sep 20;12(9):e063495. doi: 10.1136/bmjopen-2022-063495 (PMC9490584; doi:10.1136/bmjopen-2022-063495)
Supplement: Author's manuscript [file bmjopen-2022-063495.draft_revisions.pdf]

BMJ Open is committed to open peer review. As part of this commitment we make the peer review history of every article we publish publicly available.

When an article is published we post the peer reviewers' comments and the authors' responses online. We also post the versions of the paper that were used during peer review. These are the versions that the peer review comments apply to.

The versions of the paper that follow are the versions that were submitted during the peer review process. They are not the versions of record or the final published versions. They should not be cited or distributed as the published version of this manuscript.

BMJ Open is an open access journal and the full, final, typeset and author-corrected version of record of the manuscript is available on our site with no access controls, subscription charges or pay-per-view fees (http://bmjopen.bmj.com).

If you have any questions on BMJ Open's open peer review process please email info.bmjopen@bmj.com

BMJ Open

# Do General Practitioners Working in or Alongside the Emergency Department Improve Clinical Outcomes or Experience? A mixed methods study

| | |
|---|---|
| Journal: | *BMJ Open* |
| Manuscript ID | bmjopen-2022-063495 |
| Article Type: | Original research |
| Date Submitted by the Author: | 05-Apr-2022 |
| Complete List of Authors: | Scantlebury, Arabella; University of York Department of Health Sciences, York Trials Unit<br>Adamson, Joy; University of York, Department of Health Sciences<br>Salisbury, Chris; University of Bristol, Centre for Academic Primary Care, School of Social and Community Medicine<br>Brant, Heather; University of the West of England Faculty of Health and Applied Sciences<br>Anderson, Helen; University of York Department of Health Sciences<br>Baxter, Helen; University of Bristol, School of Social and Community Medicine<br>Bloor, Karen; University of York, Department of Health Sciences<br>Cowlishaw, Sean; The University of Melbourne, Department of Psychiatry ; University of Bristol,<br>Doran, Tim; University of York<br>Gaughan, James; University of York Department of Health Sciences<br>Gibson, Andy; University of the West of England Faculty of Health and Applied Sciences<br>Gutacker, Nils; University of York, Centre for Health Economics<br>Leggett, Heather; University of York Department of Health Sciences<br>Purdy, Sarah; University of Bristol School of Social and Community Medicine<br>Voss, Sarah; University of the West of England Faculty of Health and Applied Sciences<br>Benger, Jonathan; University of the West of England Faculty of Health and Applied Sciences |
| Keywords: | Health policy < HEALTH SERVICES ADMINISTRATION & MANAGEMENT, ACCIDENT & EMERGENCY MEDICINE, QUALITATIVE RESEARCH, PRIMARY CARE |
| | |

SCHOLARONE™
Manuscripts

*I, the Submitting Author has the right to grant and does grant on behalf of all authors of the Work (as defined in the below author licence), an exclusive licence and/or a non-exclusive licence for contributions from authors who are: i) UK Crown employees; ii) where BMJ has agreed a CC-BY licence shall apply, and/or iii) in accordance with the terms applicable for US Federal Government officers or employees acting as part of their official duties; on a worldwide, perpetual, irrevocable, royalty-free basis to BMJ Publishing Group Ltd ("BMJ") its licensees and where the relevant Journal is co-owned by BMJ to the co-owners of the Journal, to publish the Work in this journal and any other BMJ products and to exploit all rights, as set out in our licence.*

*The Submitting Author accepts and understands that any supply made under these terms is made by BMJ to the Submitting Author unless you are acting as an employee on behalf of your employer or a postgraduate student of an affiliated institution which is paying any applicable article publishing charge ("APC") for Open Access articles. Where the Submitting Author wishes to make the Work available on an Open Access basis (and intends to pay the relevant APC), the terms of reuse of such Open Access shall be governed by a Creative Commons licence – details of these licences and which Creative Commons licence will apply to this Work are set out in our licence referred to above.*

*Other than as permitted in any relevant BMJ Author's Self Archiving Policies, I confirm this Work has not been accepted for publication elsewhere, is not being considered for publication elsewhere and does not duplicate material already published. I confirm all authors consent to publication of this Work and authorise the granting of this licence.*

For peer review only

# Do General Practitioners Working in or Alongside the Emergency Department Improve Clinical Outcomes or Experience? A mixed methods study

**Authors names:** Arabella Scantlebury (0000-0003-3518-2740), Joy Adamson (0000-0002-9860-0850), Chris Salisbury (0000-0002-4378-3960), Heather Brant (0000-0001-9608-7451), Helen Anderson (0000-0002-6945-0590), Helen Baxter (0000-0002-3320-2915), Karen Bloor (0000-0003-4852-9854), Sean Cowlishaw (0000-0002-8523-3713), Tim Doran (0000-0001-7857-3704), James Gaughan (0000-0002-8409-140X), Andrew Gibson (0000-0002-4641-2583), Nils Gutacker (0000-0002-2833-0621), Heather Leggett (0000-0001-8708-9842), Sarah Purdy (0000-0002-3445-986X), Sarah Voss (0000-0001-5044-5145), Jonathan Benger (0000-0001-6131-0916).

York Trials Unit, Department of Health Sciences, ARRC Building, University of York, York, YO10 5DD, UK, Assistant Professor, Arabella Scantlebury

York Trials Unit, Department of Health Sciences, ARRC Building, University of York, York, YO10 5DD, UK, Professor, Joy Adamson

School of Social and Community Medicine, University of Bristol, Bristol, BS8 2PS, UK, Professor, Chris Salisbury

Faculty of Health and Applied Sciences, University of the West of England, Bristol, BS16 1QY UK, Senior Research Associate, Heather Brant

York Trials Unit, Department of Health Sciences, ARRC Building, University of York, York, YO10 5DD, UK, Research Fellow, Helen Anderson

School of Social and Community Medicine, University of Bristol, Bristol, BS8 2PS, UK, Research Fellow, Helen Baxter

Department of Health Sciences, ARRC Building, University of York, York, YO10 5DD, UK, Professor, Karen Bloor

Department of Psychiatry, University of Melbourne, Melbourne, VIC 3053, Australia, Senior Research Fellow, Sean Cowlishaw

Department of Health Sciences, ARRC Building, University of York, York, YO10 5DD, UK, Professor, Tim Doran

Department of Health Sciences, ARRC Building, University of York, York, YO10 5DD, UK, Research Fellow, James Gaughan

For peer review only

Faculty of Health and Applied Sciences, University of the West of England, Bristol, BS16 1QY, Associate Professor, Andrew Gibson

Department of Health Sciences, ARRC Building, University of York, York, YO10 5DD, UK, Senior Research Fellow, Nils Gutacker

York Trials Unit, Department of Health Sciences, ARRC Building, University of York, York, YO10 5DD, UK, Research Fellow, Heather Leggett

School of Social and Community Medicine, University of Bristol, Bristol, BS8 2PS, UK, Professor, Sarah Purdy

Faculty of Health and Applied Sciences, University of the West of England, Bristol, BS16 1QY, Professor, Sarah Voss

Faculty of Health and Applied Sciences, UWE Bristol, Glenside Campus, Blackberry Hill, Bristol, BS16 1DD, Professor, Jonathan Benger

**Correspondence to:** Jonathan Benger, Faculty of Health and Applied Sciences, University of the West of England, Bristol, BS16 1QY. Jonathan.Benger@uwe.ac.uk

For peer review only

For peer review only



For peer review only - http://bmjopen.bmj.com/site/about/guidelines.xhtml

# Abstract

**Objectives:** To examine the effect of General Practitioners (GPs) working in or alongside the Emergency Department (GPED) on patient outcomes and experience, and the associated workforce.

**Design:** Mixed-methods study: interviews with service leaders and NHS managers; in-depth case studies (n=10) and retrospective observational analysis of routinely collected national data. We used Normalisation Process Theory to map our findings to the theory's four main constructs of coherence, cognitive participation, collective action and reflexive monitoring.

**Setting and participants:** Data was collected from 64 Emergency Departments (ED) in England. Case site data included: non-participant observation of 142 clinical encounters; 413 semi-structured interviews with policy makers, service leaders, clinical staff, patients and carers. Retrospective observational analysis used routinely collected Hospital Episode Statistics alongside information on GPED service hours from 40 hospitals for which complete data were available.

**Results:** There was disagreement at individual, stakeholder and organisational levels regarding the purpose and potential impact of GPED (coherence). Participants criticised policy development and implementation, and staff engagement was hindered by tensions between ED and GP staff (cognitive participation). Patient "streaming" processes, staffing and resource constraints influenced whether GPED became embedded in routine practice. Concerns that GPED may increase ED attendance influenced staff views. Our quantitative analysis showed no detectable impact on attendance (collective action). Stakeholders disagreed whether GPED was successful, due to variations in GPED model, site-specific patient mix and governance arrangements. Following statistical adjustment for multiple testing, we found no impact on: ED re-attendances within seven days, patients discharged within four hours of arrival, patients leaving the ED without being seen; inpatient admissions; non-urgent ED attendances and 30-day mortality (reflexive monitoring).

**Conclusions:** We found a high degree of variability between hospital sites, but no overall evidence that GPED increases the efficient operation of EDs or improves clinical outcomes, patient or staff experience.


**Trial registration:** ISCRTN5178022



For peer review only - http://bmjopen.bmj.com/site/about/guidelines.xhtml

## Strengths and limitations of this study

- National evaluation of the impact of general practitioners working in or alongside emergency departments in England.
- Mixed methods approach using a large qualitative data set (413 interviews, 142 non-participant observation) and routine national data sets involving multiple stakeholders across 64 emergency departments gave us a service wide and detailed understanding of the impact of GPED.
- Our data apply to England only and so may not be generalizable to other countries and healthcare settings.
- Our quantitative analysis was limited to routinely available data and so our analysis was dependent on key performance indicators and what is routinely collected and reported.

## Introduction

There were almost 24 million attendances at hospital emergency departments (EDs) in England in 2017-18, an increase of 22% since 2007/8.[1] This continues a long-term trend of increasing demand for urgent care at EDs that has also been observed in many other countries.[2] Workload pressures within these departments can lead to adverse effects on the quality of patient care, patient safety, clinical outcomes, patient satisfaction and staff job satisfaction.[3] One important measure of the performance of emergency departments in England is the target that 95% of patients should be admitted, transferred or discharged within four hours of arrival. This target has not been met nationally since 2015, with performance declining every year.[1]

About a fifth of patients attending emergency departments could be managed by general practitioners (GPs) in primary care settings, although estimates of this proportion vary widely depending on the definitions used.[4] Research suggests that the reasons patients choose to attend an emergency department with problems suitable for General Practice include: the perceived urgency of the situation, the belief that they need care only available in hospitals, the convenience of obtaining care at any time without an appointment, barriers to accessing general practice, and a lack of awareness of available primary care services.[5-7]

Several different policy initiatives have been proposed to address rising ED demand, and to allow EDs to focus on patients with the most urgent need.[8-10] These responses fall into three main categories: a triage step before patients attend EDs, such as a telephone advice line or "streaming" at the front door of the ED to direct patients to alternative services; better provision



For peer review only - http://bmjopen.bmj.com/site/about/guidelines.xhtml

of alternative services (such as nurse-led walk-in services and Urgent Treatment Centres); improved access to GP services for people attending EDs. The latter approach can be achieved either by co-locating GP services alongside EDs at hospital sites, or by employing GPs to work within EDs to see selected patients. It has been suggested that GPs in or alongside the ED have the potential to improve patient care, and to reduce waiting times, unnecessary investigations, hospital admission rates, and costs,[11] but evidence to substantiate these claims is limited.[12-16] The introduction of these services was accelerated in 2017, when the UK government provided £100million of capital funding to support hospitals in England to provide a GP working in or alongside the ED,[17-19] as part of a comprehensive plan to reduce the growth of lower acuity patients attending EDs.[10] The aim of our research was to examine: i) the effect of General Practitioners working in or alongside the Emergency Department (GPED) on patient outcomes and experience; ii) the associated workforce and system impact; iii) the differential effects of different GPED service models.

## Methods

### Design

We completed a mixed methods study including interviews with service leaders and NHS managers, in-depth case studies and a retrospective observational analysis of routinely collected national data. This approach enabled us to obtain a service-wide understanding of the impact of GPED on the urgent care system, the associated workforce and patient care.[20-23] Details of the study methodology have been published previously.[24]

Ethics committee approval was obtained from East Midlands – Leicester South Research Ethics Committee (ref: 17/EM/0312); University of Newcastle Ethics Committee (Ref: 14348/2016) and the Health Research Authority (IRAS: 230848 and 218038).

### Theoretical approach

We drew on Normalisation Process Theory (NPT), which has been widely used to understand how and why things do or do not become embedded into routine practice.[25] The theory recognises that implementation is complex, and that successful 'adoption' is dependent on whether people are prepared to invest in incorporating an intervention into their everyday 'work.' As such, NPT views implementation as a turbulent and unpredictable process which the theory seeks to understand through its four core constructs of coherence, cognitive participation, collective action and reflexive monitoring (Box 1).[25,26]



For peer review only - http://bmjopen.bmj.com/site/about/guidelines.xhtml

**Box 1: The four core constructs of NPT, adapted for use in the GPED study**

| |
|---|
| **Coherence:** Do staff understand why GPED has been implemented? |
| **Cognitive participation:** Are staff engaged and committed to GPED, and what are the factors that promote and/or inhibit this commitment? |
| **Collective action:** Are participants using GPED and what are the factors that promote and/or inhibit them from using GPED? |
| **Reflexive monitoring:** Have staff appraised GPED and its impact on practice? |

NPT enabled us to integrate our qualitative and quantitative data; examining the extent to which GPED had become a part of routine practice and highlighting the related impact on patients and staff.

## Qualitative data collection and analysis

Qualitative data collection consisted of non-participant observation of 142 individual clinical encounters and 467 semi-structured interviews with key stakeholders (policymakers, service leaders, ED staff, General Practitioners, patients and carers). Qualitative data was distributed across 64 NHS EDs in England, including 10 in-depth case study sites. Qualitative data collection explored the impact of GPED from the perspectives of key stakeholders as well as the policy's background and factors affecting implementation. Following initial familiarisation and independent coding, the qualitative team, through a series of roundtable discussions and workshops with our patient collaborators, developed a coding framework (additional file 1). The coding framework, in conjunction with pen portraits[27] of our ten case sites, was used to facilitate cross-case comparisons and formed the basis of our main thematic analysis.[28] Initial analysis identified ten key themes: contested policy, eight 'domains of influence' which our stakeholders predicted would be affected by GPED (performance against the four hour target; use of investigations; hospital admission; patient outcome and experience; service access; staff recruitment and retention; workforce behaviour and experience; resource use), and structural implementation, relating to site level responses to the introduction of GPED. A separate paper reporting the eight domains of influence and how they were generated has been published previously.[29] Qualitative data collection is summarised in table 1.



For peer review only - http://bmjopen.bmj.com/site/about/guidelines.xhtml

For peer review only



For peer review only - http://bmjopen.bmj.com/site/about/guidelines.xhtml

Table 1 Qualitative data collection

| | **Policymakers** | **Service leaders** | | **Case sites** | | |
|---|---|---|---|---|---|---|
| | Time 1 | Time 1 | Time 2 | Time 1 | Time 2 | Time 3 |
| Type of data collected | Semi-structured telephone interviews | Semi-structured telephone interviews | Semi-structured telephone interviews | Semi-structured face-to-face and telephone interviews, non-participant observations. | Semi-structured face-to-face and telephone interviews | Semi-structured face-to-face and telephone interviews, non-participant observations. |
| Aim of data collection | In-depth understanding of GPED policy and implementation from key informants | Broad perspective of GPED implementation and current provision from a range of EDs | Broad perspective of GPED implementation and current provision from a range of EDs | In-depth understanding from a small number of case sites | Brief 'check in' visits to assess any interim changes in GPED services | In-depth understanding from a small number of case sites |
| Period of data collection | December 2017 to January 2018 | August 2017-September 2018 | February 2018- February 2019 | November 2017-December 2019 | June-October 2018 | November 2018-December 2019 |
| Number of EDs | Not applicable | 64 | 30 | 10 | 5 | 10 |
| Stakeholder groups and organisations represented | NHS England and Improvement, Department of health, Clinical Commissioning Groups, NHS Trusts, Royal College of Emergency Medicine, GPs | Chief Executives, Chief Operating Officers, Clinical Leads, Lead nurses and ED managers | Chief Executives, Chief Operating Officers, Clinical Leads, Lead nurses and ED managers | GPs, ED doctors (juniors, registrars, consultants), Nurses (streaming, triage, emergency nurse practitioner), patients and carers | GPs, ED doctors (juniors, registrars, consultants), Nurses (streaming, triage, emergency nurse practitioner), patients and carers | GPs, ED doctors (juniors, registrars, consultants), Nurses (streaming, triage, emergency nurse practitioner), patients and carers |
| Total number of participants | 10 policymakers | 57 service leaders | 26 service leaders | 124 health professionals 94 patients/ carers<br>83 non-participant observations. | 20 health professionals | 82 health professionals, 54 patients/carers, 59 non-participant observations. |

*GPED: General Practitioners working in or alongside the Emergency Department; ED: Emergency Department; GP: General Practitioner.*



For peer review only - http://bmjopen.bmj.com/site/about/guidelines.xhtml

For peer review only



For peer review only - http://bmjopen.bmj.com/site/about/guidelines.xhtml

## Quantitative data and analysis

We completed a retrospective observational analysis of routinely collected Hospital Episode Statistics (HES) data between April 2018 and March 2019 from the 40 English hospitals which were able to provide complete data on times of day when GPED services were available. Differences in GPED service availability between EDs at the same time of day were used to assign patients quasi-randomly to treatment or control groups based on local service availability. Outcomes measured were: percentage of patients discharged within four hours of arrival; ED attendances that resulted in hospital admission; patients who left without being seen; unplanned re-attendance at the ED within 7 days; 30 day mortality; non-urgent ED attendances (described previously as 'unnecessary' and identified using a defined methodology);[30] volume of ED attendances. Each outcome was analysed separately using two-way fixed effects. Outcomes for patients attending different EDs at the same time of day were compared, exploiting variation in the timing of availability of GPED within the day at different EDs. Further details of this analysis have been published previously.[31 32] The potential net cost savings were explored using a comparative approach based on the results of this analysis.[32] We also conducted a survey of the GPED workforce at our 10 case sites, however, as these results did not materially alter our overall findings they are not reported here.[32]

## Mixed methods analysis

In addition to individual quantitative and qualitative analyses, we conducted higher-level synthesis to integrate the study findings using a triangulation protocol that combined different methods to gain a more complete picture.[33] Quantitative findings were grouped under the qualitative themes described above (see table 2 for a summary of qualitative and quantitative data integration). We then mapped our study findings onto the four core constructs of NPT.[25] Given the inter-related nature of the NPT constructs this process was undertaken by two researchers (JA and AS).

*Table 2 Qualitative and quantitative data integration*

| Theme | Qualitative | Quantitative |
|---|---|---|
| **Contested policy** | Qualitative interviews with policymakers and service leaders, health professionals, patients and carers. Non-participant observation | |



For peer review only - http://bmjopen.bmj.com/site/about/guidelines.xhtml

| | | |
|---|---|---|
| **Performance against the four-hour target** | Qualitative interviews with policymakers, health professionals, patients and carers. Non-participant observation | HES data: percentage of patients discharged within four hours of arrival |
| **Use of investigations** | Qualitative interviews with policymakers, health professionals, patients and carers. Non-participant observation | |
| **Hospital admissions** | Qualitative interviews with policymakers, health professionals, patients and carers. Non-participant observation | HES data: ED attendances that resulted in hospital admission |
| **Patient outcome and experience** | Qualitative interviews with policymakers, health professionals, patients and carers. Non-participant observation | HES data: patients who left without being seen<br>HES data: Unplanned re-attendance at the ED within 7 days<br>HES data: 30 day mortality |
| **Service access** | Qualitative interviews with policymakers, health professionals, patients and carers. Non-participant observation | HES data: non-urgent (described previously as 'unnecessary') ED attendances<br>HES data: Volume of attendances |
| **Staff (recruitment, retention)** | Qualitative interviews with policymakers, health professionals, patients and carers. Non-participant observation | |
| **Workforce (behaviour, experience)** | Qualitative interviews with policymakers, health professionals, patients and carers. Non-participant observation | |



For peer review only - http://bmjopen.bmj.com/site/about/guidelines.xhtml

| | | |
|---|---|---|
| **Resource use** | Qualitative interviews with policymakers and service leaders, health professionals, patients and carers. Non-participant observation | |
| **Structural implementation** | Qualitative interviews with policymakers, health professionals, patients and carers. Non-participant observation | |

*ED: Emergency Department; HES: Hospital Episode Statistics.*

### Patient and public involvement

Patients and members of the public were involved throughout the development and delivery of this research. We formed a group of ten public contributors with a wide variety of experiences of ED services. Throughout the study, the group were involved in regular workshops and meetings where they were asked to assist in interpreting the qualitative and quantitative data and support the development of our mixed methods synthesis. For instance, our lay contributors highlighted the central role played by the streaming nurse in GPED, which led to a further analysis of qualitative data surrounding streaming that has been published previously.[34] Two members of the group were also full members of the Study Steering Committee.

## Results

Table 3 shows how the themes from our qualitative and quantitative data map onto the four constructs of NPT.

For peer review only

*Table 3 Summary of qualitative themes with integrated quantitative findings* mapped onto the four constructs of NPT.[35]

| NPT component | Questions to consider within the NPT framework | Key findings |
|---|---|---|
| **Coherence** – do stakeholders understand why GPED has been implemented? | Does GPED have a clear purpose and did participants have a shared sense of its this purpose?<br>Will GPED fit with the overall goals and activity of the organisation?<br>Is it clearly distinct from other interventions?<br>What benefits will the intervention bring and to whom? | ***Contested policy***<br>• The implementation of GPED was considered rushed, and to be based on conflicting guidance.<br>• Some stakeholders had difficulty understanding how GPED differed from other previously unsuccessful attempts to introduce GPs into the ED.<br>• It was uncertain how GPED, or the associated capital funding initiative, differed from previous and existing interventions.<br>• Variations in local context, ED demand and existing GP services in the ED resulted in GPED being interpreted and implemented differently.<br>***Domains of influence***<br>• GPED is difficult to describe, distinguish from other interventions and participants do not have a shared sense of its purpose.<br>• Stakeholders disagreed on the potential impacts of GPED, with positive, neutral or negative effects predicted for the majority of the eight identified domains of influence: 1) Performance against the four-hour target; 2) Use of investigations; 3) Hospital admissions; 4) Patient outcome and experience; 5) Service access; 6) Staff recruitment and retention, 7) Workforce behaviour and experience; 8) Resource use. |
| **Cognitive participation** - are people committed to using GPED and what are the factors that promote and/or inhibit this commitment? | Did stakeholders see the point easily?<br>Were stakeholders prepared to invest time, energy and work in GPED? | ***Contested policy***<br>• There was doubt whether GPED, as a single initiative, could fix complex problems in the healthcare system.<br>• GPED policy development was criticised, as was the fact that it was based on limited evidence and patient and clinical consultation. This reduced stakeholders' commitment to ensuring it was embedded into routine practice. |
| **Collective action** - are people using GPED and what are the factors that promote and/or inhibit them from using GPED? | What effect will GPED have on the ED and health service?<br>How will the intervention affect the work of patients and staff?<br>Will staff require further training? | ***Service Access***<br>• Despite reports that GPs have been working in the ED for some time, only a small number of patients reported using GPED previously and expected to be streamed to GPED.<br>• Staff were concerned that GPED may create 'easy access to a GP', encouraging people to attend.<br>• Staff were concerned that patients attended the ED "inappropriately", and considered poor health literacy to affect how patients use GPED.<br>• GPED and 'Urgent Care' were considered confusing to patients and made navigating services more challenging.<br>• Analysis of HES data identified no significant impact on: volume of ED attendances; number of non-urgent (described previously as 'unnecessary') attendances |



For peer review only - http://bmjopen.bmj.com/site/about/guidelines.xhtml

| | | |
|---|---|---|
| | | ***Staff recruitment and retention***<br>• Staffing issues posed a major threat to the successful implementation and adoption of GPED.<br>• Nursing shortages and a lack of experienced nurses made the staffing of streaming services challenging.<br>• Streaming may change the role of nurses and divert them away from core ED work, making GPED settings less attractive. The psychological and physical impact of streaming may negatively affect nurses' work and willingness to invest energy and time in GPED.<br>• GPED may draw GPs away from traditional General Practice. ED staff vacancies created issues in the recruitment of ED and GP staff.<br>• To overcome recruitment issues, GPED needs to be viewed as an attractive place to work.<br>• The training and educational benefits that junior doctors may receive from working alongside GPED models were considered valuable, and may make them more committed to ensuring GPED is embedded into routine practice. |
| | What impact will it have on division of labour, resources, power and responsibility between different professional groups?<br>What are the factors that promote and/or inhibit them from using GPED? | ***Use of investigations***<br>• There was a lack of consensus as to whether GPED models should give GPs access to diagnostic testing, reflecting differing interpretations of the purpose of GPED and varying local needs. This caused tension between GP and ED staff and may make staff less likely to invest their time and energy into GPED.<br>***Workforce behaviour and experience***<br>• Good communication, trust and confidence between streaming staff and GPs are pivotal to the effectiveness of GPED.<br>• Staff were concerned about patients who attend the ED with conditions that could be treated in General Practice, but had different perceptions of what constitutes a 'GPED appropriate patient.'<br>• Tensions between GPs and staff responsible for streaming decisions were common and reflected different attitudes to risk as well as staff members (ED and GP) protecting their own working environment – staff streamed patients to GPED, or back to ED during busy periods, to ease their respective workloads.<br>• Streaming protocols were developed to try to standardise streaming decisions and GPED acceptance criteria, however these were not consistently disseminated or followed.<br>***Structural implementation***<br>• Several implementation issues also affected the extent to which staff were able to embed GPED into their routine practice including structural support within the site, ensuring integrated information technology systems between ED and GPED and influencing factors relating to the GP's role such as ensuring a positive working environment and giving GPs access to investigations, where appropriate. |
| **Reflexive monitoring - **have people appraised GPED and its impact on practice? | Will it be clear what effects the intervention has had? | ***Performance against the 4 hour target and hospital admissions***<br>• There was no significant impact on the proportion of patients meeting the four hour target, or on the number of attendances resulting in a hospital admission. |



For peer review only - http://bmjopen.bmj.com/site/about/guidelines.xhtml

For peer review only

| | | |
|---|---|---|
| | How are users likely to perceive the intervention once it has been in use for a while?<br>Is it likely to be perceived as advantageous for patients or staff? | • Variations in site-specific patient mix, GPED models and whether patients streamed to GPED were included in ED reporting statistics, combined with other factors that influence ED performance, may have contributed to the apparently limited effects of GPED.<br>***Resource use***<br>• Any possible cost savings due to reduced reattendances were much smaller than the cost of providing the service itself.<br>***Patient outcome and experience***<br>• Most patients saw the value of GPs working in or alongside the ED as long as they received appropriate care.<br>• Staff felt that GPED may negatively affect patient flow.<br>• There was no significant impact on the following performance indicators in the HES analysis: left without being seen; 30-day mortality; re-attendance to the same ED within seven days. |

*GPED: General Practitioners working in or alongside the Emergency Department; ED: Emergency Department; GP: General Practitioner; HES: Hospital Episode Statistics.*

For peer review only



For peer review only - http://bmjopen.bmj.com/site/about/guidelines.xhtml

### Coherence – do stakeholders have an understanding of why GPED was implemented?

For a health policy to be adopted into routine practice, there needs to be a shared sense of its purpose. Many stakeholders understood that GPED was being introduced as a direct response to rising pressures in EDs and as a potential mechanism for improving ED performance. Despite this, all stakeholder groups suggested that GPED was a rushed policy that lacked clear and consistent guidance. The fact that the policy was believed to originate largely from discussions between the Secretary of State for Health and NHS England, leading to "top down" implementation, and the lack of evidence supporting the clinical and cost-effectiveness of GPED were further causes of concern.

*"I think it adds to the mix. I think that it was not a very well thought through policy decision … It was never part of the urgent, the care, the Keogh review of urgent emergency care to have GPs in ED. Now that review focused much more on NHS 111 and also trying to create consistency … So having GPs in ED, was outside of that policy strand. So, and it was dropped in a very, at very great speed and without a great deal of thought." (Interview with service leader)*

The decision to introduce GPED nationally was also based on the perceived success of a GPED service that had been implemented at a single NHS site - Luton and Dunstable (L&D). The rationale for choosing L&D as the national exemplar over other high-performing EDs was unclear, particularly given that it was difficult to determine whether the perceived success of L&D was due to GPED or the simultaneous introduction of other initiatives within the organisation. Associated with this were concerns that GPED failed to acknowledge local context and variations in demand for ED services, varying patient populations and pre-existing or prior attempts to implement GPED services.

*"You know, it isn't a sufficient evidence base to work from. You could have looked at the North East of England, I'm taking this call just now and said, you know, six of the top ten performers nationally sit in the North East, alright, and that tells us something about the system... and I think that, if we're going to use examples as a way of developing policy, that would have been a better way of looking at it." (Interview with Policymaker)*

This led to stakeholders questioning the generalisability of the national policy, and as a result GPED was interpreted differently and a range of models were implemented throughout the NHS in England.[36 37]

*"Whilst we started with a very clear - here's the Luton model, it became, obviously when trusts came to implement it locally that due to various circumstances that were very specific to their trust and their community, the Luton model just wasn't appropriate. So, I think what we've ended up with is a range of different models. So, you couldn't look at GP streaming*



For peer review only - http://bmjopen.bmj.com/site/about/guidelines.xhtml

*and say what we've got in place now is the same in every trust in the country because there's almost certainly ... there's huge variation in practice around how they're running." (Interview with policymaker)*

There was widespread disagreement at an individual, stakeholder and organisational level about the purpose and potential impact of GPED. Despite disagreeing about the 'direction of effect,' stakeholders agreed on the areas of the healthcare system and patient care that GPED was most likely to affect. We categorised these as 8 'domains of influence' (Tables 2 and 3).[29]

### Cognitive participation – are people committed to using GPED and what are the factors that promote and/or inhibit this commitment?

The way in which GPED policy was designed and implemented, along with challenges in translating a national policy to meet local service and population needs, caused some to view GPED as a 'sticking plaster solution' to ED pressures. For many, the rise in ED attendances was driven by wider, more complex issues across health and social care, which were often deemed to be the result of deficiencies elsewhere in the system. As a result, there was doubt that a single initiative such as GPED could provide the solution. This lack of buy-in from stakeholders was reflected during interviews with service leaders and policymakers where alternative solutions for improving ED performance were proposed. For example, investment in social care and mental health services were considered to have a greater potential for impact.

*"Because it [GPED] is cheaper than re-investing in social care. Preventing inappropriate admissions is right, but it doesn't solve all the problems in primary care - those patients that do need to be seen and do need support in the community/social care, [GPED] is not a long-term solution." (Interview with service leader)*

Embedding GPED into existing practice requires commitment from key stakeholders. Emphasis was placed on the importance of streaming nurses and GPs working together to stream patients from ED to GPED. Despite many sites trying to ensure consistency through the development of streaming protocols, the challenges of disseminating and adhering to these protocols, reliance on locum and/or part-time GPs and frequent rotation of streaming nurses meant that the definition of a patient suitable for GPED varied between and within professional groups. This, combined with the cultural differences in how GPs and ED clinicians work, and their inherently different approaches to risk, was a source of tension that in some cases resulted in patients not being accepted by GPED and sent back to ED.

*"It [streaming criteria] should be fixed, but, as I said, depending on who you speak to, it does waver slightly on what practitioners and GPs are willing to see. So, it's a bit of a grey area*



For peer review only - http://bmjopen.bmj.com/site/about/guidelines.xhtml

*really. It depends who you're working with really. I don't . . . yes. So, it's not fixed. It should be really." (Interview with Advanced Nurse Practitioner at Case site Redwood).*

Whether GPED models gave GPs access to investigations such as x-rays and blood tests varied across case sites and reflected the different interpretations of the purpose of GPED and varying local contexts. Some individuals considered giving GPs access to investigations and diagnostic tests as crucial to the model's effectiveness by supporting GPs to treat a broader range of patients and refer to inpatient specialties. However, others felt that doing so asked GPs to work beyond their clinical competency – some staff felt that there was a shortage of GPs with the skills required to interpret some ED diagnostic tests, and an upskilling of the GP workforce would therefore be required. As a result, some GPs were asked to work as they would in general practice, whilst other services preferred those with prior ED experience.

*"Actually, looking at X-rays and ECGs is, it becomes a bit of a, a dying art in General Practice, if you're not looking at those sorts of things on a daily basis, and what we provide again is allowing GPs the ability to keep those sort of clinical skills up and running, when I think that, and I think that's the attractiveness about doing this." (Interview with Urgent Care Centre clinical lead at case site Teak)*

### Collective action – are people using GPED and what are the factors that promote and/or inhibit them from using GPED?

At the time that GPED was introduced, general practice in England was facing a significant workforce crisis. This posed a real challenge both in terms of ensuring that EDs were able to recruit GPs to work in GPED and ensuring that in doing so workforce shortages elsewhere in the system were not exacerbated. Site staff suggested that to facilitate the recruitment of GPs, emphasis should be placed on ensuring that GPED was considered an attractive place to work and on supporting GPs to work within the scope of their practice. However, whether GPED was viewed as a positive role depended on the individual GP. For instance, whilst GPED may be appealing to those who wish to expand their work beyond traditional general practice, the scope, acuity and shift-based working that are typical of the ED may contradict why many individuals chose to become a GP in the first place.

*"what appeals to me is that I can do a bit of acute general medicine, trauma etc. and I'm trained in that but equally, I can also lapse into what was my comfort zone … and that works really well whereas when I'm feeling a bit more sort of "right, come on, you know, I can get into resus and I can learn a new thing' and I really enjoy that." (Interview with GP at case site Juniper).*

Ensuring that streaming is undertaken by experienced streaming nurses was also considered pivotal to an effective GPED service. However, nursing shortages, the psychological and



For peer review only - http://bmjopen.bmj.com/site/about/guidelines.xhtml

physical burden of streaming on nurses and the potential for streaming to divert nurses away from their routine ED work meant that recruiting nurses to streaming roles was challenging. [34]

*"The GP feels that one of the problems with the model, is that there is a need for experienced triage nurses in order for it to work, but the department has a high turnover of nursing staff and has difficulty retaining staff. There are only a couple of appointed nurses who have the experience required." (Interview with ED Consultant at case site Redwood).*

Our findings also identified several other factors that may promote or inhibit how staff use GPED, and the extent that it becomes embedded into routine practice (table 4). These were categorised as those relating to; workforce behaviour and experience (communication, trust and role-based cultural differences) and streaming and implementation issues (streaming protocols, inter-professional relationships and structural support).

Service leaders and site staff were concerned that giving patients 'easy access' to a GP, in a climate where general practice appointments may be difficult to obtain, could encourage patients to attend the ED rather than their own GP. Staff were particularly critical of patients for what they considered 'inappropriate ED attendance' (i.e. attending the ED when alternative services would meet their needs). Whilst this was largely attributed to the potentially confusing range of services available, re-organisation and re-branding of existing services and perceived low levels of health literacy making service navigation difficult for patients, there were also some patients who were accused of deliberately 'playing the system'. For example, some patients were thought to deliberately bypass their GP and attend ED to access investigations, referrals or treatments. However, the reasons that patients chose to attend ED were complex, and in some cases, those that were considered to have attended "inappropriately" had been advised to attend the ED by other healthcare professionals and services such as NHS111, a pharmacy or their own GP.

*"ED's frightened to send anything away, so everything comes in. So, I don't blame the public for attending if they can see a GP within three hours, rather than having to wait six, seven days or two weeks for an appointment. But I just wonder if it's made a demand for it, because you get people coming back to see the GP again in ED. (Interview with Nurse at case site Rowan)*

*"Patients are savvy as well, tell you what they think they want you to hear in order to get them into the service they want to be seen by." (Interview with Nurse at case site Linden).*

However, our qualitative data provided numerous examples of situations in which experienced nurses were unable to determine whether a patient's complaint should be treated by general



For peer review only - http://bmjopen.bmj.com/site/about/guidelines.xhtml

practice or the ED, suggesting that it may be unreasonable to expect patients to make the correct choice on every occasion.

*"I think it's down to, obviously, your training, but also how risk averse you are, and some people are very risk averse and will just have a much lower threshold for streaming people into ED and then also the Urgent Care Centre, rather than directing appropriately, you know, taking that risk." (Interview with Paramedic at case site Chestnut).*

Despite these concerns amongst site staff, analysis of HES data found no association between non-urgent attendances and GPED or the absolute and relative volume of attendances and GPED.[31] Despite staff believing that GPED may encourage ED use, only a small number of patients expected to see a GP, with the majority showing no awareness of GPED when interviewed. This is perhaps unsurprising given that sites often chose not to advertise GPED services to reduce the likelihood of driving an increase in ED attendances.

## Reflexive monitoring – have people appraised GPED and its impact on practice?

GPED is a complex intervention that has been introduced through a range of different models, into a complex and changing environment. EDs serve different patient populations and have different physical structures, staff mixes and care provision. In addition to this heterogeneity, the widespread uncertainty surrounding GPED operating hours and different governance arrangements across sites meant that there was variation in whether patients streamed to GPED were counted in nationally reported ED statistics. The challenges of using key performance indicators to evaluate national policies such as GPED was discussed by service leaders, who questioned their utility and described indicators such as the target that 95% of patients attending the ED should be admitted, transferred or discharged within four hours as 'blunt tools' for evaluating impact.

*"Yeah, I think that's really important, I think given the way the hospital performs with the Government's four-hour target, I think it's a source of pride for the hospital for the Chief Exec." (Interview with ED Consultant at case site Linden).*

Our quantitative analysis showed no statistically significant improvement in a range of key performance indicators across several domains of influence including the "four-hour target", hospital admissions and patient outcomes and experience (patients leaving the ED without being seen and mortality at 30 days after an ED attendance). We did observe that GPED reduced the probability of unplanned re-attendance within seven days by 3.2% (OR: 0.968, 95% CI: 0.95 to 0.99), which equates to approximately 300 fewer re-attendances per year for



For peer review only - http://bmjopen.bmj.com/site/about/guidelines.xhtml

an average ED in England. After adjustment for multiple testing, however, this difference was no longer statistically significant, and was also not judged to be clinically significant. Possible cost savings associated with reduced reattendances (£30-37,000 per ED per year) were heavily outweighed by the cost of GPED services. In the hospitals for which we had data, the average length of time of operation of a GPED service was 11.1 hours per day. Assuming only one GP is present and including salary costs of the GP alone (potentially a substantial underestimate), this amounts to around £454,000 per ED per year. As a result, current GPED models do not appear to be an efficient use of healthcare resources.[32]

*"I don't necessarily think it is a bad thing to have it, but it provides marginal gains, and those marginal gains are, happening at a very high capital cost and an ongoing staffing cost and looking at the NHS budget as a whole, I think it's a shocking waste of money." (Interview with ED consultant at case site Juniper)*

The majority of patients we interviewed valued GPED and considered it beneficial to have GPs in EDs. Patients were aware that GPED may relieve pressure on the ED, ensuring emergency doctors can deal with the "real emergency cases" and were indifferent to the type of health professional that they saw as long as they received appropriate care. Similarly, the "four-hour target" was not a priority for patients, with many explaining that they were happy to wait longer as they understood that they were guaranteed to be seen and were waiting because priority was given to higher acuity patients. Despite this, staff raised concerns that GPED could negatively impact patient flow, as patients are required to disclose clinical information on multiple occasions before seeing a GP, which may create a backlog.

## Discussion

The GPED study was commissioned to evaluate the impact of GPs working in or alongside EDs; a national policy implemented in response to rising pressures on EDs in England. GPED had no effect on a range of routinely collected ED performance measures. Despite considerable concern from health professionals that GPED may actually increase demand, we found no significant effect of GPED on ED attendances or reattendances within 7 days. This was supported by our qualitative analysis; most of the patients that we interviewed were unaware of GPED and had not changed their behaviour as a result. We observed confusion amongst patients, staff, service leaders and participating NHS organisations as to the purpose of GPED, with a prevailing view that the main drivers of ED workload may be more related to an ageing population, high inpatient bed occupancy and a shortage of social care[38] than attendances by patients suitable for management in traditional general practice.



For peer review only - http://bmjopen.bmj.com/site/about/guidelines.xhtml

Our findings suggest that GPED implementation was highly sensitive to local context. This is consistent with other evaluations of urgent and emergency care initiatives.[39 40] For example, Walk-In Centres were introduced to shift demand from EDs and out-of-hours General Practice. However, their introduction was found to have little overall impact, and any impacts that were found were hugely variable and sensitive to local context.[39]

The GPED study shows that even when a policy is mandatory and supported by dedicated capital funding, this does not guarantee successful or uniform adoption. Our findings highlight the complexities of translating policy into practice, and the importance of considering the extent that a government-led policy can be delivered at a local level. Previous evidence suggests that a common response to national policy is local adaptation, which can in turn lead to the implementation of different innovations to those that are originally proposed.[20] We found evidence of this, as interviewees often described a range of approaches to GPED that sometimes opposed the high-level policy messages that accompanied the provision of capital funding.

Our qualitative data also identified a range of factors that can facilitate implementation. We present these as a series of 'success factors' which may inform how services choose to implement future GPED models; or adapt existing ones (Table 4). At several of our case study sites, these fundamentals had been overlooked and the result was a weaker GPED service. However, it is important to note that even if all these 'success factors' are implemented, our findings do not present evidence that the resulting GPED service would have a positive impact on ED performance indicators or be cost-effective.

*Table 4 Success factors for the introduction of GPED.*

For peer review only

| Success factor | How can this be addressed? |
|---|---|
| **Streaming** | No single model for effective streaming was identified. The factors listed below should be considered when developing future streaming models. |
| *The experience and seniority of streaming nurses* | Effective streaming requires high levels of clinical knowledge, critical thinking, clinical decision-making and balancing clinical risks. Streaming should be undertaken by senior nurses. |
| *The skills, confidence and abilities of GPs* | Professional groups had different opinions as to what can be considered a "GP appropriate" patient. To alleviate tension between staff there needs to be a shared understanding of streaming protocols and an awareness of the skills and scope of practice of GPs. Recruiting experienced and clinically knowledgeable GPs who are willing to adapt and see a broader range of patients is helpful. |
| *Inter-professional relationships* | Trust and confidence between professional groups is essential. Co-location does not automatically ensure collaboration. Individuals naturally work within professional norms. Effective communication and common goals mitigate tension. |
| *Streaming protocols* | Stakeholder clinicians (including streamers and GPs) should be involved in the development and regular review of protocols. These should be effectively communicated to all relevant practitioners. For streaming to be effective, streamers may need to deviate from protocols based on their clinical judgment. Staff should be supported to do this, while also considering strategies to mitigate against inappropriate deviation which may negatively impact patient care. |
| *Streaming safety* | Safety concerns limit the effectiveness of streaming strategies and sources of support are needed to ensure staff feel confident in their decision making.<br>Clinicians should be involved in the development and regular review of protocols. These include effective pathways for managing deteriorating patients and returning streamed patients back to the ED when necessary. Consider ways to make the streaming process clearer for patients to navigate, to reduce repetition and patient frustration. Onward referrals were often a pinch point in the system, with patients at risk of increased waiting times or being overlooked. Guidance and support for streaming |



For peer review only - http://bmjopen.bmj.com/site/about/guidelines.xhtml

| | |
|---|---|
| | nurses experiencing complaints processes, litigation or professional registration issues should be provided. |
| **Staffing** | Less reliance on locum GPs and ensuring GPED shifts are covered consistently, and communicated effectively, promotes consistency. Recruitment of highly experienced and clinically knowledgeable GPs who are willing to adapt their practice to take on a broader range of work<br><br>Consider retention strategies to support current streaming nurses and to futureproof streaming by training and retaining adequate numbers of suitably experienced nurses. Streamers should be supported by their professional colleagues. Implement strategies to mitigate against burnout, prevent overload from additional responsibilities and positive promotion of streaming roles to make them attractive to nurses. |
| **Leadership** | Involve staff of all grades and from all key professional groups in the development and implementation of service planning, organisation and protocol development to counteract feelings of top-down change and encourage buy-in and support. |
| **Physical environment** | Consider the impact of the physical environment, e.g. privacy at the streaming desk, safety of both staff and patients in isolated or exposed streaming areas, and for GPs located away from the ED and in off-site Hubs. Inadequate space can lead to overcrowding. Patients who have to queue more than can become confused and frustrated. Consider where GPs are placed to avoid feeling isolated and separated from the ED. |
| **Integrated IT systems** | Effective, easy to use and joined up information technology systems between ED, GPED and General Practice are essential for a safe working environment. |
| **Structural support** | Support for streamers should include specific training, regular supervision, audit and feedback. GPED models and streaming services should be planned and organised with involvement and buy-in from key stakeholders including streaming nurses and GPs. |

*GPED: General Practitioners working in or alongside the Emergency Department; ED: Emergency Department; GP: General Practitioner.*

For peer review only

GPED is a new policy initiative, which has been evaluated by two large NIHR commissioned research studies (HS&DR Projects 15/145/04 and 15/145/06).[24 32 37 41] Further research evaluating its impact is therefore not recommended until the policy has been given time to embed into routine practice. Instead, priority should be given to evaluating existing performance measures and developing new, rapid methods to inform the development, implementation and evaluation of similar health policy initiatives (Box 2).

**Box 2: Implications for future research**

1. The utility and completeness of key performance indicators and national routine data sets limit the ability to evaluate the impact of complex health initiatives. Further work is needed to review existing measures and data sets. When undertaking this work, patients and clinicians should be consulted to ensure that measures of 'success' include factors that are important to all stakeholder groups.
2. The relationship and interface between general practice and secondary care is crucial to the future delivery of urgent and emergency care. Research to explore this relationship and different approaches to risk will inform future models of service development and delivery in the context of rising healthcare demand.
3. We identified particular ambiguity and uncertainty in relation to streaming in the ED. Further research to clarify the optimal approach to streaming in terms of patient outcome, safety and experience, and the wider implications of streaming on staff experience, is warranted.

only

## Strengths and limitations

We adopted a mixed-methods approach which consisted of 'big qualitative' data collection (413 interviews and 142 individual observations of clinical encounters) and quantitative analysis of national data sets to explore the impact of GPED. This approach, and the decision to interpret our study findings using NPT, provided us with an in-depth understanding of the impact of GPED. This highlighted the complex interplay of political, workforce and social factors that affect successful adoption of a health policy into routine practice.

Our data apply to England only, and so may not be generalizable to other countries and healthcare settings. In our quantitative analysis, it was not possible to identify from available data which staff members assessed and treated individual patients, so we could not separate patients treated by GPs from those treated by other ED staff to directly compare GP services



For peer review only - http://bmjopen.bmj.com/site/about/guidelines.xhtml

to traditional models of care. We relied primarily on measures of general ED performance, such as attendances, patient flow and waiting times. We were also limited in our ability to collect data from the general practice and urgent care systems surrounding our case study sites, which significantly limited our ability to evaluate quantitatively the effect of GPED on the wider healthcare system. Our qualitative case study sites were selected purposively to be as representative as possible. However, participation by sites, and from staff and patients during data collection, was voluntary and so is unlikely to be exhaustive.

## Conclusion

Implementation of General Practitioners working in or alongside the ED was highly subject to local context and micro-level influences. However, we found no consistent evidence of improvements in patient outcome or experience. This is summed up by our public contributors, who following presentation of the final study findings concluded:

*"GPED is not effective and should only be used where specific circumstances indicate that it may play a positive role."*

**Contributor and guarantor information**

AS and JA drafted the paper and are joint first authors for the manuscript. JB and CS helped to draft the manuscript. JA, JB, KB, SC, TD, AG, NG, SP, CS, SV had the initial research idea and obtained funding for this study. Qualitative data collection and analysis were undertaken by HA, JA, HL, AS. Quantitative data collection and analysis were undertaken by: KB, TD, JG, NG. Mixed methods analysis was undertaken by: JA and AS. All authors critically reviewed, revised and approved the final manuscript. JB is the study guarantor. The guarantor (JB) accepts full responsibility for the work and the conduct of the study, had access to the data, and controlled the decision to publish. The corresponding author attests that all listed authors meet the authorship criteria and that no others meeting the criteria have been omitted.

**Copyright/license for publication**

"The Corresponding Author has the right to grant on behalf of all authors and does grant on behalf of all authors, a worldwide licence to the Publishers and its licensees in perpetuity, in all forms, formats and media (whether known now or created in the future), to i) publish, reproduce, distribute, display and store the Contribution, ii) translate the Contribution into other languages, create adaptations, reprints, include within collections and create summaries,



For peer review only - http://bmjopen.bmj.com/site/about/guidelines.xhtml

extracts and/or, abstracts of the Contribution, iii) create any other derivative work(s) based on the Contribution, iv) to exploit all subsidiary rights in the Contribution, v) the inclusion of electronic links from the Contribution to third party material where-ever it may be located; and, vi) licence any third party to do any or all of the above.

## Patient consent for publication

Not applicable

## Data availability statement

The deidentified patient-level data used for the quantitative component of this study, including information on mortality, were released by the data holders (NHS Digital, Office for National Statistics) under specific data sharing agreements and only for the purpose of this study. The data sharing agreements do not permit further sharing or publication of the data. Interested parties may seek to obtain data directly from the relevant data holders. Hospital Episode Statistics (HES) data are copyright 2018-2019, reused with the permission of NHS Digital through Data Sharing Agreement NIC-84254-J2G1Q. The data about the hours a general practitioner services was operating in emergency departments was collected by the authors specifically for this project. The authors are not able to place the original data into the public domain. The qualitative data we have acquired will not be available as our ethical approval does not permit the sharing of the entire data set.

## Ethics statements

## Ethical approval

Ethical approval was obtained from East Midlands – Leicester South Research Ethics Committee (ref: 17/EM/0312); University of Newcastle Ethics Committee (Ref: 14348/2016) and the Health Research Authority (IRAS: 230848 and 218038). All participants provided informed consent before taking part in the qualitative study.

## Transparency declaration

The lead authors (AS, JA) and manuscript guarantor (JB) affirm that the manuscript is an honest, accurate and transparent account of the study being reported; that no important aspects of the study have been omitted; and that any discrepancies from the study as originally planned (and, if relevant, registered) have been explained.



For peer review only - http://bmjopen.bmj.com/site/about/guidelines.xhtml

**Role of funding source**

This work was supported by the National Institute for Health Research (NIHR) Health Services and Delivery Research (HS&DR) Programme, (grant number 15/145/06). The funders had no role in the design and conduct of the study; the collection, management, analysis, and interpretation of the data; and the preparation, review, or approval of the manuscript. The views expressed are those of the authors and not necessarily those of the NIHR or the Department of Health and Social Care.

Heather Brant's time is supported by the National Institute for Health Research Applied Research Collaboration West (NIHR ARC West).

**Competing interests**

All authors have completed the ICMJE uniform disclosure form and declare: support from the National Institute for Health Research (NIHR) Health Services and Delivery Programme for the submitted work*; no financial relationships with any organisations that might have an interest in the submitted work in the previous three years; no other relationships or activities that could appear to have influenced the submitted work."*

Acknowledgements

The authors would like to thank the participants for their involvement in the qualitative study, and the patient and public contributors.

## References:

1. NHS Digital. Hospital Accident and Emergency Activity 2017-2018 Online: NHS Digital; 2018 [Available from: https://digital.nhs.uk/data-and-information/publications/statistical/hospital-accident--emergency-activity/2017-18 accessed 23/11/2021 2021.
2. Berchet C. Emergency Care Services. 2015 doi: doi:https://doi.org/10.1787/5jrts344crns-en
3. Morley C, Unwin M, Peterson GM, et al. Emergency department crowding: a systematic review of causes, consequences and solutions. *PloS one* 2018;13(8):e0203316.
4. Morris T, Mason SM, Moulton C, et al. Calculating the proportion of avoidable attendances at UK emergency departments: analysis of the Royal College of Emergency Medicine's Sentinel Site Survey data. *Emergency Medicine Journal* 2018;35(2):114-19. doi: 10.1136/emermed-2017-206846



For peer review only - http://bmjopen.bmj.com/site/about/guidelines.xhtml

5. Vogel JA, Rising KL, Jones J, et al. Reasons Patients Choose the Emergency Department over Primary Care: a Qualitative Metasynthesis. *Journal of General Internal Medicine* 2019;34(11):2610-19. doi: 10.1007/s11606-019-05128-x
6. Coster JE, Turner JK, Bradbury D, et al. Why Do People Choose Emergency and Urgent Care Services? A Rapid Review Utilizing a Systematic Literature Search and Narrative Synthesis. *Academic Emergency Medicine* 2017;24(9):1137-49. doi: 10.1111/acem.13220
7. O'Cathain A, Connell J, Long J, et al. 'Clinically unnecessary' use of emergency and urgent care: A realist review of patients' decision making. *Health Expectations* 2020;23(1):19-40. doi: 10.1111/hex.12995
8. College of Emergency Medicine. Acute and emergency care: prescribing the remedy. London: College of Emergency Medicine, 2014.
9. Urgent and Emergency Care Review Team, Emergency Care Intensive Support Team. Transforming urgent and emergency care services in England. Safer, faster, better: good practice in delivering urgent and emergency care. A guide for local health and social care communities. London: NHS England, 2015.
10. NHS England. Next Steps on the NHS Five Year Forward View. England, 2017.
11. Boeke AJP, van Randwijck-Jacobze ME, de Lange-Klerk EM, et al. Effectiveness of GPs in accident and emergency departments. *British Journal of General Practice* 2010;60(579):e378-e84. doi: 10.3399/bjgp10X532369
12. Cooper A, Davies F, Edwards M, et al. The impact of general practitioners working in or alongside emergency departments: a rapid realist review. *BMJ Open* 2019;9(4):e024501. doi: 10.1136/bmjopen-2018-024501
13. Ramlakhan S, Mason S, O'Keeffe C, et al. Primary care services located with EDs: a review of effectiveness. *Emergency Medicine Journal* 2016;33(7):495-503. doi: 10.1136/emermed-2015-204900
14. Turner J, Coster J, Chambers D, et al. What evidence is there on the effectiveness of different models of delivering urgent care? A rapid review: NIHR Journals Library, Southampton (UK) 2015.
15. Gonçalves-Bradley D, Khangura JK, Flodgren G, et al. Primary care professionals providing non-urgent care in hospital emergency departments. *Cochrane Database of Systematic Reviews* 2018(2) doi: 10.1002/14651858.CD002097.pub4
16. National Institute for Health and Care Excellence. GPs within or on the same site as emergency departments, 2017.
17. Iacobucci G. All emergency departments must have GP led triage by October: British Medical Journal Publishing Group, 2017.
18. HM Treasury. Spring Budget. London, 2017.
19. Department of Health and Social Care. Funds for next winter will ease pressure on accident and emergency (A&E) departments in England. London, 2017.
20. Pope C, Robert G, Bate P, et al. Lost in translation: a multi-level case study of the metamorphosis of meanings and action in public sector organizational innovation. *Public Administration* 2006;84(1):59-79.
21. Travaglia JF, Nugus P, Greenfield D, et al. Contested innovation: the diffusion of interprofessionalism across a health system. *International Journal for Quality in Health Care* 2011;23(6):629-36.
22. Caldwell SE, Mays N. Studying policy implementation using a macro, meso and micro frame analysis: the case of the Collaboration for Leadership in Applied Health Research & Care (CLAHRC) programme nationally and in North West London. *Health research policy and systems* 2012;10(1):1-9.
23. Sausman C, Oborn E, Barrett M. Policy translation through localisation: implementing national policy in the UK. *Policy & Politics* 2016;44(4):563-89.



For peer review only - http://bmjopen.bmj.com/site/about/guidelines.xhtml

24. Morton K, Voss S, Adamson J, et al. General practitioners and emergency departments (GPED)—efficient models of care: a mixed-methods study protocol. *BMJ open* 2018;8(10):e024012.
25. May CR, Mair F, Finch T, et al. Development of a theory of implementation and integration: Normalization Process Theory. *Implementation Science* 2009;4(1):1-9.
26. May CR, Johnson M, Finch T. Implementation, context and complexity. *Implementation Science* 2016;11(1):1-12.
27. Sheard L, Marsh C. How to analyse longitudinal data from multiple sources in qualitative health research: the pen portrait analytic technique. *BMC medical research methodology* 2019;19(1):1-10.
28. Braun V, Clarke V. Reflecting on reflexive thematic analysis. *Qualitative research in sport, exercise and health* 2019;11(4):589-97.
29. Scantlebury A, Brant H, Anderson H, et al. Potential impacts of general practitioners working in or alongside emergency departments in England: initial qualitative findings from a national mixed-methods evaluation. *BMJ open* 2021;11(5):e045453.
30. NHS Digital. Hospital Accident & Emergency Acitivity 2020-21, 2021.
31. Gaughan J, Liu D, Gutacker N, et al. Does the presence of general practitioners in emergency departments affect quality and safety in English NHS hospitals? A retrospective observational study. *BMJ Open* 2022;12(2):e055976. doi: 10.1136/bmjopen-2021-055976
32. Benger JB, H; Scantlebury, A; Anderson, H; Baxter, H; Bloor, K; Brandling, J; Cowlishaw, S; Doran, T; Gaughan, J; Gibson, A; Gutacker, N; Leggett, H; Liu, D; Morton, K; Purdy, S; Salisbury, C; Vaittinen, A; Voss, S; Watson, R; Adamson, J;. General Practitioners working in or alongside the emergency department: the GPED mixed-methods study. *Health Services and Delivery Research Program* In press
33. O'Cathain A, Murphy E, Nicholl J. Three techniques for integrating data in mixed methods studies. *Bmj* 2010;341
34. Anderson H, Scantlebury A, Leggett H, et al. Factors influencing streaming to General Practitioners in emergency departments: A qualitative study. *International Journal of Nursing Studies* 2021;120:103980.
35. Murray E, Treweek S, Pope C, et al. Normalisation process theory: a framework for developing, evaluating and implementing complex interventions. *BMC medicine* 2010;8(1):1-11.
36. Brant H, Voss S, Morton K, et al. Current provision of general practitioner services in or alongside emergency departments in England. *Emergency Medicine Journal* 2021;38(10):780-83.
37. Cooper A, Edwards M, Brandling J, et al. Taxonomy of the form and function of primary care services in or alongside emergency departments: concepts paper. *Emergency Medicine Journal* 2019;36(10):625-30.
38. The Kings Fund. What's going on with A&E waiting times? Online, 2020.
39. Salisbury C, Hollinghurst S, Montgomery A, et al. The impact of co-located NHS walk-in centres on emergency departments. *Emergency Medicine Journal* 2007;24(4):265-69.
40. Pope C, Turnbull J, Jones J, et al. Has the NHS 111 urgent care telephone service been a success? Case study and secondary data analysis in England. *BMJ open* 2017;7(5):e014815.
41. Edwards A. Evaluating effectiveness, safety, patient experience and system implications of different models of using GPs in or alongside Emergency Departments. *London: National Institute for Health Research (NIHR)* 2017



For peer review only - http://bmjopen.bmj.com/site/about/guidelines.xhtml

# GPED –Coding Framework

| | |
|---|---|
| NATIONAL CONTEXT | National - policy, pressures |
| LOCAL CONTEXT | Local – service landscape and population/specific local needs/considerations |
| TRUST ED & UC CULTURE | RESPOND OR RESIST whether staff are actively redirecting patients away from the ED to resist the flow or providing a service in response and recognition that patients have attended with health concerns. Where staff feel they must see patients and responsibility rests with them to provide health care. UCC/GP in ED vs primary care (differences to traditional primary care role. |
| PEN PORTRAIT DATA | Explanation of current system, patient journey through the ED, Layout, History of GPED, future plans, |
| PATIENTS REASONS FOR ATTENDING ED | Patient and staff explanations of why patients attend ED/Previous use of services e.g. have they seen/contacted service before ED |
| SERVICE LITERACY | Any discussions around appropriate/inappropriate attendances, perceived impact of service literacy and actual patient service literacy on use of GPED/ED |
| IMPLEMENTATION | Perceived Challenges and Facilitators to Implementation |
| PERCEIVED IMPACT | Perceived impact of GPED on patient safety, workforce and skills mix, staff interactions, performance/targets, views of GPED |
| EXPECTATIONS OF GPED (T1) | 'hypothesis' from stakeholders at all levels regarding their expectations of what would be the outcome of introduction to GPED. From T1 data, only prospective? |
| OTHER/MISCELLANEOUS INSIGHTS | Potential emerging insights which are outside the current framework but may be significant/to be reviewed with the WPC team regular meetings. |

StaRI
Standards for reporting implementation studies

## Standards for Reporting Implementation Studies: the StaRI checklist for completion

The StaRI standard should be referenced as: Pinnock H, Barwick M, Carpenter C, Eldridge S, Grandes G, Griffiths CJ, Rycroft-Malone J, Meissner P, Murray E, Patel A, Sheikh A, Taylor SJC for the StaRI Group. Standards for Reporting Implementation Studies (StaRI) statement. *BMJ* 2017;356:i6795

The detailed Explanation and Elaboration document, which provides the rationale and exemplar text for all these items is: Pinnock H, Barwick M, Carpenter C, Eldridge S, Grandes G, Griffiths C, Rycroft-Malone J, Meissner P, Murray E, Patel A, Sheikh A, Taylor S, for the StaRI group. Standards for Reporting Implementation Studies (StaRI). Explanation and Elaboration document. *BMJ Open* 2017 2017;7:e013318

Notes: A key concept of the StaRI standards is the dual strands of describing, on the one hand, the implementation strategy and, on the other, the clinical, healthcare, or public health intervention that is being implemented. These strands are represented as two columns in the checklist.

| | |
|---|---|
| The primary focus of implementation science is the implementation strategy (column 1) and the expectation is that this will always be completed. | The evidence about the impact of the intervention on the targeted population should always be considered (column 2) and either health outcomes reported or robust evidence cited to support a known beneficial effect of the intervention on the health of individuals or populations. |

The StaRI standardsrefers to the broad range of study designs employed in implementation science. Authors should refer to other reporting standards for advice on reporting specific methodological features. Conversely, whilst all items are worthy of consideration, not all items will be applicable to, or feasible within every study.

| Checklist item | | Reported on page # | Implementation Strategy | Reported on page # | Intervention |
|---|---|---|---|---|---|
| | | | "Implementation strategy" refers to how the intervention was implemented | | "Intervention" refers to the healthcare or public health intervention that is being implemented. |
| **Title and abstract** | | | | | |
| Title | **1** | 1 | Identification as an implementation study, and description of the methodology in the title and/or keywords | | |
| Abstract | **2** | 3 | Identification as an implementation study, including a description of the implementation strategy to be tested, the evidence-based intervention being implemented, and defining the key implementation and health outcomes. | | |
| **Introduction** | | | | | |
| Introduction | **3** | 4 | Description of the problem, challenge or deficiency in healthcare or public health that the intervention being implemented aims to address. | | |
| Rationale | **4** | 4, 5 | The scientific background and rationale for the implementation strategy (including any underpinning theory/framework/model, how it is expected to achieve its effects and any pilot work). | | The scientific background and rationale for the intervention being implemented (including evidence about its effectiveness and how it is expected to achieve its effects). |

**Commented [a1]:** This doesn't work



For peer review only - http://bmjopen.bmj.com/site/about/guidelines.xhtml

| | | | | | |
|---|---|---|---|---|---|
| Aims and objectives | **5** | 4 | The aims of the study, differentiating between implementation objectives and any intervention objectives. | | |
| **Methods: description** | | | | | |
| Design | **6** | 4 - 8 | The design and key features of the evaluation, (cross referencing to any appropriate methodology reporting standards) and any changes to study protocol, with reasons | | |
| Context | **7** | 3, 6, 8, 14, 15 | The context in which the intervention was implemented. (Consider social, economic, policy, healthcare, organisational barriers and facilitators that might influence implementation elsewhere). | | |
| Targeted 'sites' | **8** | 3, 6-8 | The characteristics of the targeted 'site(s)' (e.g locations/personnel/resources etc.) for implementation and any eligibility criteria. | | The population targeted by the intervention and any eligibility criteria. |
| Description | **9** | 4, 11-19 | A description of the implementation strategy | | A description of the intervention |
| Sub-groups | **10** | N/A | Any sub-groups recruited for additional research tasks, and/or nested studies are described | | |
| **Methods: evaluation** | | | | | |
| Outcomes | **11** | 3, 6, 7, 8, 9 | Defined pre-specified primary and other outcome(s) of the implementation strategy, and how they were assessed. Document any pre-determined targets | | Defined pre-specified primary and other outcome(s) of the intervention (if assessed), and how they were assessed. Document any pre-determined targets |
| Process evaluation | **12** | N/A | Process evaluation objectives and outcomes related to the mechanism by which the strategy is expected to work | | |
| Economic evaluation | **13** | 8 | Methods for resource use, costs, economic outcomes and analysis for the implementation strategy | | Methods for resource use, costs, economic outcomes and analysis for the intervention |
| Sample size | **14** | 6-8 | Rationale for sample sizes (including sample size calculations, budgetary constraints, practical considerations, data saturation, as appropriate) | | |
| Analysis | **15** | 3, 6, 8 | Methods of analysis (with reasons for that choice) | | |
| Sub-group analyses | **16** | 6-8 | Any a priori sub-group analyses (e.g. between different sites in a multicentre study, different clinical or demographic populations), and sub-groups recruited to specific nested research tasks | | |
| **Results** | | | | | |

| | | | | | |
|---|---|---|---|---|---|
| Characteristics | **17** | 6, 7 | Proportion recruited and characteristics of the recipient population for the implementation strategy | | Proportion recruited and characteristics (if appropriate) of the recipient population for the intervention |
| Outcomes | **18** | 11-13 14-19 | Primary and other outcome(s) of the implementation strategy | | Primary and other outcome(s) of the Intervention (if assessed) |
| Process outcomes | **19** | N/A | Process data related to the implementation strategy mapped to the mechanism by which the strategy is expected to work | | |
| Economic evaluation | **20** | 13, 18-19 | Resource use, costs, economic outcomes and analysis for the implementation strategy | | Resource use, costs, economic outcomes and analysis for the intervention |
| Sub-group analyses | **21** | 11-19 | Representativeness and outcomes of subgroups including those recruited to specific research tasks | | |
| Fidelity/ adaptation | **22** | 11-19 | Fidelity to implementation strategy as planned and adaptation to suit context and preferences | | Fidelity to delivering the core components of intervention (where measured) |
| Contextual changes | **23** | 11-19 | Contextual changes (if any) which may have affected outcomes | | |
| Harms | **24** | N/A | All important harms or unintended effects in each group | | |
| **Discussion** | | | | | |
| Structured discussion | **25** | 19-24 | Summary of findings, strengths and limitations, comparisons with other studies, conclusions and implications | | |
| Implications | **26** | 20-24 | Discussion of policy, practice and/or research implications of the implementation strategy (specifically including scalability) | | Discussion of policy, practice and/or research implications of the intervention (specifically including sustainability) |
| **General** | | | | | |
| Statements | **27** | 5, 24-26 | Include statement(s) on regulatory approvals (including, as appropriate, ethical approval, confidential use of routine data, governance approval), trial/study registration (availability of protocol), funding and conflicts of interest | | |

For peer review only

BMJ Open

# Do General Practitioners Working in or Alongside the Emergency Department Improve Clinical Outcomes or Experience? A mixed methods study

| | |
|---|---|
| Journal: | *BMJ Open* |
| Manuscript ID | bmjopen-2022-063495.R1 |
| Article Type: | Original research |
| Date Submitted by the Author: | 26-Jul-2022 |
| Complete List of Authors: | Scantlebury, Arabella; University of York Department of Health Sciences, York Trials Unit<br>Adamson, Joy; University of York, Department of Health Sciences<br>Salisbury, Chris; University of Bristol, Centre for Academic Primary Care, School of Social and Community Medicine<br>Brant, Heather; University of the West of England Faculty of Health and Applied Sciences<br>Anderson, Helen; University of York Department of Health Sciences<br>Baxter, Helen; University of Bristol, School of Social and Community Medicine<br>Bloor, Karen; University of York, Department of Health Sciences<br>Cowlishaw, Sean; The University of Melbourne, Department of Psychiatry ; University of Bristol,<br>Doran, Tim; University of York<br>Gaughan, James; University of York Department of Health Sciences<br>Gibson, Andy; University of the West of England Faculty of Health and Applied Sciences<br>Gutacker, Nils; University of York, Centre for Health Economics<br>Leggett, Heather; University of York Department of Health Sciences<br>Purdy, Sarah; University of Bristol School of Social and Community Medicine<br>Voss, Sarah; University of the West of England Faculty of Health and Applied Sciences<br>Benger, Jonathan; University of the West of England Faculty of Health and Applied Sciences |
| <b>Primary Subject Heading</b>: | Emergency medicine |
| Secondary Subject Heading: | General practice / Family practice |
| Keywords: | Health policy < HEALTH SERVICES ADMINISTRATION & MANAGEMENT, ACCIDENT & EMERGENCY MEDICINE, QUALITATIVE RESEARCH, PRIMARY CARE |
| | |

For peer review only - http://bmjopen.bmj.com/site/about/guidelines.xhtml

*I, the Submitting Author has the right to grant and does grant on behalf of all authors of the Work (as defined in the below author licence), an exclusive licence and/or a non-exclusive licence for contributions from authors who are: i) UK Crown employees; ii) where BMJ has agreed a CC-BY licence shall apply, and/or iii) in accordance with the terms applicable for US Federal Government officers or employees acting as part of their official duties; on a worldwide, perpetual, irrevocable, royalty-free basis to BMJ Publishing Group Ltd ("BMJ") its licensees and where the relevant Journal is co-owned by BMJ to the co-owners of the Journal, to publish the Work in this journal and any other BMJ products and to exploit all rights, as set out in our licence.*

*The Submitting Author accepts and understands that any supply made under these terms is made by BMJ to the Submitting Author unless you are acting as an employee on behalf of your employer or a postgraduate student of an affiliated institution which is paying any applicable article publishing charge ("APC") for Open Access articles. Where the Submitting Author wishes to make the Work available on an Open Access basis (and intends to pay the relevant APC), the terms of reuse of such Open Access shall be governed by a Creative Commons licence – details of these licences and which Creative Commons licence will apply to this Work are set out in our licence referred to above.*

*Other than as permitted in any relevant BMJ Author's Self Archiving Policies, I confirm this Work has not been accepted for publication elsewhere, is not being considered for publication elsewhere and does not duplicate material already published. I confirm all authors consent to publication of this Work and authorise the granting of this licence.*

For peer review only

# Do General Practitioners Working in or Alongside the Emergency Department Improve Clinical Outcomes or Experience? A mixed methods study

**Authors names:** Arabella Scantlebury (0000-0003-3518-2740), Joy Adamson (0000-0002-9860-0850), Chris Salisbury (0000-0002-4378-3960), Heather Brant (0000-0001-9608-7451), Helen Anderson (0000-0002-6945-0590), Helen Baxter (0000-0002-3320-2915), Karen Bloor (0000-0003-4852-9854), Sean Cowlishaw (0000-0002-8523-3713), Tim Doran (0000-0001-7857-3704), James Gaughan (0000-0002-8409-140X), Andrew Gibson (0000-0002-4641-2583), Nils Gutacker (0000-0002-2833-0621), Heather Leggett (0000-0001-8708-9842), Sarah Purdy (0000-0002-3445-986X), Sarah Voss (0000-0001-5044-5145), Jonathan Richard Benger (0000-0001-6131-0916).

York Trials Unit, Department of Health Sciences, ARRC Building, University of York, York, YO10 5DD, UK, Assistant Professor, Arabella Scantlebury

York Trials Unit, Department of Health Sciences, ARRC Building, University of York, York, YO10 5DD, UK, Professor, Joy Adamson

School of Social and Community Medicine, University of Bristol, Bristol, BS8 2PS, UK, Professor, Chris Salisbury

Faculty of Health and Applied Sciences, University of the West of England, Bristol, BS16 1QY UK, Senior Research Associate, Heather Brant

York Trials Unit, Department of Health Sciences, ARRC Building, University of York, York, YO10 5DD, UK, Research Fellow, Helen Anderson

School of Social and Community Medicine, University of Bristol, Bristol, BS8 2PS, UK, Research Fellow, Helen Baxter

Department of Health Sciences, ARRC Building, University of York, York, YO10 5DD, UK, Professor, Karen Bloor

Department of Psychiatry, University of Melbourne, Melbourne, VIC 3053, Australia, Senior Research Fellow, Sean Cowlishaw

Department of Health Sciences, ARRC Building, University of York, York, YO10 5DD, UK, Professor, Tim Doran

Department of Health Sciences, ARRC Building, University of York, York, YO10 5DD, UK, Research Fellow, James Gaughan

For peer review only

Faculty of Health and Applied Sciences, University of the West of England, Bristol, BS16 1QY, Associate Professor, Andrew Gibson

Department of Health Sciences, ARRC Building, University of York, York, YO10 5DD, UK, Senior Research Fellow, Nils Gutacker

York Trials Unit, Department of Health Sciences, ARRC Building, University of York, York, YO10 5DD, UK, Research Fellow, Heather Leggett

School of Social and Community Medicine, University of Bristol, Bristol, BS8 2PS, UK, Professor, Sarah Purdy

Faculty of Health and Applied Sciences, University of the West of England, Bristol, BS16 1QY, Professor, Sarah Voss

Faculty of Health and Applied Sciences, UWE Bristol, Glenside Campus, Blackberry Hill, Bristol, BS16 1DD, Professor, Jonathan Benger

**Correspondence to:** Jonathan Benger, Faculty of Health and Applied Sciences, University of the West of England, Bristol, BS16 1QY. Jonathan.Benger@uwe.ac.uk

For peer review only

For peer review only



For peer review only - http://bmjopen.bmj.com/site/about/guidelines.xhtml

# Abstract

**Objectives:** To examine the effect of General Practitioners (GPs) working in or alongside the Emergency Department (GPED) on patient outcomes and experience, and the associated impacts of implementation on the workforce.

**Design:** Mixed-methods study: interviews with service leaders and NHS managers; in-depth case studies (n=10) and retrospective observational analysis of routinely collected national data. We used Normalisation Process Theory to map our findings to the theory's four main constructs of coherence, cognitive participation, collective action and reflexive monitoring.

**Setting and participants:** Data was collected from 64 Emergency Departments (ED) in England. Case site data included: non-participant observation of 142 clinical encounters; 413 semi-structured interviews with policy makers, service leaders, clinical staff, patients and carers. Retrospective observational analysis used routinely collected Hospital Episode Statistics alongside information on GPED service hours from 40 hospitals for which complete data were available.

**Results:** There was disagreement at individual, stakeholder and organisational levels regarding the purpose and potential impact of GPED (coherence). Participants criticised policy development and implementation, and staff engagement was hindered by tensions between ED and GP staff (cognitive participation). Patient "streaming" processes, staffing and resource constraints influenced whether GPED became embedded in routine practice. Concerns that GPED may increase ED attendance influenced staff views. Our quantitative analysis showed no detectable impact on attendance (collective action). Stakeholders disagreed whether GPED was successful, due to variations in GPED model, site-specific patient mix and governance arrangements. Following statistical adjustment for multiple testing, we found no impact on: ED re-attendances within seven days, patients discharged within four hours of arrival, patients leaving the ED without being seen; inpatient admissions; non-urgent ED attendances and 30-day mortality (reflexive monitoring).

**Conclusions:** We found a high degree of variability between hospital sites, but no overall evidence that GPED increases the efficient operation of EDs or improves clinical outcomes, patient or staff experience.

**Trial registration:** ISCRTN5178022



For peer review only - http://bmjopen.bmj.com/site/about/guidelines.xhtml

## Strengths and limitations of this study

- National evaluation of the impact of general practitioners working in or alongside emergency departments in England.
- Mixed methods approach using a large qualitative data set (413 interviews, 142 non-participant observation) and routine national data sets involving multiple stakeholders across 64 emergency departments gave us a service wide and detailed understanding of the impact of GPED.
- Our data apply to England only and so may not be generalizable to other countries and healthcare settings.
- Our quantitative analysis was limited to routinely available data and so our analysis was dependent on key performance indicators and what is routinely collected and reported.

## Introduction

There were almost 24 million attendances at hospital emergency departments (EDs) in England in 2017-18, an increase of 22% since 2007/8.[1] This continues a long-term trend of increasing demand for urgent care at EDs that has also been observed in many other countries.[2] Workload pressures within these departments can lead to adverse effects on the quality of patient care, patient safety, clinical outcomes, patient satisfaction and staff job satisfaction.[3] One important measure of the performance of emergency departments in England is the target that 95% of patients should be admitted, transferred or discharged within four hours of arrival. This target has not been met nationally since 2015, with performance declining every year.[1]

About a fifth of patients attending emergency departments could be managed by general practitioners (GPs) in primary care settings, although estimates of this proportion vary widely depending on the definitions used.[4] Research suggests that the reasons patients choose to attend an emergency department with problems suitable for General Practice include: the perceived urgency of the situation, the belief that they need care only available in hospitals, the convenience of obtaining care at any time without an appointment, barriers to accessing general practice, and a lack of awareness of available primary care services.[5-7]

Several different policy initiatives have been proposed to address rising ED demand, and to allow EDs to focus on patients with the most urgent need.[8-10] These responses fall into three main categories: a triage step before patients attend EDs, such as a telephone advice line or "streaming" at the front door of the ED to direct patients to alternative services off-site;



For peer review only - http://bmjopen.bmj.com/site/about/guidelines.xhtml

better provision of alternative services (such as nurse-led walk-in services and Urgent Treatment Centres); improved access to GP services for people attending EDs. The latter approach can be achieved either by co-locating GP services alongside EDs at hospital sites, or by employing GPs to work within EDs to see selected patients. It has been suggested that GPs in or alongside the ED have the potential to improve patient care, and to reduce waiting times, unnecessary investigations, hospital admission rates, and costs,[11] but evidence to substantiate these claims is limited.[12-16] The introduction of these services was accelerated in 2017, when the UK government provided £100million of capital funding to support hospitals in England to provide a GP working in or alongside the ED,[17-19] as part of a comprehensive plan to reduce the growth of lower acuity patients attending EDs.[10] The aim of our research was to examine the effect of General Practitioners working in or alongside the Emergency Department (GPED) on patient outcomes and experience and the associated workforce and system impact. To incorporate all aspects of the research – both evaluative and the experiences associated with implementation – we have situated our work in the Normalisation Process Theory (NPT) framework.[20.21]

## Methods

### Design

We completed a mixed methods study including interviews with service leaders and NHS managers, in-depth case studies and a retrospective observational analysis of routinely collected national data. This approach enabled us to obtain a service-wide understanding of the impact of GPED on the urgent care system, the associated workforce and patient care.[22-25] Details of the study methodology have been published previously.[26]

Ethics committee approval was obtained from East Midlands – Leicester South Research Ethics Committee (ref: 17/EM/0312); University of Newcastle Ethics Committee (Ref: 14348/2016) and the Health Research Authority (IRAS: 230848 and 218038).

### Theoretical approach

We drew on NPT, which has been widely used to understand how and why things do or do not become embedded into routine practice.[20] Through its four core constructs of coherence, cognitive participation, collective action and reflexive monitoring (Box 1).[20,21] NPT can support both the understanding *and* evaluation of the implementation of organisational innovations such as GPED.[27] Its use has been supported by empirical studies using both qualitative and quantitative methods – therefore it was a particularly useful framework to apply in this context, given our study aims.[28,29]



For peer review only

**Box 1: The four core constructs of NPT, adapted for use in the GPED study**

| |
|---|
| **Coherence:** Do staff understand why GPED has been implemented? |
| **Cognitive participation:** Are staff engaged and committed to GPED, and what are the factors that promote and/or inhibit this commitment? |
| **Collective action:** Are participants using GPED and what are the factors that promote and/or inhibit them from using GPED? |
| **Reflexive monitoring:** Have staff appraised GPED and its impact on practice? |

NPT enabled us to integrate our qualitative and quantitative data; examining the extent to which GPED had become a part of routine practice and highlighting the related impact on patients and staff.

## Qualitative data collection and analysis

Qualitative data collection (Table 1) consisted of non-participant observation of 142 individual clinical encounters and 467 semi-structured interviews with key stakeholders (policymakers, service leaders, ED staff, General Practitioners, patients and carers). Qualitative data was distributed across 64 NHS EDs in England, 10 of which were in-depth case study sites. Data collection explored the impact of GPED from the perspectives of key stakeholders as well as the policy's background and factors affecting implementation. Following initial familiarisation and independent coding, the qualitative team, through a series of roundtable discussions and workshops with our patient collaborators, developed a coding framework (additional file 1). The coding framework, in conjunction with pen portraits of our ten case sites,[30] was used to facilitate cross-case comparisons and formed the basis of our main thematic analysis.[31] Initial analysis identified ten key themes (Table 2) – these included *Contested Policy*, which reflected stakeholder views on the concept of GPED and *Structural Implementation* relating to site level responses to the introduction of GPED. In addition, we identified eight themes which were factors our participants predicted would be affected by GPED (at time 1 qualitative data collection): *Performance against the four hour target; Use of investigations; Hospital admission; Patient outcome and experience; Service access; Staff recruitment and retention; Workforce behaviour and experience; Resource use*. We have collectively termed these eight themes as 'domains of influence',[32] which we have then used as outcome measures in our evaluation of GPED.



For peer review only - http://bmjopen.bmj.com/site/about/guidelines.xhtml

For peer review only



For peer review only - http://bmjopen.bmj.com/site/about/guidelines.xhtml

Table 1 Qualitative data collection

| | Policymakers | Service leaders | | Case sites | | |
|---|---|---|---|---|---|---|
| | Time 1 | Time 1 | Time 2 | Time 1 | Time 2 | Time 3 |
| Type of data collected | Semi-structured telephone interviews | Semi-structured telephone interviews | Semi-structured telephone interviews | Semi-structured face-to-face and telephone interviews, non-participant observations. | Semi-structured face-to-face and telephone interviews | Semi-structured face-to-face and telephone interviews, non-participant observations. |
| Aim of data collection | In-depth understanding of GPED policy and implementation from key informants | Broad perspective of GPED implementation and current provision from a range of EDs | Broad perspective of GPED implementation and current provision from a range of EDs | In-depth understanding from a small number of case sites | Brief 'check in' visits to assess any interim changes in GPED services | In-depth understanding from a small number of case sites |
| Period of data collection | December 2017 to January 2018 | August 2017-September 2018 | February 2018- February 2019 | November 2017-December 2018 | June-October 2018 | November 2018-December 2019 |
| Number of EDs | Not applicable | 64 | 30 | 10 | 5 | 10 |
| Stakeholder groups and organisations represented | NHS England and Improvement, Department of health, Clinical Commissioning Groups, NHS Trusts, Royal College of Emergency Medicine, GPs | Chief Executives, Chief Operating Officers, Clinical Leads, Lead nurses and ED managers | Chief Executives, Chief Operating Officers, Clinical Leads, Lead nurses and ED managers | GPs, ED doctors (juniors, registrars, consultants), Nurses (streaming, triage, emergency nurse practitioner), patients and carers | GPs, ED doctors (juniors, registrars, consultants), Nurses (streaming, triage, emergency nurse practitioner), patients and carers | GPs, ED doctors (juniors, registrars, consultants), Nurses (streaming, triage, emergency nurse practitioner), patients and carers |
| Total number of participants | 10 policymakers | 57 service leaders | 26 service leaders | 124 health professionals 94 patients/ carers<br>83 non-participant observations. | 20 health professionals | 82 health professionals, 54 patients/carers, 59 non-participant observations. |

*GPED: General Practitioners working in or alongside the Emergency Department; ED: Emergency Department; GP: General Practitioner.*



For peer review only - http://bmjopen.bmj.com/site/about/guidelines.xhtml

## Quantitative data and analysis

We completed a retrospective observational analysis of routinely collected Hospital Episode Statistics (HES) data between April 2018 and March 2019 from 40 English hospitals that were selected for their ability to provide complete data on the times of day when GPED services were available. Differences in GPED service availability between EDs at the same time of day were used to assign patients quasi-randomly to treatment or control groups at each hour of the day. Outcomes measured were: percentage of patients discharged within four hours of arrival; ED attendances that resulted in hospital admission; patients who left without being seen; unplanned re-attendance at the ED within 7 days; 30 day mortality; non-urgent ED attendances (described previously as ‘unnecessary’ and identified using a defined methodology);[33] volume of ED attendances. Each outcome was analysed separately using two-way fixed effects. Outcomes for patients attending different EDs at the same time of day were compared, exploiting variation in the timing of availability of GPED within the day at different EDs. Further details of this analysis have been published previously.[34] The potential net cost savings were explored using a comparative approach based on the results of this analysis.[35] We also conducted a survey of the GPED workforce at our 10 case sites, however, as these results did not materially alter our overall findings they are not reported here.[35]

## Mixed methods analysis

In addition to individual quantitative and qualitative analyses, we conducted higher-level synthesis to integrate the study findings using a triangulation protocol that combined different methods to gain a more complete picture.[36] Quantitative findings were grouped under the qualitative themes described above (Table 2). We then mapped our study findings onto the four core constructs of NPT (Tables 3-6).[20] Given the inter-related nature of the NPT constructs this process was undertaken by two researchers (JA and AS).

*Table 2 Qualitative and quantitative data integration*

| Theme | Qualitative | Quantitative |
|---|---|---|
| **Contested policy** | Qualitative interviews with policymakers and service leaders, health professionals, patients and carers. Non-participant observation | |



For peer review only - http://bmjopen.bmj.com/site/about/guidelines.xhtml

| | | |
|---|---|---|
| **Performance against the four-hour target** | Qualitative interviews with policymakers, health professionals, patients and carers. Non-participant observation | HES data: percentage of patients discharged within four hours of arrival |
| **Use of investigations** | Qualitative interviews with policymakers, health professionals, patients and carers. Non-participant observation | |
| **Hospital admissions** | Qualitative interviews with policymakers, health professionals, patients and carers. Non-participant observation | HES data: ED attendances that resulted in hospital admission |
| **Patient outcome and experience** | Qualitative interviews with policymakers, health professionals, patients and carers. Non-participant observation | HES data: patients who left without being seen<br>HES data: Unplanned re-attendance at the ED within 7 days<br>HES data: 30 day mortality |
| **Service access** | Qualitative interviews with policymakers, health professionals, patients and carers. Non-participant observation | HES data: non-urgent (described previously as 'unnecessary') ED attendances<br>HES data: Volume of attendances |
| **Staff (recruitment, retention)** | Qualitative interviews with policymakers, health professionals, patients and carers. Non-participant observation | |
| **Workforce (behaviour, experience)** | Qualitative interviews with policymakers, health professionals, patients and carers. Non-participant observation | |
| **Resource use** | Qualitative interviews with policymakers and service leaders, health professionals, patients and carers. Non-participant observation | |
| **Structural implementation** | Qualitative interviews with policymakers, health professionals, patients and carers. Non-participant observation | |

*ED: Emergency Department; HES: Hospital Episode Statistics.*

## Patient and public involvement

Patients and members of the public were involved throughout the development and delivery of this research. We formed a group of ten public contributors with a wide variety of



For peer review only - http://bmjopen.bmj.com/site/about/guidelines.xhtml

experiences of ED services. Throughout the study, the group were involved in regular workshops and meetings where they were asked to assist in interpreting the qualitative and quantitative data and support the development of our mixed methods synthesis. For instance, our lay contributors highlighted the central role played by the streaming nurse in GPED, which led to a further analysis of qualitative data surrounding streaming that has been published previously.[37] Two members of the group were also full members of the Study Steering Committee.

# Results

Tables 3-6 show how the themes from our qualitative and quantitative data map onto the four constructs of NPT.

### Coherence – do stakeholders have an understanding of why GPED was implemented?

For a health policy to be adopted into routine practice, there needs to be a shared sense of its purpose. Many stakeholders understood that GPED was being introduced as a direct response to rising pressures in EDs and as a potential mechanism for improving ED performance. Despite this, all stakeholder groups suggested that GPED was a rushed policy that lacked clear and consistent guidance. The fact that the policy was believed to originate largely from discussions between the Secretary of State for Health and NHS England, leading to "top down" implementation, and the lack of evidence supporting the clinical and cost-effectiveness of GPED were further causes of concern.

The decision to introduce GPED nationally was also based on the perceived success of a GPED service that had been implemented at a single NHS site – Luton and Dunstable (L&D). The rationale for choosing L&D as the national exemplar over other high-performing EDs was unclear, particularly given that it was difficult to determine whether the perceived success of L&D was due to GPED or the simultaneous introduction of other initiatives within the organisation. Associated with this were concerns that GPED failed to acknowledge local context and variations in demand for ED services, varying patient populations and pre-existing or prior attempts to implement GPED services.

This led to stakeholders questioning the generalisability of the national policy, and as a result GPED was interpreted differently with a range of models implemented throughout the NHS in England.[38,39]

There was widespread disagreement at an individual, stakeholder and organisational level about the purpose and potential impact of GPED. Despite disagreeing about the 'direction of effect,' stakeholders agreed on the areas of the healthcare system and patient care that GPED



For peer review only - http://bmjopen.bmj.com/site/about/guidelines.xhtml

was most likely to affect. We categorised these as eight themes as ‘domains of influence’ (Table 3),[32] which were subsequently used as the outcomes for our evaluation of GPED.

For peer review only



For peer review only - http://bmjopen.bmj.com/site/about/guidelines.xhtml

Table 3: Coherence - do stakeholders understand why GPED has been implemented?

| Questions | Themes | Illustrative data |
|---|---|---|
| Does GPED have a clear purpose and did participants have a shared sense of its this purpose?<br><br>Will GPED fit with the overall goals and activity of the organisation?<br><br>Is it clearly distinct from other interventions?<br>What benefits will the intervention bring and to whom? | ***Contested policy***<br>• The implementation of GPED was considered rushed, and to be based on conflicting guidance.<br>• Some stakeholders had difficulty understanding how GPED differed from other previously unsuccessful attempts to introduce GPs into the ED.<br>• It was uncertain how GPED, or the associated capital funding initiative, differed from previous and existing interventions.<br>• Variations in local context, ED demand and existing GP services in the ED resulted in GPED being interpreted and implemented differently.<br>***Domains of influence***<br>• GPED is difficult to describe, distinguish from other interventions and participants do not have a shared sense of its purpose.<br>• Stakeholders disagreed on the potential impacts of GPED, with positive, neutral or negative effects predicted for the majority of the eight identified domains of influence: 1) Performance against the four-hour target; 2) Use of investigations; 3) Hospital admissions; 4) Patient outcome and experience; 5) Service access; 6) Staff recruitment and retention, 7) Workforce behaviour and experience; 8) Resource use. | *"I think it adds to the mix. I think that it was not a very well thought through policy decision … It was never part of the urgent, the care, the Keogh review of urgent emergency care to have GPs in ED. Now that review focused much more on NHS 111 and also trying to create consistency … So having GPs in ED, was outside of that policy strand. So, and it was dropped in a very, at very great speed and without a great deal of thought." (Interview with service leader)*<br><br>*"You know, it isn't a sufficient evidence base to work from. You could have looked at the North East of England, I'm taking this call just now and said, you know, six of the top ten performers nationally sit in the North East, alright, and that tells us something about the system... and I think that, if we're going to use examples as a way of developing policy, that would have been a better way of looking at it." (Interview with Policymaker)*<br><br>*"Whilst we started with a very clear - here's the Luton model, it became, obviously when trusts came to implement it locally that due to various circumstances that were very specific to their trust and their community, the Luton model just wasn't appropriate. So, I think what we've ended up with is a range of different models. So, you couldn't look at GP streaming and say what we've got in place now is the same in every trust in the country because there's almost certainly ... there's huge variation in practice around how they're running." (Interview with policymaker)* |



For peer review only - http://bmjopen.bmj.com/site/about/guidelines.xhtml

Table 4: Cognitive participation - are people committed to using GPED and what are the factors that promote and/or inhibit this commitment?

| Questions | Themes | Illustrative data |
|---|---|---|
| Did stakeholders see the point easily?<br><br>Were stakeholders prepared to invest time, energy and work in GPED? | ***Contested policy***<br>• There was doubt whether GPED, as a single initiative, could fix complex problems in the healthcare system.<br>• GPED policy development was criticised, as was the fact that it was based on limited evidence and patient and clinical consultation. This reduced stakeholders' commitment to ensuring it was embedded into routine practice. | *"Because it [GPED] is cheaper than re-investing in social care. Preventing inappropriate admissions is right, but it doesn't solve all the problems in primary care - those patients that do need to be seen and do need support in the community/social care, [GPED] is not a long-term solution." (Interview with service leader)*<br><br>*"It [streaming criteria] should be fixed, but, as I said, depending on who you speak to, it does waver slightly on what practitioners and GPs are willing to see. So, it's a bit of a grey area really. It depends who you're working with really. I don't . . . yes. So, it's not fixed. It should be really." (Interview with Advanced Nurse Practitioner at Case site Redwood).*<br><br>*"Actually, looking at X-rays and ECGs is, it becomes a bit of a, a dying art in General Practice, if you're not looking at those sorts of things on a daily basis, and what we provide again is allowing GPs the ability to keep those sort of clinical skills up and running, when I think that, and I think that's the attractiveness about doing this." (Interview with Urgent Care Centre clinical lead at case site Teak)* |



For peer review only - http://bmjopen.bmj.com/site/about/guidelines.xhtml

Table 5: Collective action - are people using GPED and what are the factors that promote and/or inhibit them from using GPED?

| Questions | Themes | Illustrative data |
|---|---|---|
| What effect will GPED have on the ED and health service?<br>How will the intervention affect the work of patients and staff?<br><br>Will staff require further training?<br><br>What impact will it have on division of labour, resources, power and responsibility between different professional groups?<br><br>What are the factors that promote and/or inhibit them from using GPED? | ***Service Access***<br>• Despite reports that GPs have been working in the ED for some time, only a small number of patients reported using GPED previously and expected to be streamed to GPED.<br>• Staff were concerned that GPED may create 'easy access to a GP', encouraging people to attend.<br>• Staff were concerned that patients attended the ED "inappropriately", and considered poor health literacy to affect how patients use GPED.<br>• GPED and 'Urgent Care' were considered confusing to patients and made navigating services more challenging.<br>• Analysis of HES data identified no significant impact on: volume of ED attendances; number of non-urgent (described previously as 'unnecessary') attendances<br>***Staff recruitment and retention***<br>• Staffing issues posed a major threat to the successful implementation and adoption of GPED.<br>• Nursing shortages and a lack of experienced nurses made the staffing of streaming services challenging.<br>• Streaming may change the role of nurses and divert them away from core ED work, making GPED settings less attractive. The psychological and physical impact of streaming may negatively affect nurses' work and willingness to invest energy and time in GPED.<br>• GPED may draw GPs away from traditional General Practice. ED staff vacancies created issues in the recruitment of ED and GP staff.<br>• To overcome recruitment issues, GPED needs to be viewed as an attractive place to work.<br>The training and educational benefits that junior doctors may receive from working alongside GPED models were considered valuable, and may make them more committed to ensuring GPED is embedded into routine practice.<br>***Use of investigations***<br>• There was a lack of consensus as to whether GPED models should give GPs access to diagnostic testing, reflecting differing interpretations of the purpose of GPED and varying local needs. This | *"what appeals to me is that I can do a bit of acute general medicine, trauma etc. and I'm trained in that but equally, I can also lapse into what was my comfort zone … and that works really well whereas when I'm feeling a bit more sort of "right, come on, you know, I can get into resus and I can learn a new thing' and I really enjoy that." (Interview with GP at case site Juniper).*<br><br>*"The GP feels that one of the problems with the model, is that there is a need for experienced triage nurses in order for it to work, but the department has a high turnover of nursing staff and has difficulty retaining staff. There are only a couple of appointed nurses who have the experience required." (Interview with ED Consultant at case site Redwood).*<br><br>*"ED's frightened to send anything away, so everything comes in. So, I don't blame the public for attending if they can see a GP within three hours, rather than having to wait six, seven days or two weeks for an appointment. But I just wonder if it's made a demand for it, because you get people coming back to see the GP again in ED. (Interview with Nurse at case site Rowan)*<br><br>*"Patients are savvy as well, tell you what they think they want you to hear in order to get them into the service they want to be seen by." (Interview with Nurse at case site Linden).*<br><br>*"I think it's down to, obviously, your training, but also how risk averse you are, and some people are very risk averse and will just have a much lower* |



For peer review only - http://bmjopen.bmj.com/site/about/guidelines.xhtml

| | | |
|---|---|---|
| | caused tension between GP and ED staff and may make staff less likely to invest their time and energy into GPED.<br>***Workforce behaviour and experience***<br>• Good communication, trust and confidence between streaming staff and GPs are pivotal to the effectiveness of GPED.<br>• Staff were concerned about patients who attend the ED with conditions that could be treated in general practice, but had different perceptions of what constitutes a 'GPED appropriate patient.'<br>• Tensions between GPs and staff responsible for streaming decisions were common and reflected different attitudes to risk as well as staff members (ED and GP) protecting their own working environment – staff streamed patients to GPED, or back to ED during busy periods, to ease their respective workloads.<br>• Streaming protocols were developed to try to standardise streaming decisions and GPED acceptance criteria, however these were not consistently disseminated or followed.<br>***Structural implementation***<br>• Several implementation issues also affected the extent to which staff were able to embed GPED into their routine practice including structural support within the site, ensuring integrated information technology systems between ED and GPED and influencing factors relating to the GP's role such as ensuring a positive working environment and giving GPs access to investigations, where appropriate. | *threshold for streaming people into ED and then also the Urgent Care Centre, rather than directing appropriately, you know, taking that risk." (Interview with Paramedic at case site Chestnut).*<br><br>*"It's going on long enough to do and we really just didn't know what else to do. I literally can't drive. I'm having trouble getting out of the house. We could do it today and get here and try and figure out what was going on, rather than go to the GP, the GP say, "Do this, then come back," then almost probably end up in the hospital as it's going there anyway, to do the same things. That was the decision really." (Interview with patient at case site Hawthorn).* |

Table 6: Reflexive monitoring - have people appraised GPED and its impact on practice?

| Questions | Themes | Illustrative data |
|---|---|---|
| Will it be clear what effects the intervention has had?<br><br>How are users likely to perceive the intervention | ***Performance against the 4 hour target and hospital admissions***<br>• There was no significant impact on the proportion of patients meeting the four hour target, or on the number of attendances resulting in a hospital admission.<br>• Variations in site-specific patient mix, GPED models and whether patients streamed to GPED were included in ED reporting statistics, | *"Yeah, I think that's really important, I think given the way the hospital performs with the Government's four-hour target, I think it's a source of pride for the hospital for the Chief Exec." (Interview with ED Consultant at case site Linden).* |



For peer review only - http://bmjopen.bmj.com/site/about/guidelines.xhtml

| | | |
|---|---|---|
| once it has been in use for a while?<br><br>Is it likely to be perceived as advantageous for patients or staff? | combined with other factors that influence ED performance, may have contributed to the apparently limited effects of GPED.<br>***Resource use***<br>• Any possible cost savings due to reduced reattendances were much smaller than the cost of providing the service itself.<br>***Patient outcome and experience***<br>• Most patients saw the value of GPs working in or alongside the ED as long as they received appropriate care.<br>• Staff felt that GPED may negatively affect patient flow.<br>There was no significant impact on the following performance indicators in the HES analysis: left without being seen; 30-day mortality; re-attendance to the same ED within seven days. | *"I don't necessarily think it is a bad thing to have it, but it provides marginal gains, and those marginal gains are, happening at a very high capital cost and an ongoing staffing cost and looking at the NHS budget as a whole, I think it's a shocking waste of money." (Interview with ED consultant at case site Juniper)* |



For peer review only - http://bmjopen.bmj.com/site/about/guidelines.xhtml

## Cognitive participation – are people committed to using GPED and what are the factors that promote and/or inhibit this commitment?

The way in which GPED policy was designed and implemented, along with challenges in translating a national policy to meet local service and population needs, caused some to view GPED as a 'sticking plaster solution' to ED pressures. For many, the rise in ED attendances was driven by wider, more complex issues across health and social care, which were often deemed to be the result of deficiencies elsewhere in the system. As a result, there was doubt that a single initiative such as GPED could provide the solution. This lack of buy-in from stakeholders was reflected during interviews with service leaders and policymakers where alternative solutions for improving ED performance were proposed. For example, investment in social care and mental health services were considered to have a greater potential for impact.

Embedding GPED into existing practice requires commitment from key stakeholders. Emphasis was placed on the importance of streaming nurses and GPs working together to stream patients from ED to GPED. Despite many sites trying to ensure consistency through the development of streaming protocols, the challenges of disseminating and adhering to these protocols, reliance on locum and/or part-time GPs and frequent rotation of streaming nurses meant that the definition of a patient suitable for GPED varied between and within professional groups. This, combined with the cultural differences in how GPs and ED clinicians work, and their inherently different approaches to risk, was a source of tension that in some cases resulted in patients not being accepted by GPED and sent back to ED.

Whether GPED models gave GPs access to investigations such as x-rays and blood tests varied across case sites and reflected the different interpretations of the purpose of GPED and varying local contexts. Some individuals considered giving GPs access to investigations and diagnostic tests as crucial to the model's effectiveness by supporting GPs to treat a broader range of patients and refer to inpatient specialties. However, others felt that doing so asked GPs to work beyond their clinical competency – some staff felt that there was a shortage of GPs with the skills required to interpret some ED diagnostic tests, and an upskilling of the GP workforce would therefore be required. As a result, some GPs were asked to work as they would in general practice, whilst other services preferred those with prior ED experience.

## Collective action – are people using GPED and what are the factors that promote and/or inhibit them from using GPED?

At the time that GPED was introduced, general practice in England was facing a significant workforce crisis. This posed a real challenge both in terms of ensuring that EDs were able to recruit GPs to work in GPED and ensuring that in doing so workforce shortages elsewhere in



For peer review only - http://bmjopen.bmj.com/site/about/guidelines.xhtml

the system were not exacerbated. Site staff suggested that to facilitate the recruitment of GPs, emphasis should be placed on ensuring that GPED was considered an attractive place to work and on supporting GPs to work within the scope of their practice. However, whether GPED was viewed as a positive role depended on the individual GP. For instance, whilst GPED may be appealing to those who wish to expand their work beyond traditional general practice, the scope, acuity and shift-based working that are typical of the ED may contradict why many individuals chose to become a GP in the first place.

Ensuring that streaming is undertaken by experienced streaming nurses was also considered pivotal to an effective GPED service. However, nursing shortages, the psychological and physical burden of streaming on nurses and the potential for streaming to divert nurses away from their routine ED work meant that recruiting nurses to streaming roles was challenging.[37]

Our findings also identified several other factors that may promote or inhibit how staff use GPED, and the extent that it becomes embedded into routine practice (Table 7). These were categorised as those relating to; workforce behaviour and experience (communication, trust and role-based cultural differences) and streaming and implementation issues (streaming protocols, inter-professional relationships and structural support).

Service leaders and site staff were concerned that giving patients ‘easy access’ to a GP, in a climate where general practice appointments may be difficult to obtain, could encourage patients to attend the ED rather than their own GP. Staff were particularly critical of patients for what they considered ‘inappropriate ED attendance’ (i.e. attending the ED when they perceived alternative services would meet their needs). Whilst this was largely attributed to the potentially confusing range of services available, re-organisation and re-branding of existing services and perceived low levels of health literacy making service navigation difficult for patients, there were also some patients who were accused of deliberately ‘playing the system’. For example, some patients were thought to deliberately bypass their GP and attend ED to access investigations, referrals or treatments. However, the reasons that patients chose to attend ED were complex, and in some cases, those that were considered by staff to have attended “inappropriately” had been advised to attend the ED by other healthcare professionals and services such as NHS111, a pharmacy or their own GP.

However, our qualitative data provided numerous examples of situations in which experienced nurses were unable to determine whether a patient’s complaint should be treated by general practice or the ED, suggesting that it may be unreasonable to expect patients to make the ‘correct’ choice on every occasion.

Despite these concerns amongst site staff, analysis of HES data found no association between non-urgent attendances and GPED or the absolute and relative volume of



For peer review only - http://bmjopen.bmj.com/site/about/guidelines.xhtml

attendances and GPED.[34] Despite staff believing that GPED may encourage ED use, the qualitative data highlighted that patients attend the ED for a variety of reasons, and demonstrate reasoned decision-making in their service use. Only a small number of patients expected to see a GP, with the majority showing no awareness of GPED when interviewed. This is perhaps unsurprising given that sites often chose not to advertise GPED services to reduce the likelihood of driving an increase in ED attendances.

## Reflexive monitoring – have people appraised GPED and its impact on practice?

GPED is a complex intervention that has been introduced through a range of different models, into a complex and changing environment. EDs serve different patient populations and have different physical structures, staff mixes and care provision. In addition to this heterogeneity, the widespread uncertainty surrounding GPED operating hours and different governance arrangements across sites meant that there was variation in whether patients streamed to GPED were counted in nationally reported ED statistics. The challenges of using key performance indicators to evaluate national policies such as GPED was discussed by service leaders, who questioned their utility and described indicators such as the target that 95% of patients attending the ED should be admitted, transferred or discharged within four hours as ‘blunt tools’ for evaluating impact.

Our quantitative analysis showed no statistically significant improvement in a range of key performance indicators across several domains of influence including the “four-hour target”, hospital admissions and patient outcomes and experience (patients leaving the ED without being seen and mortality at 30 days after an ED attendance). We did observe that GPED reduced the probability of unplanned re-attendance within seven days by 3.2% (OR: 0.968, 95% CI: 0.95 to 0.99), which equates to approximately 300 fewer re-attendances per year for an average ED in England. After adjustment for multiple testing, however, this difference was no longer statistically significant, and was also not judged to be clinically significant. Possible cost savings associated with reduced reattendances (£30-37,000 per ED per year) were heavily outweighed by the cost of GPED services. In the hospitals for which we had data, the average length of time of operation of a GPED service was 11.1 hours per day. Assuming only one GP is present and including salary costs of the GP alone (potentially a substantial underestimate), this amounts to around £454,000 per ED per year. As a result, current GPED models do not appear to be an efficient use of healthcare resources.[35]



For peer review only - http://bmjopen.bmj.com/site/about/guidelines.xhtml

The majority of patients we interviewed valued GPED and considered it beneficial to have GPs in EDs. Patients were aware that GPED may relieve pressure on the ED, ensuring emergency doctors can deal with the "real emergency cases" and were indifferent to the type of health professional that they saw as long as they received appropriate care. Similarly, the "four-hour target" was not a priority for patients, with many explaining that they were happy to wait longer as they understood that they were guaranteed to be seen and were waiting because priority was given to higher acuity patients. Despite this, staff raised concerns that GPED could negatively impact patient flow, as patients are required to disclose clinical information on multiple occasions before seeing a GP, which may create a backlog.

## Discussion

The GPED study was commissioned to evaluate the impact of GPs working in or alongside EDs; a national policy implemented in response to rising pressures on EDs in England. GPED had no effect on a range of routinely collected ED performance measures. Despite considerable concern from health professionals that GPED may actually increase demand, we found no significant effect of GPED on ED attendances or reattendances within 7 days. This was supported by our qualitative analysis; most of the patients that we interviewed were unaware of GPED and had not changed their behaviour as a result. We observed confusion amongst patients, staff, service leaders and participating NHS organisations as to the purpose of GPED, with a prevailing view that the main drivers of ED workload may be more related to an ageing population, high inpatient bed occupancy and a shortage of social care[40] than attendances by patients suitable for management in traditional general practice.

Early evaluations of GPED models of care in the UK and internationally suggested that placing GPs in the ED was a promising innovation.[41] Studies reported that GPED had the potential to reduce resource use,[42,43] and increase patient satisfaction.[44] Carson et al.[45] found that the proportion of cases seen by GPs varied and that clinical and operational governance was often disjointed. In a survey of patients, Bickerton et al.[46] found that whilst GPED offered patients a greater range of service provision, it also increased the risk of duplication and repeat attendance. More recently, in a relatively small study, Uthman et al. found that GPs who saw patients in the ED used fewer resources without increasing reattendance, and referred more patients to follow-up services.[47] In addition, service users appreciated simplified health-care provision from a single point of access.[48]

It is not 22ncommonn for early reports of new initiatives to be positive, but contradicted subsequently,[49] and our study is the largest of GPED services published to date. A similar phenomenon was observed previously in relation to nurse-led walk-in centres co-located with



For peer review only - http://bmjopen.bmj.com/site/about/guidelines.xhtml

the ED, whereby initially positive reports were challenged by a subsequent large-scale evaluation that found 'no evidence of any effect on attendance rates, process, costs or outcome of care'.[50] Furthermore, our data demonstrate considerable heterogeneity, with the implication that whilst our overall result is null, GPED may still have beneficial effects in some locations and under certain circumstances. Our findings suggest that GPED implementation is highly sensitive to local context, and these contexts will govern the success of any particular scheme. This is consistent with other evaluations of urgent and emergency care initiatives,[51] Investment in GPED appears justified only when the factors associated with success are in place (see below), and there is clear evidence of benefit at a local level. Where this evidence of local benefit is absent alternatives to GPED should be considered, such as improving provision and access in traditional General Practice, both in and out of hours.

Our quantitative analysis used routinely available data, and it would be surprising if some of these measures (e.g. 30 day mortality) were influenced by GPED. It has also been noted that patients eligible for GPED are often quick and easy to manage, do not breach the '4-hour target', are less likely to be admitted and do not contribute to crowding.[45,52] A recent realist review concluded that, despite GPs in ED being associated with a reduction in process time for non-urgent patients, this does not necessarily increase capacity to care for the sickest patients.[12] The main cause of ED crowding is perceived to be congestion in the flow of sicker patients into the hospital and a lack of beds, rather than absolute attendance numbers.[53]

The GPED study shows that even when a policy is mandatory and supported by dedicated capital funding, this does not guarantee successful or uniform adoption. Our findings highlight the complexities of translating policy into practice, and the importance of considering the extent that a government-led policy can be delivered at a local level. Previous evidence suggests that a common response to national policy is local adaptation, which can in turn lead to the implementation of different innovations to those that are originally proposed.[22] We found evidence of this, as interviewees often described a range of approaches to GPED that sometimes opposed the high-level policy messages that accompanied the provision of capital funding. It also remains uncertain whether revenue funding, as well as (or instead of) capital funding would have alleviated some of the noted challenges.

Our qualitative data also identified a range of factors that can facilitate implementation. We present these as a series of 'success factors' which may inform how services choose to implement future GPED models; or adapt existing ones (Table 7). At several of our case study sites, these fundamentals had been overlooked and the result was a less coherent GPED



For peer review only - http://bmjopen.bmj.com/site/about/guidelines.xhtml

service. However, it is important to note that even if all these ‘success factors’ are implemented, our findings do not present evidence that the resulting GPED service would have a positive impact on ED performance indicators or be cost-effective.

*Table 7 Success factors for the implementation of GPED.*

For peer review only



For peer review only - http://bmjopen.bmj.com/site/about/guidelines.xhtml

| Success factor | How can this be addressed? |
|---|---|
| **Streaming** | No single model for effective streaming was identified. The factors listed below should be considered when developing future streaming models. |
| *The experience and seniority of streaming nurses* | Effective streaming requires high levels of clinical knowledge, critical thinking, clinical decision-making and balancing clinical risks. Streaming should be undertaken by senior nurses. |
| *The skills, confidence and abilities of GPs* | Professional groups had different opinions as to what can be considered a "GP appropriate" patient. To alleviate tension between staff there needs to be a shared understanding of streaming protocols and an awareness of the skills and scope of practice of GPs. Recruiting experienced and clinically knowledgeable GPs who are willing to adapt and see a broader range of patients is helpful. |
| *Inter-professional relationships* | Trust and confidence between professional groups is essential. Co-location does not automatically ensure collaboration. Individuals naturally work within professional norms. Effective communication and common goals mitigate tension. |
| *Streaming protocols* | Stakeholder clinicians (including streamers and GPs) should be involved in the development and regular review of protocols. These should be effectively communicated to all relevant practitioners. For streaming to be effective, streamers may need to deviate from protocols based on their clinical judgment. Staff should be supported to do this, while also considering strategies to mitigate against inappropriate deviation which may negatively impact patient care. |
| *Streaming safety* | Safety concerns limit the effectiveness of streaming strategies and sources of support are needed to ensure staff feel confident in their decision making.<br>Clinicians should be involved in the development and regular review of protocols. These include effective pathways for managing deteriorating patients and returning streamed patients back to the ED when necessary. Consider ways to make the streaming process clearer for patients to navigate, to reduce repetition and patient frustration. Onward referrals were often a pinch point in the system, with patients at risk of increased waiting times or being overlooked. Guidance and support for streaming |



For peer review only - http://bmjopen.bmj.com/site/about/guidelines.xhtml

| | |
|---|---|
| | nurses experiencing complaints processes, litigation or professional registration issues should be provided. |
| **Staffing** | Less reliance on locum GPs and ensuring GPED shifts are covered consistently, and communicated effectively, promotes consistency. Recruitment of highly experienced and clinically knowledgeable GPs who are willing to adapt their practice to take on a broader range of work<br><br>Consider retention strategies to support current streaming nurses and to futureproof streaming by training and retaining adequate numbers of suitably experienced nurses. Streamers should be supported by their professional colleagues. Implement strategies to mitigate against burnout, prevent overload from additional responsibilities and positive promotion of streaming roles to make them attractive to nurses. |
| **Leadership** | Involve staff of all grades and from all key professional groups in the development and implementation of service planning, organisation and protocol development to counteract feelings of top-down change and encourage buy-in and support. |
| **Physical environment** | Consider the impact of the physical environment, e.g. privacy at the streaming desk, safety of both staff and patients in isolated or exposed streaming areas, and for GPs located away from the ED and in off-site Hubs. Inadequate space can lead to overcrowding. Patients who have to queue more than can become confused and frustrated. Consider where GPs are placed to avoid feeling isolated and separated from the ED. |
| **Integrated IT systems** | Effective, easy to use and joined up information technology systems between ED, GPED and General Practice are essential for a safe working environment. |
| **Structural support** | Support for streamers should include specific training, regular supervision, audit and feedback. GPED models and streaming services should be planned and organised with involvement and buy-in from key stakeholders including streaming nurses and GPs. |

*GPED: General Practitioners working in or alongside the Emergency Department; ED: Emergency Department; GP: General Practitioner.*



For peer review only - http://bmjopen.bmj.com/site/about/guidelines.xhtml

GPED is a new policy initiative, which has been evaluated by two large NIHR commissioned research studies (HS&DR Projects 15/145/04 and 15/145/06).[26,35,39,54] Further research evaluating its impact is therefore not recommended until the policy has been given time to embed into routine practice. Instead, priority should be given to evaluating existing performance measures and developing new, rapid methods to inform the development, implementation and evaluation of similar health policy initiatives (Box 2).

**Box 2: Implications for future research**

1. The utility and completeness of national routine data sets limit the ability to evaluate the impact of complex health initiatives across a range of outcomes. Patients and clinicians should be consulted to ensure that measures of 'success' include outcomes that are important to all stakeholder groups and how these can be captured.
2. The relationship and interface between general practice and secondary care is crucial to the future delivery of urgent and emergency care. Research to explore this relationship and different approaches to risk will inform future models of service development and delivery in the context of rising healthcare demand.
3. We identified particular ambiguity and uncertainty in relation to streaming in the ED. Further research to clarify the optimal approach to streaming in terms of patient outcome, safety and experience, and the wider implications of streaming on staff experience, is warranted.

## Strengths and limitations

We adopted a mixed-methods approach which consisted of 'big qualitative' data collection (413 interviews and 142 individual observations of clinical encounters) and quantitative analysis of national data sets to explore the impact of GPED. This approach, and the decision to interpret our study findings using NPT, provided us with an in-depth understanding of the impact of GPED. This highlighted the complex interplay of political, workforce and social factors that affect successful adoption of a health policy into routine practice.

Our data apply to England only, and so may not be generalizable to other countries and healthcare settings. In our quantitative analysis, it was not possible to identify from available data which staff members assessed and treated individual patients, so we could not separate patients treated by GPs from those treated by other ED staff to directly compare GP services



For peer review only - http://bmjopen.bmj.com/site/about/guidelines.xhtml

to traditional models of care. We relied primarily on measures of general ED performance, such as attendances, patient flow and waiting times. We were also limited in our ability to collect data from the general practice and urgent care systems surrounding our case study sites, which significantly limited our ability to evaluate quantitatively the effect of GPED on the wider healthcare system. Our qualitative case study sites were selected purposively to be as representative as possible. However, participation by sites, and from staff and patients during data collection, was voluntary and so is unlikely to be exhaustive.

## Conclusion

Implementation of General Practitioners working in or alongside the ED was highly subject to local context and micro-level influences. However, we found no consistent evidence of improvements in patient outcome or experience. This is summed up by our public contributors, who following presentation of the final study findings concluded:

*"GPED is not effective and should only be used where specific circumstances indicate that it may play a positive role."*

**Contributor and guarantor information**

JB, JA, HBa, KB, SC, TD, AG, NG, SP, CS, SV had the initial research idea and obtained funding for this study. Qualitative data collection and analysis were undertaken by HA, JA, HL, AS. Quantitative data collection and analysis were undertaken by KB, TD, JG, NG, HBr, SC. Mixed methods analysis was undertaken by JA and AS. AS and JA drafted the paper and are joint first authors for the manuscript. JB and CS assisted in drafting the manuscript. AS, JA, CS, HBr, HA, HBa, KB, SC, TD, JG, AG, NG, HL, SP, SV, JB critically reviewed, revised and approved the final manuscript. JB is the study guarantor. The guarantor (JB) accepts full responsibility for the work and the conduct of the study, had access to the data, and controlled the decision to publish. The corresponding author attests that all listed authors meet the authorship criteria and that no others meeting the criteria have been omitted.

**Copyright/license for publication**

"The Corresponding Author has the right to grant on behalf of all authors and does grant on behalf of all authors, a worldwide licence to the Publishers and its licensees in perpetuity, in all forms, formats and media (whether known now or created in the future), to i) publish, reproduce, distribute, display and store the Contribution, ii) translate the Contribution into other



For peer review only - http://bmjopen.bmj.com/site/about/guidelines.xhtml

languages, create adaptations, reprints, include within collections and create summaries, extracts and/or, abstracts of the Contribution, iii) create any other derivative work(s) based on the Contribution, iv) to exploit all subsidiary rights in the Contribution, v) the inclusion of electronic links from the Contribution to third party material where-ever it may be located; and, vi) licence any third party to do any or all of the above.

**Patient consent for publication**

Not applicable

**Data availability statement**

The deidentified patient-level data used for the quantitative component of this study, including information on mortality, were released by the data holders (NHS Digital, Office for National Statistics) under specific data sharing agreements and only for the purpose of this study. The data sharing agreements do not permit further sharing or publication of the data. Interested parties may seek to obtain data directly from the relevant data holders. Hospital Episode Statistics (HES) data are copyright 2018-2019, reused with the permission of NHS Digital through Data Sharing Agreement NIC-84254-J2G1Q. The data about the hours a general practitioner services was operating in emergency departments was collected by the authors specifically for this project. The authors are not able to place the original data into the public domain. The qualitative data we have acquired will not be available as our ethical approval does not permit the sharing of the entire data set.

**Ethical approval**

Ethical approval was obtained from East Midlands – Leicester South Research Ethics Committee (ref: 17/EM/0312); University of Newcastle Ethics Committee (Ref: 14348/2016) and the Health Research Authority (IRAS: 230848 and 218038). All participants provided informed consent before taking part in the qualitative study.

**Transparency declaration**

The lead authors (AS, JA) and manuscript guarantor (JB) affirm that the manuscript is an honest, accurate and transparent account of the study being reported; that no important aspects of the study have been omitted; and that any discrepancies from the study as originally planned (and, if relevant, registered) have been explained.

**Funder**



For peer review only - http://bmjopen.bmj.com/site/about/guidelines.xhtml

Department of Health. National Institute for Health Research. Health Services and Delivery Research Programme. Grant number 15/145/06.

**Competing interests**

All authors have completed the ICMJE uniform disclosure form and declare: support from the National Institute for Health Research (NIHR) Health Services and Delivery Programme for the submitted work*; no financial relationships with any organisations that might have an interest in the submitted work in the previous three years; no other relationships or activities that could appear to have influenced the submitted work."*

Acknowledgements

The authors would like to thank the participants for their involvement in the qualitative study, and the patient and public contributors.

## References:

1. NHS Digital. Hospital Accident and Emergency Activity 2017-2018 Online: NHS Digital; 2018 [Available from: https://digital.nhs.uk/data-and-information/publications/statistical/hospital-accident--emergency-activity/2017-18 accessed 23/11/2021 2021.

2. Berchet C. Emergency Care Services. 2015 doi: doi:https://doi.org/10.1787/5jrts344crns-en

3. Morley C, Unwin M, Peterson GM, et al. Emergency department crowding: a systematic review of causes, consequences and solutions. *PloS one* 2018;13(8):e0203316.

4. Morris T, Mason SM, Moulton C, et al. Calculating the proportion of avoidable attendances at UK emergency departments: analysis of the Royal College of Emergency Medicine's Sentinel Site Survey data. *Emergency Medicine Journal* 2018;35(2):114-19. doi: 10.1136/emermed-2017-206846

5. Vogel JA, Rising KL, Jones J, et al. Reasons Patients Choose the Emergency Department over Primary Care: a Qualitative Metasynthesis. *Journal of General Internal Medicine* 2019;34(11):2610-19. doi: 10.1007/s11606-019-05128-x



For peer review only - http://bmjopen.bmj.com/site/about/guidelines.xhtml

6. Coster JE, Turner JK, Bradbury D, et al. Why Do People Choose Emergency and Urgent Care Services? A Rapid Review Utilizing a Systematic Literature Search and Narrative Synthesis. *Academic Emergency Medicine* 2017;24(9):1137-49. doi: 10.1111/acem.13220

7. O'Cathain A, Connell J, Long J, et al. 'Clinically unnecessary' use of emergency and urgent care: A realist review of patients' decision making. *Health Expectations* 2020;23(1):19-40. doi: 10.1111/hex.12995

8. College of Emergency Medicine. Acute and emergency care: prescribing the remedy. London: College of Emergency Medicine, 2014.

9. Urgent and Emergency Care Review Team, Emergency Care Intensive Support Team. Transforming urgent and emergency care services in England. Safer, faster, better: good practice in delivering urgent and emergency care. A guide for local health and social care communities. London: NHS England, 2015.

10. NHS England. Next Steps on the NHS Five Year Forward View. England, 2017.

11. Boeke AJP, van Randwijck-Jacobze ME, de Lange-Klerk EM, et al. Effectiveness of GPs in accident and emergency departments. *British Journal of General Practice* 2010;60(579):e378-e84. doi: 10.3399/bjgp10X532369

12. Cooper A, Davies F, Edwards M, et al. The impact of general practitioners working in or alongside emergency departments: a rapid realist review. *BMJ Open* 2019;9(4):e024501. doi: 10.1136/bmjopen-2018-024501

13. Ramlakhan S, Mason S, O'Keeffe C, et al. Primary care services located with EDs: a review of effectiveness. *Emergency Medicine Journal* 2016;33(7):495-503. doi: 10.1136/emermed-2015-204900

14. Turner J, Coster J, Chambers D, et al. What evidence is there on the effectiveness of different models of delivering urgent care? A rapid review: NIHR Journals Library, Southampton (UK) 2015.

15. Gonçalves-Bradley D, Khangura JK, Flodgren G, et al. Primary care professionals providing non-urgent care in hospital emergency departments. *Cochrane Database of Systematic Reviews* 2018(2) doi: 10.1002/14651858.CD002097.pub4

16. National Institute for Health and Care Excellence. GPs within or on the same site as emergency departments, 2017.

17. Iacobucci G. All emergency departments must have GP led triage by October: British Medical Journal Publishing Group, 2017.



For peer review only - http://bmjopen.bmj.com/site/about/guidelines.xhtml

18. HM Treasury. Spring Budget. London, 2017.

19. Department of Health and Social Care. Funds for next winter will ease pressure on accident and emergency (A&E) departments in England. London, 2017.

20. May CR, Mair F, Finch T, et al. Development of a theory of implementation and integration: Normalization Process Theory. *Implementation Science* 2009;4(1):1-9.

21. May CR, Johnson M, Finch T. Implementation, context and complexity. *Implementation Science* 2016;11(1):1-12.

22. Pope C, Robert G, Bate P, et al. Lost in translation: a multi-level case study of the metamorphosis of meanings and action in public sector organizational innovation. *Public Administration* 2006;84(1):59-79.

23. Travaglia JF, Nugus P, Greenfield D, et al. Contested innovation: the diffusion of interprofessionalism across a health system. *International Journal for Quality in Health Care* 2011;23(6):629-36.

24. Caldwell SE, Mays N. Studying policy implementation using a macro, meso and micro frame analysis: the case of the Collaboration for Leadership in Applied Health Research & Care (CLAHRC) programme nationally and in North West London. *Health research policy and systems* 2012;10(1):1-9.

25. Sausman C, Oborn E, Barrett M. Policy translation through localisation: implementing national policy in the UK. *Policy & Politics* 2016;44(4):563-89.

26. Morton K, Voss S, Adamson J, et al. General practitioners and emergency departments (GPED)—efficient models of care: a mixed-methods study protocol. *BMJ open* 2018;8(10):e024012.

27. May, C.R., Albers, B., Bracher, M. *et al.* Translational framework for implementation evaluation and research: a normalisation process theory coding manual for qualitative research and instrument development. *Implementation Sci* **17,** 19 (2022). https://doi.org/10.1186/s13012-022-01191-x

28. McEvoy R, Ballini L, Maltoni S, O'Donnell CA, Mair FS, MacFarlane A. A qualitative systematic review of studies using the normalization process theory to research implementation processes. Implement Sci. 2014;9:2.

29. May C, Cummings A, Girling M, Bracher M, Mair FS, et al. Using normalization process theory in feasibility studies and process evaluations of complex healthcare interventions: a systematic review. Implement Sci. 2018;13(1):80.



For peer review only - http://bmjopen.bmj.com/site/about/guidelines.xhtml

30. Sheard L, Marsh C. How to analyse longitudinal data from multiple sources in qualitative health research: the pen portrait analytic technique. *BMC medical research methodology* 2019;19(1):1-10.

31. Braun V, Clarke V. Reflecting on reflexive thematic analysis. *Qualitative research in sport, exercise and health* 2019;11(4):589-97.

32. Scantlebury A, Brant H, Anderson H, et al. Potential impacts of general practitioners working in or alongside emergency departments in England: initial qualitative findings from a national mixed-methods evaluation. *BMJ open* 2021;11(5):e045453.

33. NHS Digital. Hospital Accident & Emergency Acitivity 2020-21, 2021.

34. Gaughan J, Liu D, Gutacker N, et al. Does the presence of general practitioners in emergency departments affect quality and safety in English NHS hospitals? A retrospective observational study. *BMJ Open* 2022;12(2):e055976. doi: 10.1136/bmjopen-2021-055976

35. Benger JR, H; Scantlebury, A; Anderson, H; Baxter, H; Bloor, K; Brandling, J; Cowlishaw, S; Doran, T; Gaughan, J; Gibson, A; Gutacker, N; Leggett, H; Liu, D; Morton, K; Purdy, S; Salisbury, C; Vaittinen, A; Voss, S; Watson, R; Adamson, J;. General Practitioners working in or alongside the emergency department: the GPED mixed-methods study. *Health Services and Delivery Research Program* In press

36. O'Cathain A, Murphy E, Nicholl J. Three techniques for integrating data in mixed methods studies. *Bmj* 2010;341

37. Anderson H, Scantlebury A, Leggett H, et al. Factors influencing streaming to General Practitioners in emergency departments: A qualitative study. *International Journal of Nursing Studies* 2021;120:103980.

38. Brant H, Voss S, Morton K, et al. Current provision of general practitioner services in or alongside emergency departments in England. *Emergency Medicine Journal* 2021;38(10):780-83.

39. Cooper A, Edwards M, Brandling J, et al. Taxonomy of the form and function of primary care services in or alongside emergency departments: concepts paper. *Emergency Medicine Journal* 2019;36(10):625-30.

40. The Kings Fund. What's going on with A&E waiting times? Online, 2020.

41. Kool RB, Homberg DJ, Kamphuis HC. Towards integration of general practitioner posts and accident and emergency departments: a case study of two integrated emergency posts



For peer review only - http://bmjopen.bmj.com/site/about/guidelines.xhtml

in the Netherlands. BMC Health Serv Res 2008;8:225. https://doi.org/10.1186/1472-6963-8-225

42. Dale J, Green J, Reid F, Glucksman E, Higgs R. Primary care in the accident and emergency department: II. Comparison of general practitioners and hospital doctors. BMJ 1995;311:427–30. https://doi.org/10.1136/bmj.311.7002.427

43. Wang M, Wild S, Hilfiker G, Chmiel C, Sidler P, Eichler K, et al. Hospital-integrated general practice: a promising way to manage walk-in patients in emergency departments. J Eval Clin Pract 2014;20:20–6. https://doi.org/10.1111/jep.12074

44. Boeke AJ, van Randwijck-Jacobze ME, de Lange-Klerk EM, Grol SM, Kramer MH, van der Horst HE. Effectiveness of GPs in accident and emergency departments. Br J Gen Pract 2010;60:e378–84. https://doi.org/10.3399/bjgp10X532369

45. Carson D, Clay H, Stern R. Primary Care and Emergency Departments: Report From the Primary. Care Foundation. Mar 2010. London: Primary Care Foundation; 2010.

46. Bickerton J, Davies J, Davies H, Apau D, Procter S. Streaming primary urgent care: a prospective approach. Prim Health Care Res Dev 2012;13:142–52. https://doi.org/10.1017/S146342361100017X

47. Uthman OA, Walker C, Lahiri S, Jenkinson D, Adekanmbi V, Robertson W, Clarke A. General practitioners providing non-urgent care in emergency department: a natural experiment. BMJ Open 2018;8:e019736. https://doi.org/10.1136/bmjopen-2017-019736

48. Ablard S, Kuczawski M, Sampson FC, Mason SM. What does the ideal urgent and emergency care system look like? A qualitative study of service user perspectives. Emerg Med J 2020;37:200–5. https://doi.org/10.1136/emermed-2019-208921

49. Ioannidis JPA. Contradicted and Initially Stronger Effects in Highly Cited Clinical Research. JAMA. 2005;294(2):218–228. doi:10.1001/jama.294.2.218

50. Salisbury C, Hollinghurst S, Montgomery A, et al. The impact of co-located NHS walk-in centres on emergency departments. *Emergency Medicine Journal* 2007;24(4):265-69.

51. Pope C, Turnbull J, Jones J, et al. Has the NHS 111 urgent care telephone service been a success. Case study and secondary data analysis in England. *BMJ open* 2017;7(5):e014815.

52. Blunt I, Edwards N, Merry L. What's Behind the A&E 'Crisis'? London: Nuffield Trust; 2015.

For peer review only

53. Oliver D. David Oliver: Why force GP streaming on NHS emergency departments? BMJ 2020;368:m992. https://doi.org/10.1136/bmj.m992

54. Edwards A. Evaluating effectiveness, safety, patient experience and system implications of different models of using GPs in or alongside Emergency Departments. *London: National Institute for Health Research (NIHR)* 2017

For peer review only



For peer review only - http://bmjopen.bmj.com/site/about/guidelines.xhtml

# GPED –Coding Framework

| | |
|---|---|
| NATIONAL CONTEXT | National - policy, pressures |
| LOCAL CONTEXT | Local – service landscape and population/specific local needs/considerations |
| TRUST ED & UC CULTURE | RESPOND OR RESIST whether staff are actively redirecting patients away from the ED to resist the flow or providing a service in response and recognition that patients have attended with health concerns. Where staff feel they must see patients and responsibility rests with them to provide health care. UCC/GP in ED vs primary care (differences to traditional primary care role. |
| PEN PORTRAIT DATA | Explanation of current system, patient journey through the ED, Layout, History of GPED, future plans, |
| PATIENTS REASONS FOR ATTENDING ED | Patient and staff explanations of why patients attend ED/Previous use of services e.g. have they seen/contacted service before ED |
| SERVICE LITERACY | Any discussions around appropriate/inappropriate attendances, perceived impact of service literacy and actual patient service literacy on use of GPED/ED |
| IMPLEMENTATION | Perceived Challenges and Facilitators to Implementation |
| PERCEIVED IMPACT | Perceived impact of GPED on patient safety, workforce and skills mix, staff interactions, performance/targets, views of GPED |
| EXPECTATIONS OF GPED (T1) | 'hypothesis' from stakeholders at all levels regarding their expectations of what would be the outcome of introduction to GPED. From T1 data, only prospective? |
| OTHER/MISCELLANEOUS INSIGHTS | Potential emerging insights which are outside the current framework but may be significant/to be reviewed with the WPC team regular meetings. |

## Standards for Reporting Implementation Studies: the StaRI checklist for completion

The StaRI standard should be referenced as: Pinnock H, Barwick M, Carpenter C, Eldridge S, Grandes G, Griffiths CJ, Rycroft-Malone J, Meissner P, Murray E, Patel A, Sheikh A, Taylor SJC for the StaRI Group. Standards for Reporting Implementation Studies (StaRI) statement. *BMJ* 2017;356:i6795

The detailed Explanation and Elaboration document, which provides the rationale and exemplar text for all these items is: Pinnock H, Barwick M, Carpenter C, Eldridge S, Grandes G, Griffiths C, Rycroft-Malone J, Meissner P, Murray E, Patel A, Sheikh A, Taylor S, for the StaRI group. Standards for Reporting Implementation Studies (StaRI). Explanation and Elaboration document. *BMJ Open* 2017 2017;7:e013318

Notes: A key concept of the StaRI standards is the dual strands of describing, on the one hand, the implementation strategy and, on the other, the clinical, healthcare, or public health intervention that is being implemented. These strands are represented as two columns in the checklist.

| | |
|---|---|
| The primary focus of implementation science is the implementation strategy (column 1) and the expectation is that this will always be completed. | The evidence about the impact of the intervention on the targeted population should always be considered (column 2) and either health outcomes reported or robust evidence cited to support a known beneficial effect of the intervention on the health of individuals or populations. |

The StaRI standardsrefers to the broad range of study designs employed in implementation science. Authors should refer to other reporting standards for advice on reporting specific methodological features. Conversely, whilst all items are worthy of consideration, not all items will be applicable to, or feasible within every study.

| Checklist item | | Reported on page # | Implementation Strategy | Reported on page # | Intervention |
|---|---|---|---|---|---|
| | | | "Implementation strategy" refers to how the intervention was implemented | | "Intervention" refers to the healthcare or public health intervention that is being implemented. |
| **Title and abstract** | | | | | |
| Title | **1** | 1 | Identification as an implementation study, and description of the methodology in the title and/or keywords | | |
| Abstract | **2** | 3 | Identification as an implementation study, including a description of the implementation strategy to be tested, the evidence-based intervention being implemented, and defining the key implementation and health outcomes. | | |
| **Introduction** | | | | | |
| Introduction | **3** | 4 | Description of the problem, challenge or deficiency in healthcare or public health that the intervention being implemented aims to address. | | |
| Rationale | **4** | 4, 5 | The scientific background and rationale for the implementation strategy (including any underpinning theory/framework/model, how it is expected to achieve its effects and any pilot work). | | The scientific background and rationale for the intervention being implemented (including evidence about its effectiveness and how it is expected to achieve its effects). |

**Commented [a1]:** This doesn't work



For peer review only - http://bmjopen.bmj.com/site/about/guidelines.xhtml

| | | | | | |
|---|---|---|---|---|---|
| Aims and objectives | **5** | 4 | The aims of the study, differentiating between implementation objectives and any intervention objectives. | | |
| **Methods: description** | | | | | |
| Design | **6** | 4 - 8 | The design and key features of the evaluation, (cross referencing to any appropriate methodology reporting standards) and any changes to study protocol, with reasons | | |
| Context | **7** | 3, 6, 8, 14, 15 | The context in which the intervention was implemented. (Consider social, economic, policy, healthcare, organisational barriers and facilitators that might influence implementation elsewhere). | | |
| Targeted 'sites' | **8** | 3, 6-8 | The characteristics of the targeted 'site(s)' (e.g locations/personnel/resources etc.) for implementation and any eligibility criteria. | | The population targeted by the intervention and any eligibility criteria. |
| Description | **9** | 4, 11-19 | A description of the implementation strategy | | A description of the intervention |
| Sub-groups | **10** | N/A | Any sub-groups recruited for additional research tasks, and/or nested studies are described | | |
| **Methods: evaluation** | | | | | |
| Outcomes | **11** | 3, 6, 7, 8, 9 | Defined pre-specified primary and other outcome(s) of the implementation strategy, and how they were assessed. Document any pre-determined targets | | Defined pre-specified primary and other outcome(s) of the intervention (if assessed), and how they were assessed. Document any pre-determined targets |
| Process evaluation | **12** | N/A | Process evaluation objectives and outcomes related to the mechanism by which the strategy is expected to work | | |
| Economic evaluation | **13** | 8 | Methods for resource use, costs, economic outcomes and analysis for the implementation strategy | | Methods for resource use, costs, economic outcomes and analysis for the intervention |
| Sample size | **14** | 6-8 | Rationale for sample sizes (including sample size calculations, budgetary constraints, practical considerations, data saturation, as appropriate) | | |
| Analysis | **15** | 3, 6, 8 | Methods of analysis (with reasons for that choice) | | |
| Sub-group analyses | **16** | 6-8 | Any a priori sub-group analyses (e.g. between different sites in a multicentre study, different clinical or demographic populations), and sub-groups recruited to specific nested research tasks | | |
| **Results** | | | | | |



For peer review only - http://bmjopen.bmj.com/site/about/guidelines.xhtml

| | | | | | |
|---|---|---|---|---|---|
| Characteristics | **17** | 6, 7 | Proportion recruited and characteristics of the recipient population for the implementation strategy | | Proportion recruited and characteristics (if appropriate) of the recipient population for the intervention |
| Outcomes | **18** | 11-13 14-19 | Primary and other outcome(s) of the implementation strategy | | Primary and other outcome(s) of the Intervention (if assessed) |
| Process outcomes | **19** | N/A | Process data related to the implementation strategy mapped to the mechanism by which the strategy is expected to work | | |
| Economic evaluation | **20** | 13, 18-19 | Resource use, costs, economic outcomes and analysis for the implementation strategy | | Resource use, costs, economic outcomes and analysis for the intervention |
| Sub-group analyses | **21** | 11-19 | Representativeness and outcomes of subgroups including those recruited to specific research tasks | | |
| Fidelity/ adaptation | **22** | 11-19 | Fidelity to implementation strategy as planned and adaptation to suit context and preferences | | Fidelity to delivering the core components of intervention (where measured) |
| Contextual changes | **23** | 11-19 | Contextual changes (if any) which may have affected outcomes | | |
| Harms | **24** | N/A | All important harms or unintended effects in each group | | |
| **Discussion** | | | | | |
| Structured discussion | **25** | 19-24 | Summary of findings, strengths and limitations, comparisons with other studies, conclusions and implications | | |
| Implications | **26** | 20-24 | Discussion of policy, practice and/or research implications of the implementation strategy (specifically including scalability) | | Discussion of policy, practice and/or research implications of the intervention (specifically including sustainability) |
| **General** | | | | | |
| Statements | **27** | 5, 24-26 | Include statement(s) on regulatory approvals (including, as appropriate, ethical approval, confidential use of routine data, governance approval), trial/study registration (availability of protocol), funding and conflicts of interest | | |



For peer review only - http://bmjopen.bmj.com/site/about/guidelines.xhtml

BMJ Open

# Do General Practitioners Working in or Alongside the Emergency Department Improve Clinical Outcomes or Experience? A mixed methods study

| | |
|---|---|
| Journal: | *BMJ Open* |
| Manuscript ID | bmjopen-2022-063495.R2 |
| Article Type: | Original research |
| Date Submitted by the Author: | 30-Aug-2022 |
| Complete List of Authors: | Scantlebury, Arabella; University of York Department of Health Sciences, York Trials Unit<br>Adamson, Joy; University of York, Department of Health Sciences<br>Salisbury, Chris; University of Bristol, Centre for Academic Primary Care, School of Social and Community Medicine<br>Brant, Heather; University of the West of England Faculty of Health and Applied Sciences<br>Anderson, Helen; University of York Department of Health Sciences<br>Baxter, Helen; University of Bristol, School of Social and Community Medicine<br>Bloor, Karen; University of York, Department of Health Sciences<br>Cowlishaw, Sean; The University of Melbourne, Department of Psychiatry ; University of Bristol,<br>Doran, Tim; University of York<br>Gaughan, James; University of York Department of Health Sciences<br>Gibson, Andy; University of the West of England Faculty of Health and Applied Sciences<br>Gutacker, Nils; University of York, Centre for Health Economics<br>Leggett, Heather; University of York Department of Health Sciences<br>Purdy, Sarah; University of Bristol School of Social and Community Medicine<br>Voss, Sarah; University of the West of England Faculty of Health and Applied Sciences<br>Benger, Jonathan; University of the West of England Faculty of Health and Applied Sciences |
| <b>Primary Subject Heading</b>: | Emergency medicine |
| Secondary Subject Heading: | General practice / Family practice |
| Keywords: | Health policy < HEALTH SERVICES ADMINISTRATION & MANAGEMENT, ACCIDENT & EMERGENCY MEDICINE, QUALITATIVE RESEARCH, PRIMARY CARE |
| | |

SCHOLARONE™
Manuscripts

*I, the Submitting Author has the right to grant and does grant on behalf of all authors of the Work (as defined in the below author licence), an exclusive licence and/or a non-exclusive licence for contributions from authors who are: i) UK Crown employees; ii) where BMJ has agreed a CC-BY licence shall apply, and/or iii) in accordance with the terms applicable for US Federal Government officers or employees acting as part of their official duties; on a worldwide, perpetual, irrevocable, royalty-free basis to BMJ Publishing Group Ltd ("BMJ") its licensees and where the relevant Journal is co-owned by BMJ to the co-owners of the Journal, to publish the Work in this journal and any other BMJ products and to exploit all rights, as set out in our licence.*

*The Submitting Author accepts and understands that any supply made under these terms is made by BMJ to the Submitting Author unless you are acting as an employee on behalf of your employer or a postgraduate student of an affiliated institution which is paying any applicable article publishing charge ("APC") for Open Access articles. Where the Submitting Author wishes to make the Work available on an Open Access basis (and intends to pay the relevant APC), the terms of reuse of such Open Access shall be governed by a Creative Commons licence – details of these licences and which Creative Commons licence will apply to this Work are set out in our licence referred to above.*

*Other than as permitted in any relevant BMJ Author's Self Archiving Policies, I confirm this Work has not been accepted for publication elsewhere, is not being considered for publication elsewhere and does not duplicate material already published. I confirm all authors consent to publication of this Work and authorise the granting of this licence.*

For peer review only

# Do General Practitioners Working in or Alongside the Emergency Department Improve Clinical Outcomes or Experience? A mixed methods study

**Authors names:** Arabella Scantlebury (0000-0003-3518-2740), Joy Adamson (0000-0002-9860-0850), Chris Salisbury (0000-0002-4378-3960), Heather Brant (0000-0001-9608-7451), Helen Anderson (0000-0002-6945-0590), Helen Baxter (0000-0002-3320-2915), Karen Bloor (0000-0003-4852-9854), Sean Cowlishaw (0000-0002-8523-3713), Tim Doran (0000-0001-7857-3704), James Gaughan (0000-0002-8409-140X), Andy Gibson (0000-0002-4641-2583), Nils Gutacker (0000-0002-2833-0621), Heather Leggett (0000-0001-8708-9842), Sarah Purdy (0000-0002-3445-986X), Sarah Voss (0000-0001-5044-5145), Jonathan Richard Benger (0000-0001-6131-0916).

York Trials Unit, Department of Health Sciences, ARRC Building, University of York, York, YO10 5DD, UK, Assistant Professor, Arabella Scantlebury

York Trials Unit, Department of Health Sciences, ARRC Building, University of York, York, YO10 5DD, UK, Professor, Joy Adamson

School of Social and Community Medicine, University of Bristol, Bristol, BS8 2PS, UK, Professor, Chris Salisbury

Faculty of Health and Applied Sciences, University of the West of England, Bristol, BS16 1QY UK, Senior Research Associate, Heather Brant

York Trials Unit, Department of Health Sciences, ARRC Building, University of York, York, YO10 5DD, UK, Research Fellow, Helen Anderson

School of Social and Community Medicine, University of Bristol, Bristol, BS8 2PS, UK, Research Fellow, Helen Baxter

Department of Health Sciences, ARRC Building, University of York, York, YO10 5DD, UK, Professor, Karen Bloor

Department of Psychiatry, University of Melbourne, Melbourne, VIC 3053, Australia, Senior Research Fellow, Sean Cowlishaw

Department of Health Sciences, ARRC Building, University of York, York, YO10 5DD, UK, Professor, Tim Doran

Department of Health Sciences, ARRC Building, University of York, York, YO10 5DD, UK, Research Fellow, James Gaughan

For peer review only

Faculty of Health and Applied Sciences, University of the West of England, Bristol, BS16 1QY, Associate Professor, Andrew Gibson

Department of Health Sciences, ARRC Building, University of York, York, YO10 5DD, UK, Senior Research Fellow, Nils Gutacker

York Trials Unit, Department of Health Sciences, ARRC Building, University of York, York, YO10 5DD, UK, Research Fellow, Heather Leggett

School of Social and Community Medicine, University of Bristol, Bristol, BS8 2PS, UK, Professor, Sarah Purdy

Faculty of Health and Applied Sciences, University of the West of England, Bristol, BS16 1QY, Professor, Sarah Voss

Faculty of Health and Applied Sciences, UWE Bristol, Glenside Campus, Blackberry Hill, Bristol, BS16 1DD, Professor, Jonathan Benger

**Correspondence to:** Jonathan Benger, Faculty of Health and Applied Sciences, University of the West of England, Bristol, BS16 1QY. Jonathan.Benger@uwe.ac.uk

For peer review only

For peer review only



For peer review only - http://bmjopen.bmj.com/site/about/guidelines.xhtml

# Abstract

**Objectives:** To examine the effect of General Practitioners (GPs) working in or alongside the Emergency Department (GPED) on patient outcomes and experience, and the associated impacts of implementation on the workforce.

**Design:** Mixed-methods study: interviews with service leaders and NHS managers; in-depth case studies (n=10) and retrospective observational analysis of routinely collected national data. We used Normalisation Process Theory to map our findings to the theory's four main constructs of coherence, cognitive participation, collective action and reflexive monitoring.

**Setting and participants:** Data was collected from 64 Emergency Departments (ED) in England. Case site data included: non-participant observation of 142 clinical encounters; 413 semi-structured interviews with policy makers, service leaders, clinical staff, patients and carers. Retrospective observational analysis used routinely collected Hospital Episode Statistics alongside information on GPED service hours from 40 hospitals for which complete data were available.

**Results:** There was disagreement at individual, stakeholder and organisational levels regarding the purpose and potential impact of GPED (coherence). Participants criticised policy development and implementation, and staff engagement was hindered by tensions between ED and GP staff (cognitive participation). Patient "streaming" processes, staffing and resource constraints influenced whether GPED became embedded in routine practice. Concerns that GPED may increase ED attendance influenced staff views. Our quantitative analysis showed no detectable impact on attendance (collective action). Stakeholders disagreed whether GPED was successful, due to variations in GPED model, site-specific patient mix and governance arrangements. Following statistical adjustment for multiple testing, we found no impact on: ED re-attendances within seven days, patients discharged within four hours of arrival, patients leaving the ED without being seen; inpatient admissions; non-urgent ED attendances and 30-day mortality (reflexive monitoring).

**Conclusions:** We found a high degree of variability between hospital sites, but no overall evidence that GPED increases the efficient operation of EDs or improves clinical outcomes, patient or staff experience.


**Trial registration:** ISCRTN5178022



For peer review only - http://bmjopen.bmj.com/site/about/guidelines.xhtml

## Strengths and limitations of this study

- National evaluation of the impact of general practitioners working in or alongside emergency departments in England.
- Mixed methods approach using a large qualitative data set (413 interviews, 142 non-participant observation) and routine national data sets involving multiple stakeholders across 64 emergency departments gave us a service wide and detailed understanding of the impact of GPED.
- Our data apply to England only and so may not be generalizable to other countries and healthcare settings.
- Our quantitative analysis was limited to routinely available data and so our analysis was dependent on key performance indicators and what is routinely collected and reported.

## Introduction

There were almost 24 million attendances at hospital emergency departments (EDs) in England in 2017-18, an increase of 22% since 2007/8.[1] This continues a long-term trend of increasing demand for urgent care at EDs that has also been observed in many other countries.[2] Workload pressures within these departments can lead to adverse effects on the quality of patient care, patient safety, clinical outcomes, patient satisfaction and staff job satisfaction.[3] One important measure of the performance of emergency departments in England is the target that 95% of patients should be admitted, transferred or discharged within four hours of arrival. This target has not been met nationally since 2015, with performance declining every year.[1]

About a fifth of patients attending emergency departments could be managed by general practitioners (GPs) in primary care settings, although estimates of this proportion vary widely depending on the definitions used.[4] Research suggests that the reasons patients choose to attend an emergency department with problems suitable for General Practice include: the perceived urgency of the situation, the belief that they need care only available in hospitals, the convenience of obtaining care at any time without an appointment, barriers to accessing general practice, and a lack of awareness of available primary care services.[5-7]

Several different policy initiatives have been proposed to address rising ED demand, and to allow EDs to focus on patients with the most urgent need.[8-10] These responses fall into three main categories: a triage step before patients attend EDs, such as a telephone advice line or "streaming" at the front door of the ED to direct patients to alternative services off-site;



For peer review only - http://bmjopen.bmj.com/site/about/guidelines.xhtml

better provision of alternative services (such as nurse-led walk-in services and Urgent Treatment Centres); improved access to GP services for people attending EDs. The latter approach can be achieved either by co-locating GP services alongside EDs at hospital sites, or by employing GPs to work within EDs to see selected patients. It has been suggested that GPs in or alongside the ED have the potential to improve patient care, and to reduce waiting times, unnecessary investigations, hospital admission rates, and costs,[11] but evidence to substantiate these claims is limited.[12-16] The introduction of these services was accelerated in 2017, when the UK government provided £100million of capital funding to support hospitals in England to provide a GP working in or alongside the ED,[17-19] as part of a comprehensive plan to reduce the growth of lower acuity patients attending EDs.[10] The aim of our research was to examine the effect of General Practitioners working in or alongside the Emergency Department (GPED) on patient outcomes and experience and the associated workforce and system impact. To incorporate all aspects of the research – both evaluative and the experiences associated with implementation – we have situated our work in the Normalisation Process Theory (NPT) framework.[20.21]

## Methods

### Design

We completed a mixed methods study including interviews with service leaders and NHS managers, in-depth case studies and a retrospective observational analysis of routinely collected national data. This approach enabled us to obtain a service-wide understanding of the impact of GPED on the urgent care system, the associated workforce and patient care.[22-25] Details of the study methodology have been published previously.[26]

Ethics committee approval was obtained from East Midlands – Leicester South Research Ethics Committee (ref: 17/EM/0312); University of Newcastle Ethics Committee (Ref: 14348/2016) and the Health Research Authority (IRAS: 230848 and 218038).

### Theoretical approach

We drew on NPT, which has been widely used to understand how and why things do or do not become embedded into routine practice.[20] Through its four core constructs of coherence, cognitive participation, collective action and reflexive monitoring (Box 1).[20,21] NPT can support both the understanding *and* evaluation of the implementation of organisational innovations such as GPED.[27] Its use has been supported by empirical studies using both qualitative and quantitative methods – therefore it was a particularly useful framework to apply in this context, given our study aims.[28,29]



For peer review only - http://bmjopen.bmj.com/site/about/guidelines.xhtml

**Box 1: The four core constructs of NPT, adapted for use in the GPED study**

**Coherence:** Do staff understand why GPED has been implemented?

**Cognitive participation:** Are staff engaged and committed to GPED, and what are the factors that promote and/or inhibit this commitment?

**Collective action:** Are participants using GPED and what are the factors that promote and/or inhibit them from using GPED?

**Reflexive monitoring:** Have staff appraised GPED and its impact on practice?

NPT enabled us to integrate our qualitative and quantitative data; examining the extent to which GPED had become a part of routine practice and highlighting the related impact on patients and staff.

## Qualitative data collection and analysis

Qualitative data collection (Table 1) consisted of non-participant observation of 142 individual clinical encounters and 467 semi-structured interviews with key stakeholders (policymakers, service leaders, ED staff, General Practitioners, patients and carers). Qualitative data was distributed across 64 NHS EDs in England, 10 of which were in-depth case study sites. Data collection explored the impact of GPED from the perspectives of key stakeholders as well as the policy's background and factors affecting implementation (see Supplementary files 1 and 2 for example topic guides). Following initial familiarisation and independent coding, the qualitative team, through a series of roundtable discussions and workshops with our patient collaborators, developed a coding framework (additional file 1). The coding framework, in conjunction with pen portraits of our ten case sites,[30] was used to facilitate cross-case comparisons and formed the basis of our main thematic analysis.[31] Initial analysis identified ten key themes (Table 2) – these included *Contested Policy*, which reflected stakeholder views on the concept of GPED and *Structural Implementation* relating to site level responses to the introduction of GPED. In addition, we identified eight themes which were factors our participants predicted would be affected by GPED (at time 1 qualitative data collection): *Performance against the four hour target; Use of investigations; Hospital admission; Patient outcome and experience; Service access; Staff recruitment and retention; Workforce behaviour and experience; Resource use*. We have collectively termed these eight themes as



For peer review only - http://bmjopen.bmj.com/site/about/guidelines.xhtml

‘domains of influence’,[32] which we have then used as outcome measures in our evaluation of GPED.

For peer review only



For peer review only - http://bmjopen.bmj.com/site/about/guidelines.xhtml

Table 1 Qualitative data collection

| | Policymakers | Service leaders | | Case sites | | |
|---|---|---|---|---|---|---|
| | Time 1 | Time 1 | Time 2 | Time 1 | Time 2 | Time 3 |
| Type of data collected | Semi-structured telephone interviews | Semi-structured telephone interviews | Semi-structured telephone interviews | Semi-structured face-to-face and telephone interviews, non-participant observations. | Semi-structured face-to-face and telephone interviews | Semi-structured face-to-face and telephone interviews, non-participant observations. |
| Aim of data collection | In-depth understanding of GPED policy and implementation from key informants | Broad perspective of GPED implementation and current provision from a range of EDs | Broad perspective of GPED implementation and current provision from a range of EDs | In-depth understanding from a small number of case sites | Brief 'check in' visits to assess any interim changes in GPED services | In-depth understanding from a small number of case sites |
| Period of data collection | December 2017 to January 2018 | August 2017-September 2018 | February 2018- February 2019 | November 2017-December 2018 | June-October 2018 | November 2018-December 2019 |
| Number of EDs | Not applicable | 64 | 30 | 10 | 5 | 10 |
| Stakeholder groups and organisations represented | NHS England and Improvement, Department of health, Clinical Commissioning Groups, NHS Trusts, Royal College of Emergency Medicine, GPs | Chief Executives, Chief Operating Officers, Clinical Leads, Lead nurses and ED managers | Chief Executives, Chief Operating Officers, Clinical Leads, Lead nurses and ED managers | GPs, ED doctors (juniors, registrars, consultants), Nurses (streaming, triage, emergency nurse practitioner), patients and carers | GPs, ED doctors (juniors, registrars, consultants), Nurses (streaming, triage, emergency nurse practitioner), patients and carers | GPs, ED doctors (juniors, registrars, consultants), Nurses (streaming, triage, emergency nurse practitioner), patients and carers |
| Total number of participants | 10 policymakers | 57 service leaders | 26 service leaders | 124 health professionals 94 patients/ carers<br>83 non-participant observations. | 20 health professionals | 82 health professionals, 54 patients/carers, 59 non-participant observations. |

*GPED: General Practitioners working in or alongside the Emergency Department; ED: Emergency Department; GP: General Practitioner.*



For peer review only - http://bmjopen.bmj.com/site/about/guidelines.xhtml

## Quantitative data and analysis

We completed a retrospective observational analysis of routinely collected Hospital Episode Statistics (HES) data between April 2018 and March 2019 from 40 English hospitals that were selected for their ability to provide complete data on the times of day when GPED services were available. Differences in GPED service availability between EDs at the same time of day were used to assign patients quasi-randomly to treatment or control groups at each hour of the day. Outcomes measured were: percentage of patients discharged within four hours of arrival; ED attendances that resulted in hospital admission; patients who left without being seen; unplanned re-attendance at the ED within 7 days; 30 day mortality; non-urgent ED attendances (described previously as ‘unnecessary’ and identified using a defined methodology);[33] volume of ED attendances. Each outcome was analysed separately using two-way fixed effects. Outcomes for patients attending different EDs at the same time of day were compared, exploiting variation in the timing of availability of GPED within the day at different EDs. Further details of this analysis have been published previously.[34] The potential net cost savings were explored using a comparative approach based on the results of this analysis.[35] We also conducted a survey of the GPED workforce at our 10 case sites, however, as these results did not materially alter our overall findings they are not reported here.[35]

## Mixed methods analysis

In addition to individual quantitative and qualitative analyses, we conducted higher-level synthesis to integrate the study findings using a triangulation protocol that combined different methods to gain a more complete picture.[36] Quantitative findings were grouped under the qualitative themes described above (Table 2). We then mapped our study findings onto the four core constructs of NPT (Tables 3-6).[20] Given the inter-related nature of the NPT constructs this process was undertaken by two researchers (JA and AS).

*Table 2 Qualitative and quantitative data integration*

| Theme | Qualitative | Quantitative |
|---|---|---|
| **Contested policy** | Qualitative interviews with policymakers and service leaders, health professionals, patients and carers. Non-participant observation | |



For peer review only - http://bmjopen.bmj.com/site/about/guidelines.xhtml

| | | |
|---|---|---|
| **Performance against the four-hour target** | Qualitative interviews with policymakers, health professionals, patients and carers. Non-participant observation | HES data: percentage of patients discharged within four hours of arrival |
| **Use of investigations** | Qualitative interviews with policymakers, health professionals, patients and carers. Non-participant observation | |
| **Hospital admissions** | Qualitative interviews with policymakers, health professionals, patients and carers. Non-participant observation | HES data: ED attendances that resulted in hospital admission |
| **Patient outcome and experience** | Qualitative interviews with policymakers, health professionals, patients and carers. Non-participant observation | HES data: patients who left without being seen<br>HES data: Unplanned re-attendance at the ED within 7 days<br>HES data: 30 day mortality |
| **Service access** | Qualitative interviews with policymakers, health professionals, patients and carers. Non-participant observation | HES data: non-urgent (described previously as 'unnecessary') ED attendances<br>HES data: Volume of attendances |
| **Staff (recruitment, retention)** | Qualitative interviews with policymakers, health professionals, patients and carers. Non-participant observation | |
| **Workforce (behaviour, experience)** | Qualitative interviews with policymakers, health professionals, patients and carers. Non-participant observation | |
| **Resource use** | Qualitative interviews with policymakers and service leaders, health professionals, patients and carers. Non-participant observation | |
| **Structural implementation** | Qualitative interviews with policymakers, health professionals, patients and carers. Non-participant observation | |

*ED: Emergency Department; HES: Hospital Episode Statistics.*

## Patient and public involvement

Patients and members of the public were involved throughout the development and delivery of this research. We formed a group of ten public contributors with a wide variety of



For peer review only - http://bmjopen.bmj.com/site/about/guidelines.xhtml

experiences of ED services. Throughout the study, the group were involved in regular workshops and meetings where they were asked to assist in interpreting the qualitative and quantitative data and support the development of our mixed methods synthesis. For instance, our lay contributors highlighted the central role played by the streaming nurse in GPED, which led to a further analysis of qualitative data surrounding streaming that has been published previously.[37] Two members of the group were also full members of the Study Steering Committee.

# Results

Tables 3-6 show how the themes from our qualitative and quantitative data map onto the four constructs of NPT.

### Coherence – do stakeholders have an understanding of why GPED was implemented?

For a health policy to be adopted into routine practice, there needs to be a shared sense of its purpose. Many stakeholders understood that GPED was being introduced as a direct response to rising pressures in EDs and as a potential mechanism for improving ED performance. Despite this, all stakeholder groups suggested that GPED was a rushed policy that lacked clear and consistent guidance. The fact that the policy was believed to originate largely from discussions between the Secretary of State for Health and NHS England, leading to "top down" implementation, and the lack of evidence supporting the clinical and cost-effectiveness of GPED were further causes of concern.

The decision to introduce GPED nationally was also based on the perceived success of a GPED service that had been implemented at a single NHS site – Luton and Dunstable (L&D). The rationale for choosing L&D as the national exemplar over other high-performing EDs was unclear, particularly given that it was difficult to determine whether the perceived success of L&D was due to GPED or the simultaneous introduction of other initiatives within the organisation. Associated with this were concerns that GPED failed to acknowledge local context and variations in demand for ED services, varying patient populations and pre-existing or prior attempts to implement GPED services.

This led to stakeholders questioning the generalisability of the national policy, and as a result GPED was interpreted differently with a range of models implemented throughout the NHS in England.[38,39]

There was widespread disagreement at an individual, stakeholder and organisational level about the purpose and potential impact of GPED. Despite disagreeing about the 'direction of effect,' stakeholders agreed on the areas of the healthcare system and patient care that GPED



For peer review only - http://bmjopen.bmj.com/site/about/guidelines.xhtml

was most likely to affect. We categorised these as eight themes as ‘domains of influence’ (Table 3),[32] which were subsequently used as the outcomes for our evaluation of GPED.

For peer review only



For peer review only - http://bmjopen.bmj.com/site/about/guidelines.xhtml

Table 3: Coherence - do stakeholders understand why GPED has been implemented?

| Questions | Themes | Illustrative data |
|---|---|---|
| Does GPED have a clear purpose and did participants have a shared sense of its this purpose?<br><br>Will GPED fit with the overall goals and activity of the organisation?<br><br>Is it clearly distinct from other interventions?<br>What benefits will the intervention bring and to whom? | ***Contested policy***<br>• The implementation of GPED was considered rushed, and to be based on conflicting guidance.<br>• Some stakeholders had difficulty understanding how GPED differed from other previously unsuccessful attempts to introduce GPs into the ED.<br>• It was uncertain how GPED, or the associated capital funding initiative, differed from previous and existing interventions.<br>• Variations in local context, ED demand and existing GP services in the ED resulted in GPED being interpreted and implemented differently.<br>***Domains of influence***<br>• GPED is difficult to describe, distinguish from other interventions and participants do not have a shared sense of its purpose.<br>• Stakeholders disagreed on the potential impacts of GPED, with positive, neutral or negative effects predicted for the majority of the eight identified domains of influence:  1) Performance against the four-hour target; 2) Use of investigations; 3) Hospital admissions; 4) Patient outcome and experience; 5) Service access; 6) Staff recruitment and retention, 7) Workforce behaviour and experience; 8) Resource use. | *"I think it adds to the mix. I think that it was not a very well thought through policy decision … It was never part of the urgent, the care, the Keogh review of urgent emergency care to have GPs in ED. Now that review focused much more on NHS 111 and also trying to create consistency … So having GPs in ED, was outside of that policy strand. So, and it was dropped in a very, at very great speed and without a great deal of thought." (Interview with service leader)*<br><br>*"You know, it isn't a sufficient evidence base to work from. You could have looked at the North East of England, I'm taking this call just now and said, you know, six of the top ten performers nationally sit in the North East, alright, and that tells us something about the system... and I think that, if we're going to use examples as a way of developing policy, that would have been a better way of looking at it." (Interview with Policymaker)*<br><br>*"Whilst we started with a very clear - here's the Luton model, it became, obviously when trusts came to implement it locally that due to various circumstances that were very specific to their trust and their community, the Luton model just wasn't appropriate. So, I think what we've ended up with is a range of different models. So, you couldn't look at GP streaming and say what we've got in place now is the same in every trust in the country because there's almost certainly ... there's huge variation in practice around how they're running." (Interview with policymaker)* |



For peer review only - http://bmjopen.bmj.com/site/about/guidelines.xhtml

Table 4: Cognitive participation - are people committed to using GPED and what are the factors that promote and/or inhibit this commitment?

| Questions | Themes | Illustrative data |
|---|---|---|
| Did stakeholders see the point easily?<br><br>Were stakeholders prepared to invest time, energy and work in GPED? | ***Contested policy***<br>• There was doubt whether GPED, as a single initiative, could fix complex problems in the healthcare system.<br>• GPED policy development was criticised, as was the fact that it was based on limited evidence and patient and clinical consultation. This reduced stakeholders' commitment to ensuring it was embedded into routine practice. | *"Because it [GPED] is cheaper than re-investing in social care. Preventing inappropriate admissions is right, but it doesn't solve all the problems in primary care - those patients that do need to be seen and do need support in the community/social care, [GPED] is not a long-term solution." (Interview with service leader)*<br><br>*"It [streaming criteria] should be fixed, but, as I said, depending on who you speak to, it does waver slightly on what practitioners and GPs are willing to see. So, it's a bit of a grey area really. It depends who you're working with really. I don't . . . yes. So, it's not fixed. It should be really." (Interview with Advanced Nurse Practitioner at Case site Redwood).*<br><br>*"Actually, looking at X-rays and ECGs is, it becomes a bit of a, a dying art in General Practice, if you're not looking at those sorts of things on a daily basis, and what we provide again is allowing GPs the ability to keep those sort of clinical skills up and running, when I think that, and I think that's the attractiveness about doing this." (Interview with Urgent Care Centre clinical lead at case site Teak)* |



For peer review only - http://bmjopen.bmj.com/site/about/guidelines.xhtml

Table 5: Collective action - are people using GPED and what are the factors that promote and/or inhibit them from using GPED?

| Questions | Themes | Illustrative data |
|---|---|---|
| What effect will GPED have on the ED and health service?<br>How will the intervention affect the work of patients and staff?<br><br>Will staff require further training?<br><br>What impact will it have on division of labour, resources, power and responsibility between different professional groups?<br><br>What are the factors that promote and/or inhibit them from using GPED? | ***Service Access***<br>• Despite reports that GPs have been working in the ED for some time, only a small number of patients reported using GPED previously and expected to be streamed to GPED.<br>• Staff were concerned that GPED may create 'easy access to a GP', encouraging people to attend.<br>• Staff were concerned that patients attended the ED "inappropriately", and considered poor health literacy to affect how patients use GPED.<br>• GPED and 'Urgent Care' were considered confusing to patients and made navigating services more challenging.<br>• Analysis of HES data identified no significant impact on: volume of ED attendances; number of non-urgent (described previously as 'unnecessary') attendances<br>***Staff recruitment and retention***<br>• Staffing issues posed a major threat to the successful implementation and adoption of GPED.<br>• Nursing shortages and a lack of experienced nurses made the staffing of streaming services challenging.<br>• Streaming may change the role of nurses and divert them away from core ED work, making GPED settings less attractive. The psychological and physical impact of streaming may negatively affect nurses' work and willingness to invest energy and time in GPED.<br>• GPED may draw GPs away from traditional General Practice. ED staff vacancies created issues in the recruitment of ED and GP staff.<br>• To overcome recruitment issues, GPED needs to be viewed as an attractive place to work.<br>The training and educational benefits that junior doctors may receive from working alongside GPED models were considered valuable, and may make them more committed to ensuring GPED is embedded into routine practice.<br>***Use of investigations***<br>• There was a lack of consensus as to whether GPED models should give GPs access to diagnostic testing, reflecting differing interpretations of the purpose of GPED and varying local needs. This | *"what appeals to me is that I can do a bit of acute general medicine, trauma etc. and I'm trained in that but equally, I can also lapse into what was my comfort zone … and that works really well whereas when I'm feeling a bit more sort of "right, come on, you know, I can get into resus and I can learn a new thing' and I really enjoy that." (Interview with GP at case site Juniper).*<br><br>*"The GP feels that one of the problems with the model, is that there is a need for experienced triage nurses in order for it to work, but the department has a high turnover of nursing staff and has difficulty retaining staff. There are only a couple of appointed nurses who have the experience required." (Interview with ED Consultant at case site Redwood).*<br><br>*"ED's frightened to send anything away, so everything comes in. So, I don't blame the public for attending if they can see a GP within three hours, rather than having to wait six, seven days or two weeks for an appointment. But I just wonder if it's made a demand for it, because you get people coming back to see the GP again in ED. (Interview with Nurse at case site Rowan)*<br><br>*"Patients are savvy as well, tell you what they think they want you to hear in order to get them into the service they want to be seen by." (Interview with Nurse at case site Linden).*<br><br>*"I think it's down to, obviously, your training, but also how risk averse you are, and some people are very risk averse and will just have a much lower* |



For peer review only - http://bmjopen.bmj.com/site/about/guidelines.xhtml

| | | |
|---|---|---|
| | caused tension between GP and ED staff and may make staff less likely to invest their time and energy into GPED.<br>***Workforce behaviour and experience***<br>• Good communication, trust and confidence between streaming staff and GPs are pivotal to the effectiveness of GPED.<br>• Staff were concerned about patients who attend the ED with conditions that could be treated in general practice, but had different perceptions of what constitutes a 'GPED appropriate patient.'<br>• Tensions between GPs and staff responsible for streaming decisions were common and reflected different attitudes to risk as well as staff members (ED and GP) protecting their own working environment – staff streamed patients to GPED, or back to ED during busy periods, to ease their respective workloads.<br>• Streaming protocols were developed to try to standardise streaming decisions and GPED acceptance criteria, however these were not consistently disseminated or followed.<br>***Structural implementation***<br>• Several implementation issues also affected the extent to which staff were able to embed GPED into their routine practice including structural support within the site, ensuring integrated information technology systems between ED and GPED and influencing factors relating to the GP's role such as ensuring a positive working environment and giving GPs access to investigations, where appropriate. | *threshold for streaming people into ED and then also the Urgent Care Centre, rather than directing appropriately, you know, taking that risk." (Interview with Paramedic at case site Chestnut).*<br><br>*"It's going on long enough to do and we really just didn't know what else to do. I literally can't drive. I'm having trouble getting out of the house. We could do it today and get here and try and figure out what was going on, rather than go to the GP, the GP say, "Do this, then come back," then almost probably end up in the hospital as it's going there anyway, to do the same things. That was the decision really." (Interview with patient at case site Hawthorn).* |

For peer review only

Table 6: Reflexive monitoring - have people appraised GPED and its impact on practice?

| Questions | Themes | Illustrative data |
|---|---|---|
| Will it be clear what effects the intervention has had?<br><br>How are users likely to perceive the intervention | ***Performance against the 4 hour target and hospital admissions***<br>• There was no significant impact on the proportion of patients meeting the four hour target, or on the number of attendances resulting in a hospital admission.<br>• Variations in site-specific patient mix, GPED models and whether patients streamed to GPED were included in ED reporting statistics, | *"Yeah, I think that's really important, I think given the way the hospital performs with the Government's four-hour target, I think it's a source of pride for the hospital for the Chief Exec." (Interview with ED Consultant at case site Linden).* |



For peer review only - http://bmjopen.bmj.com/site/about/guidelines.xhtml

| | | |
|---|---|---|
| once it has been in use for a while?<br><br>Is it likely to be perceived as advantageous for patients or staff? | combined with other factors that influence ED performance, may have contributed to the apparently limited effects of GPED.<br>***Resource use***<br>• Any possible cost savings due to reduced reattendances were much smaller than the cost of providing the service itself.<br>***Patient outcome and experience***<br>• Most patients saw the value of GPs working in or alongside the ED as long as they received appropriate care.<br>• Staff felt that GPED may negatively affect patient flow.<br>There was no significant impact on the following performance indicators in the HES analysis: left without being seen; 30-day mortality; re-attendance to the same ED within seven days. | *"I don't necessarily think it is a bad thing to have it, but it provides marginal gains, and those marginal gains are, happening at a very high capital cost and an ongoing staffing cost and looking at the NHS budget as a whole, I think it's a shocking waste of money." (Interview with ED consultant at case site Juniper)* |



For peer review only - http://bmjopen.bmj.com/site/about/guidelines.xhtml

### Cognitive participation – are people committed to using GPED and what are the factors that promote and/or inhibit this commitment?

The way in which GPED policy was designed and implemented, along with challenges in translating a national policy to meet local service and population needs, caused some to view GPED as a 'sticking plaster solution' to ED pressures. For many, the rise in ED attendances was driven by wider, more complex issues across health and social care, which were often deemed to be the result of deficiencies elsewhere in the system. As a result, there was doubt that a single initiative such as GPED could provide the solution. This lack of buy-in from stakeholders was reflected during interviews with service leaders and policymakers where alternative solutions for improving ED performance were proposed. For example, investment in social care and mental health services were considered to have a greater potential for impact.

Embedding GPED into existing practice requires commitment from key stakeholders. Emphasis was placed on the importance of streaming nurses and GPs working together to stream patients from ED to GPED. Despite many sites trying to ensure consistency through the development of streaming protocols, the challenges of disseminating and adhering to these protocols, reliance on locum and/or part-time GPs and frequent rotation of streaming nurses meant that the definition of a patient suitable for GPED varied between and within professional groups. This, combined with the cultural differences in how GPs and ED clinicians work, and their inherently different approaches to risk, was a source of tension that in some cases resulted in patients not being accepted by GPED and sent back to ED.

Whether GPED models gave GPs access to investigations such as x-rays and blood tests varied across case sites and reflected the different interpretations of the purpose of GPED and varying local contexts. Some individuals considered giving GPs access to investigations and diagnostic tests as crucial to the model's effectiveness by supporting GPs to treat a broader range of patients and refer to inpatient specialties. However, others felt that doing so asked GPs to work beyond their clinical competency – some staff felt that there was a shortage of GPs with the skills required to interpret some ED diagnostic tests, and an upskilling of the GP workforce would therefore be required. As a result, some GPs were asked to work as they would in general practice, whilst other services preferred those with prior ED experience.

### Collective action – are people using GPED and what are the factors that promote and/or inhibit them from using GPED?

At the time that GPED was introduced, general practice in England was facing a significant workforce crisis. This posed a real challenge both in terms of ensuring that EDs were able to recruit GPs to work in GPED and ensuring that in doing so workforce shortages elsewhere in



For peer review only - http://bmjopen.bmj.com/site/about/guidelines.xhtml

the system were not exacerbated. Site staff suggested that to facilitate the recruitment of GPs, emphasis should be placed on ensuring that GPED was considered an attractive place to work and on supporting GPs to work within the scope of their practice. However, whether GPED was viewed as a positive role depended on the individual GP. For instance, whilst GPED may be appealing to those who wish to expand their work beyond traditional general practice, the scope, acuity and shift-based working that are typical of the ED may contradict why many individuals chose to become a GP in the first place.

Ensuring that streaming is undertaken by experienced streaming nurses was also considered pivotal to an effective GPED service. However, nursing shortages, the psychological and physical burden of streaming on nurses and the potential for streaming to divert nurses away from their routine ED work meant that recruiting nurses to streaming roles was challenging.[37]

Our findings also identified several other factors that may promote or inhibit how staff use GPED, and the extent that it becomes embedded into routine practice (Table 7). These were categorised as those relating to; workforce behaviour and experience (communication, trust and role-based cultural differences) and streaming and implementation issues (streaming protocols, inter-professional relationships and structural support).

Service leaders and site staff were concerned that giving patients 'easy access' to a GP, in a climate where general practice appointments may be difficult to obtain, could encourage patients to attend the ED rather than their own GP. Staff were particularly critical of patients for what they considered 'inappropriate ED attendance' (i.e. attending the ED when they perceived alternative services would meet their needs). Whilst this was largely attributed to the potentially confusing range of services available, re-organisation and re-branding of existing services and perceived low levels of health literacy making service navigation difficult for patients, there were also some patients who were accused of deliberately 'playing the system'. For example, some patients were thought to deliberately bypass their GP and attend ED to access investigations, referrals or treatments. However, the reasons that patients chose to attend ED were complex, and in some cases, those that were considered by staff to have attended "inappropriately" had been advised to attend the ED by other healthcare professionals and services such as NHS111, a pharmacy or their own GP.

However, our qualitative data provided numerous examples of situations in which experienced nurses were unable to determine whether a patient's complaint should be treated by general practice or the ED, suggesting that it may be unreasonable to expect patients to make the 'correct' choice on every occasion.

Despite these concerns amongst site staff, analysis of HES data found no association between non-urgent attendances and GPED or the absolute and relative volume of



For peer review only - http://bmjopen.bmj.com/site/about/guidelines.xhtml

attendances and GPED.[34] Despite staff believing that GPED may encourage ED use, the qualitative data highlighted that patients attend the ED for a variety of reasons, and demonstrate reasoned decision-making in their service use. Only a small number of patients expected to see a GP, with the majority showing no awareness of GPED when interviewed. This is perhaps unsurprising given that sites often chose not to advertise GPED services to reduce the likelihood of driving an increase in ED attendances.

## Reflexive monitoring – have people appraised GPED and its impact on practice?

GPED is a complex intervention that has been introduced through a range of different models, into a complex and changing environment. EDs serve different patient populations and have different physical structures, staff mixes and care provision. In addition to this heterogeneity, the widespread uncertainty surrounding GPED operating hours and different governance arrangements across sites meant that there was variation in whether patients streamed to GPED were counted in nationally reported ED statistics. The challenges of using key performance indicators to evaluate national policies such as GPED was discussed by service leaders, who questioned their utility and described indicators such as the target that 95% of patients attending the ED should be admitted, transferred or discharged within four hours as 'blunt tools' for evaluating impact.

Our quantitative analysis showed no statistically significant improvement in a range of key performance indicators across several domains of influence including the "four-hour target", hospital admissions and patient outcomes and experience (patients leaving the ED without being seen and mortality at 30 days after an ED attendance). We did observe that GPED reduced the probability of unplanned re-attendance within seven days by 3.2% (OR: 0.968, 95% CI: 0.95 to 0.99), which equates to approximately 300 fewer re-attendances per year for an average ED in England. After adjustment for multiple testing, however, this difference was no longer statistically significant, and was also not judged to be clinically significant. Possible cost savings associated with reduced reattendances (£30-37,000 per ED per year) were heavily outweighed by the cost of GPED services. In the hospitals for which we had data, the average length of time of operation of a GPED service was 11.1 hours per day. Assuming only one GP is present and including salary costs of the GP alone (potentially a substantial underestimate), this amounts to around £454,000 per ED per year. As a result, current GPED models do not appear to be an efficient use of healthcare resources.[35]



For peer review only - http://bmjopen.bmj.com/site/about/guidelines.xhtml

The majority of patients we interviewed valued GPED and considered it beneficial to have GPs in EDs. Patients were aware that GPED may relieve pressure on the ED, ensuring emergency doctors can deal with the “real emergency cases” and were indifferent to the type of health professional that they saw as long as they received appropriate care. Similarly, the “four-hour target” was not a priority for patients, with many explaining that they were happy to wait longer as they understood that they were guaranteed to be seen and were waiting because priority was given to higher acuity patients. Despite this, staff raised concerns that GPED could negatively impact patient flow, as patients are required to disclose clinical information on multiple occasions before seeing a GP, which may create a backlog.

## Discussion

The GPED study was commissioned to evaluate the impact of GPs working in or alongside EDs; a national policy implemented in response to rising pressures on EDs in England. GPED had no effect on a range of routinely collected ED performance measures. Despite considerable concern from health professionals that GPED may actually increase demand, we found no significant effect of GPED on ED attendances or reattendances within 7 days. This was supported by our qualitative analysis; most of the patients that we interviewed were unaware of GPED and had not changed their behaviour as a result. We observed confusion amongst patients, staff, service leaders and participating NHS organisations as to the purpose of GPED, with a prevailing view that the main drivers of ED workload may be more related to an ageing population, high inpatient bed occupancy and a shortage of social care[40] than attendances by patients suitable for management in traditional general practice.

Early evaluations of GPED models of care in the UK and internationally suggested that placing GPs in the ED was a promising innovation.[41] Studies reported that GPED had the potential to reduce resource use,[42,43] and increase patient satisfaction.[44] Carson et al.[45] found that the proportion of cases seen by GPs varied and that clinical and operational governance was often disjointed. In a survey of patients, Bickerton et al.[46] found that whilst GPED offered patients a greater range of service provision, it also increased the risk of duplication and repeat attendance. More recently, in a relatively small study, Uthman et al. found that GPs who saw patients in the ED used fewer resources without increasing reattendance and referred more patients to follow-up services.[47] In addition, service users appreciated simplified health-care provision from a single point of access.[48]

It is not uncommon for early reports of new initiatives to be positive, but contradicted subsequently,[49] and our study is the largest of GPED services published to date. A similar phenomenon was observed previously in relation to nurse-led walk-in centres co-located with



For peer review only - http://bmjopen.bmj.com/site/about/guidelines.xhtml

For peer review only

the ED, whereby initially positive reports were challenged by a subsequent large-scale evaluation that found 'no evidence of any effect on attendance rates, process, costs or outcome of care'.[50] Furthermore, our data demonstrate considerable heterogeneity, with the implication that whilst our overall result is null, GPED may still have beneficial effects in some locations and under certain circumstances. Our findings suggest that GPED implementation is highly sensitive to local context, and these contexts will govern the success of any particular scheme. This is consistent with other evaluations of urgent and emergency care initiatives,[51] Investment in GPED appears justified only when the factors associated with success are in place (see below), and there is clear evidence of benefit at a local level. Where this evidence of local benefit is absent alternatives to GPED should be considered, such as improving provision and access in traditional General Practice, both in and out of hours.

Our quantitative analysis used routinely available data, and it would be surprising if some of these measures (e.g. 30 day mortality) were influenced by GPED. It has also been noted that patients eligible for GPED are often quick and easy to manage, do not breach the '4-hour target', are less likely to be admitted and do not contribute to crowding.[45,52] A recent realist review concluded that, despite GPs in ED being associated with a reduction in process time for non-urgent patients, this does not necessarily increase capacity to care for the sickest patients.[12] The main cause of ED crowding is perceived to be congestion in the flow of sicker patients into the hospital and a lack of beds, rather than absolute attendance numbers.[53]

The GPED study shows that even when a policy is mandatory and supported by dedicated capital funding, this does not guarantee successful or uniform adoption. Our findings highlight the complexities of translating policy into practice, and the importance of considering the extent that a government-led policy can be delivered at a local level. Previous evidence suggests that a common response to national policy is local adaptation, which can in turn lead to the implementation of different innovations to those that are originally proposed.[22] We found evidence of this, as interviewees often described a range of approaches to GPED that sometimes opposed the high-level policy messages that accompanied the provision of capital funding. It also remains uncertain whether revenue funding, as well as (or instead of) capital funding would have alleviated some of the noted challenges.

Our qualitative data also identified a range of factors that can facilitate implementation. We present these as a series of 'success factors' which may inform how services choose to implement future GPED models; or adapt existing ones (Table 7). At several of our case study sites, these fundamentals had been overlooked and the result was a less coherent GPED



For peer review only - http://bmjopen.bmj.com/site/about/guidelines.xhtml

service. However, it is important to note that even if all these ‘success factors’ are implemented, our findings do not present evidence that the resulting GPED service would have a positive impact on ED performance indicators or be cost-effective.

*Table 7 Success factors for the implementation of GPED.*

For peer review only



For peer review only - http://bmjopen.bmj.com/site/about/guidelines.xhtml

| Success factor | How can this be addressed? |
|---|---|
| **Streaming** | No single model for effective streaming was identified. The factors listed below should be considered when developing future streaming models. |
| *The experience and seniority of streaming nurses* | Effective streaming requires high levels of clinical knowledge, critical thinking, clinical decision-making and balancing clinical risks. Streaming should be undertaken by senior nurses. |
| *The skills, confidence and abilities of GPs* | Professional groups had different opinions as to what can be considered a "GP appropriate" patient. To alleviate tension between staff there needs to be a shared understanding of streaming protocols and an awareness of the skills and scope of practice of GPs. Recruiting experienced and clinically knowledgeable GPs who are willing to adapt and see a broader range of patients is helpful. |
| *Inter-professional relationships* | Trust and confidence between professional groups is essential. Co-location does not automatically ensure collaboration. Individuals naturally work within professional norms. Effective communication and common goals mitigate tension. |
| *Streaming protocols* | Stakeholder clinicians (including streamers and GPs) should be involved in the development and regular review of protocols. These should be effectively communicated to all relevant practitioners. For streaming to be effective, streamers may need to deviate from protocols based on their clinical judgment. Staff should be supported to do this, while also considering strategies to mitigate against inappropriate deviation which may negatively impact patient care. |
| *Streaming safety* | Safety concerns limit the effectiveness of streaming strategies and sources of support are needed to ensure staff feel confident in their decision making.<br>Clinicians should be involved in the development and regular review of protocols. These include effective pathways for managing deteriorating patients and returning streamed patients back to the ED when necessary. Consider ways to make the streaming process clearer for patients to navigate, to reduce repetition and patient frustration. Onward referrals were often a pinch point in the system, with patients at risk of increased waiting times or being overlooked. Guidance and support for streaming |



For peer review only - http://bmjopen.bmj.com/site/about/guidelines.xhtml

| | |
|---|---|
| | nurses experiencing complaints processes, litigation or professional registration issues should be provided. |
| **Staffing** | Less reliance on locum GPs and ensuring GPED shifts are covered consistently, and communicated effectively, promotes consistency. Recruitment of highly experienced and clinically knowledgeable GPs who are willing to adapt their practice to take on a broader range of work<br><br>Consider retention strategies to support current streaming nurses and to futureproof streaming by training and retaining adequate numbers of suitably experienced nurses. Streamers should be supported by their professional colleagues. Implement strategies to mitigate against burnout, prevent overload from additional responsibilities and positive promotion of streaming roles to make them attractive to nurses. |
| **Leadership** | Involve staff of all grades and from all key professional groups in the development and implementation of service planning, organisation and protocol development to counteract feelings of top-down change and encourage buy-in and support. |
| **Physical environment** | Consider the impact of the physical environment, e.g. privacy at the streaming desk, safety of both staff and patients in isolated or exposed streaming areas, and for GPs located away from the ED and in off-site Hubs. Inadequate space can lead to overcrowding. Patients who have to queue more than can become confused and frustrated. Consider where GPs are placed to avoid feeling isolated and separated from the ED. |
| **Integrated IT systems** | Effective, easy to use and joined up information technology systems between ED, GPED and General Practice are essential for a safe working environment. |
| **Structural support** | Support for streamers should include specific training, regular supervision, audit and feedback. GPED models and streaming services should be planned and organised with involvement and buy-in from key stakeholders including streaming nurses and GPs. |

*GPED: General Practitioners working in or alongside the Emergency Department; ED: Emergency Department; GP: General Practitioner.*



For peer review only - http://bmjopen.bmj.com/site/about/guidelines.xhtml

GPED is a new policy initiative, which has been evaluated by two large NIHR commissioned research studies (HS&DR Projects 15/145/04 and 15/145/06).[26,35,39,54] Further research evaluating its impact is therefore not recommended until the policy has been given time to embed into routine practice. Instead, priority should be given to evaluating existing performance measures and developing new, rapid methods to inform the development, implementation and evaluation of similar health policy initiatives (Box 2).

**Box 2: Implications for future research**

1. The utility and completeness of national routine data sets limit the ability to evaluate the impact of complex health initiatives across a range of outcomes. Patients and clinicians should be consulted to ensure that measures of 'success' include outcomes that are important to all stakeholder groups and how these can be captured.
2. The relationship and interface between general practice and secondary care is crucial to the future delivery of urgent and emergency care. Research to explore this relationship and different approaches to risk will inform future models of service development and delivery in the context of rising healthcare demand.
3. We identified particular ambiguity and uncertainty in relation to streaming in the ED. Further research to clarify the optimal approach to streaming in terms of patient outcome, safety and experience, and the wider implications of streaming on staff experience, is warranted.

## Strengths and limitations

We adopted a mixed-methods approach which consisted of 'big qualitative' data collection (413 interviews and 142 individual observations of clinical encounters) and quantitative analysis of national data sets to explore the impact of GPED. This approach, and the decision to interpret our study findings using NPT, provided us with an in-depth understanding of the impact of GPED. This highlighted the complex interplay of political, workforce and social factors that affect successful adoption of a health policy into routine practice.

Our data apply to England only, and so may not be generalizable to other countries and healthcare settings. In our quantitative analysis, it was not possible to identify from available data which staff members assessed and treated individual patients, so we could not separate patients treated by GPs from those treated by other ED staff to directly compare GP services



For peer review only - http://bmjopen.bmj.com/site/about/guidelines.xhtml

to traditional models of care. We relied primarily on measures of general ED performance, such as attendances, patient flow and waiting times. We were also limited in our ability to collect data from the general practice and urgent care systems surrounding our case study sites, which significantly limited our ability to evaluate quantitatively the effect of GPED on the wider healthcare system. Our qualitative case study sites were selected purposively to be as representative as possible. However, participation by sites, and from staff and patients during data collection, was voluntary and so is unlikely to be exhaustive.

## Conclusion

Implementation of General Practitioners working in or alongside the ED was highly subject to local context and micro-level influences. However, we found no consistent evidence of improvements in patient outcome or experience. This is summed up by our public contributors, who following presentation of the final study findings concluded:

*"GPED is not effective and should only be used where specific circumstances indicate that it may play a positive role."*

**Contributor and guarantor information**

JB, JA, HBa, KB, SC, TD, AG, NG, SP, CS, SV had the initial research idea and obtained funding for this study. Qualitative data collection and analysis were undertaken by HA, JA, HL, AS. Quantitative data collection and analysis were undertaken by KB, TD, JG, NG, HBr, SC. Mixed methods analysis was undertaken by JA and AS. AS and JA drafted the paper and are joint first authors for the manuscript. JB and CS assisted in drafting the manuscript. AS, JA, CS, HBr, HA, HBa, KB, SC, TD, JG, AG, NG, HL, SP, SV, JB critically reviewed, revised and approved the final manuscript. JB is the study guarantor. The guarantor (JB) accepts full responsibility for the work and the conduct of the study, had access to the data, and controlled the decision to publish. The corresponding author attests that all listed authors meet the authorship criteria and that no others meeting the criteria have been omitted.

**Copyright/license for publication**

"The Corresponding Author has the right to grant on behalf of all authors and does grant on behalf of all authors, a worldwide licence to the Publishers and its licensees in perpetuity, in all forms, formats and media (whether known now or created in the future), to i) publish, reproduce, distribute, display and store the Contribution, ii) translate the Contribution into other



For peer review only - http://bmjopen.bmj.com/site/about/guidelines.xhtml

languages, create adaptations, reprints, include within collections and create summaries, extracts and/or, abstracts of the Contribution, iii) create any other derivative work(s) based on the Contribution, iv) to exploit all subsidiary rights in the Contribution, v) the inclusion of electronic links from the Contribution to third party material where-ever it may be located; and, vi) licence any third party to do any or all of the above.

**Patient consent for publication**

Not applicable

**Data availability statement**

The deidentified patient-level data used for the quantitative component of this study, including information on mortality, were released by the data holders (NHS Digital, Office for National Statistics) under specific data sharing agreements and only for the purpose of this study. The data sharing agreements do not permit further sharing or publication of the data. Interested parties may seek to obtain data directly from the relevant data holders. Hospital Episode Statistics (HES) data are copyright 2018-2019, reused with the permission of NHS Digital through Data Sharing Agreement NIC-84254-J2G1Q. The data about the hours a general practitioner services was operating in emergency departments was collected by the authors specifically for this project. The authors are not able to place the original data into the public domain. The qualitative data we have acquired will not be available as our ethical approval does not permit the sharing of the entire data set.

**Ethical approval**

Ethical approval was obtained from East Midlands – Leicester South Research Ethics Committee (ref: 17/EM/0312); University of Newcastle Ethics Committee (Ref: 14348/2016) and the Health Research Authority (IRAS: 230848 and 218038). All participants provided informed consent before taking part in the qualitative study.

**Transparency declaration**

The lead authors (AS, JA) and manuscript guarantor (JB) affirm that the manuscript is an honest, accurate and transparent account of the study being reported; that no important aspects of the study have been omitted; and that any discrepancies from the study as originally planned (and, if relevant, registered) have been explained.

**Funder**

For peer review only

Department of Health. National Institute for Health Research. Health Services and Delivery Research Programme. Grant number 15/145/06.

**Competing interests**

All authors have completed the ICMJE uniform disclosure form and declare: support from the National Institute for Health Research (NIHR) Health Services and Delivery Programme for the submitted work*; no financial relationships with any organisations that might have an interest in the submitted work in the previous three years; no other relationships or activities that could appear to have influenced the submitted work."*

Acknowledgements

The authors would like to thank the participants for their involvement in the qualitative study, and the patient and public contributors.

## References:

1. NHS Digital. Hospital Accident and Emergency Activity 2017-2018 Online: NHS Digital; 2018 [Available from: https://digital.nhs.uk/data-and-information/publications/statistical/hospital-accident--emergency-activity/2017-18 accessed 23/11/2021 2021.

2. Berchet C. Emergency Care Services. 2015 doi: doi:https://doi.org/10.1787/5jrts344crns-en

3. Morley C, Unwin M, Peterson GM, et al. Emergency department crowding: a systematic review of causes, consequences and solutions. *PloS one* 2018;13(8):e0203316.

4. Morris T, Mason SM, Moulton C, et al. Calculating the proportion of avoidable attendances at UK emergency departments: analysis of the Royal College of Emergency Medicine's Sentinel Site Survey data. *Emergency Medicine Journal* 2018;35(2):114-19. doi: 10.1136/emermed-2017-206846

5. Vogel JA, Rising KL, Jones J, et al. Reasons Patients Choose the Emergency Department over Primary Care: a Qualitative Metasynthesis. *Journal of General Internal Medicine* 2019;34(11):2610-19. doi: 10.1007/s11606-019-05128-x



For peer review only - http://bmjopen.bmj.com/site/about/guidelines.xhtml

6. Coster JE, Turner JK, Bradbury D, et al. Why Do People Choose Emergency and Urgent Care Services? A Rapid Review Utilizing a Systematic Literature Search and Narrative Synthesis. *Academic Emergency Medicine* 2017;24(9):1137-49. doi: 10.1111/acem.13220

7. O'Cathain A, Connell J, Long J, et al. 'Clinically unnecessary' use of emergency and urgent care: A realist review of patients' decision making. *Health Expectations* 2020;23(1):19-40. doi: 10.1111/hex.12995

8. College of Emergency Medicine. Acute and emergency care: prescribing the remedy. London: College of Emergency Medicine, 2014.

9. Urgent and Emergency Care Review Team, Emergency Care Intensive Support Team. Transforming urgent and emergency care services in England. Safer, faster, better: good practice in delivering urgent and emergency care. A guide for local health and social care communities. London: NHS England, 2015.

10. NHS England. Next Steps on the NHS Five Year Forward View. England, 2017.

11. Boeke AJP, van Randwijck-Jacobze ME, de Lange-Klerk EM, et al. Effectiveness of GPs in accident and emergency departments. *British Journal of General Practice* 2010;60(579):e378-e84. doi: 10.3399/bjgp10X532369

12. Cooper A, Davies F, Edwards M, et al. The impact of general practitioners working in or alongside emergency departments: a rapid realist review. *BMJ Open* 2019;9(4):e024501. doi: 10.1136/bmjopen-2018-024501

13. Ramlakhan S, Mason S, O'Keeffe C, et al. Primary care services located with EDs: a review of effectiveness. *Emergency Medicine Journal* 2016;33(7):495-503. doi: 10.1136/emermed-2015-204900

14. Turner J, Coster J, Chambers D, et al. What evidence is there on the effectiveness of different models of delivering urgent care? A rapid review: NIHR Journals Library, Southampton (UK) 2015.

15. Gonçalves-Bradley D, Khangura JK, Flodgren G, et al. Primary care professionals providing non-urgent care in hospital emergency departments. *Cochrane Database of Systematic Reviews* 2018(2) doi: 10.1002/14651858.CD002097.pub4

16. National Institute for Health and Care Excellence. GPs within or on the same site as emergency departments, 2017.

17. Iacobucci G. All emergency departments must have GP led triage by October: British Medical Journal Publishing Group, 2017.



For peer review only - http://bmjopen.bmj.com/site/about/guidelines.xhtml

18. HM Treasury. Spring Budget. London, 2017.

19. Department of Health and Social Care. Funds for next winter will ease pressure on accident and emergency (A&E) departments in England. London, 2017.

20. May CR, Mair F, Finch T, et al. Development of a theory of implementation and integration: Normalization Process Theory. *Implementation Science* 2009;4(1):1-9.

21. May CR, Johnson M, Finch T. Implementation, context and complexity. *Implementation Science* 2016;11(1):1-12.

22. Pope C, Robert G, Bate P, et al. Lost in translation: a multi-level case study of the metamorphosis of meanings and action in public sector organizational innovation. *Public Administration* 2006;84(1):59-79.

23. Travaglia JF, Nugus P, Greenfield D, et al. Contested innovation: the diffusion of interprofessionalism across a health system. *International Journal for Quality in Health Care* 2011;23(6):629-36.

24. Caldwell SE, Mays N. Studying policy implementation using a macro, meso and micro frame analysis: the case of the Collaboration for Leadership in Applied Health Research & Care (CLAHRC) programme nationally and in North West London. *Health research policy and systems* 2012;10(1):1-9.

25. Sausman C, Oborn E, Barrett M. Policy translation through localisation: implementing national policy in the UK. *Policy & Politics* 2016;44(4):563-89.

26. Morton K, Voss S, Adamson J, et al. General practitioners and emergency departments (GPED)—efficient models of care: a mixed-methods study protocol. *BMJ open* 2018;8(10):e024012.

27. May, C.R., Albers, B., Bracher, M. *et al.* Translational framework for implementation evaluation and research: a normalisation process theory coding manual for qualitative research and instrument development. *Implementation Sci* **17,** 19 (2022). https://doi.org/10.1186/s13012-022-01191-x

28. McEvoy R, Ballini L, Maltoni S, O'Donnell CA, Mair FS, MacFarlane A. A qualitative systematic review of studies using the normalization process theory to research implementation processes. Implement Sci. 2014;9:2.

29. May C, Cummings A, Girling M, Bracher M, Mair FS, et al. Using normalization process theory in feasibility studies and process evaluations of complex healthcare interventions: a systematic review. Implement Sci. 2018;13(1):80.



For peer review only - http://bmjopen.bmj.com/site/about/guidelines.xhtml

30. Sheard L, Marsh C. How to analyse longitudinal data from multiple sources in qualitative health research: the pen portrait analytic technique. *BMC medical research methodology* 2019;19(1):1-10.

31. Braun V, Clarke V. Reflecting on reflexive thematic analysis. *Qualitative research in sport, exercise and health* 2019;11(4):589-97.

32. Scantlebury A, Brant H, Anderson H, et al. Potential impacts of general practitioners working in or alongside emergency departments in England: initial qualitative findings from a national mixed-methods evaluation. *BMJ open* 2021;11(5):e045453.

33. NHS Digital. Hospital Accident & Emergency Acitivity 2020-21, 2021.

34. Gaughan J, Liu D, Gutacker N, et al. Does the presence of general practitioners in emergency departments affect quality and safety in English NHS hospitals? A retrospective observational study. *BMJ Open* 2022;12(2):e055976. doi: 10.1136/bmjopen-2021-055976

35. Benger JR, H; Scantlebury, A; Anderson, H; Baxter, H; Bloor, K; Brandling, J; Cowlishaw, S; Doran, T; Gaughan, J; Gibson, A; Gutacker, N; Leggett, H; Liu, D; Morton, K; Purdy, S; Salisbury, C; Vaittinen, A; Voss, S; Watson, R; Adamson, J;. General Practitioners working in or alongside the emergency department: the GPED mixed-methods study. *Health Services and Delivery Research Program* In press

36. O'Cathain A, Murphy E, Nicholl J. Three techniques for integrating data in mixed methods studies. *Bmj* 2010;341

37. Anderson H, Scantlebury A, Leggett H, et al. Factors influencing streaming to General Practitioners in emergency departments: A qualitative study. *International Journal of Nursing Studies* 2021;120:103980.

38. Brant H, Voss S, Morton K, et al. Current provision of general practitioner services in or alongside emergency departments in England. *Emergency Medicine Journal* 2021;38(10):780-83.

39. Cooper A, Edwards M, Brandling J, et al. Taxonomy of the form and function of primary care services in or alongside emergency departments: concepts paper. *Emergency Medicine Journal* 2019;36(10):625-30.

40. The Kings Fund. What's going on with A&E waiting times? Online, 2020.

41. Kool RB, Homberg DJ, Kamphuis HC. Towards integration of general practitioner posts and accident and emergency departments: a case study of two integrated emergency posts



For peer review only - http://bmjopen.bmj.com/site/about/guidelines.xhtml

in the Netherlands. BMC Health Serv Res 2008;8:225. https://doi.org/10.1186/1472-6963-8-225

42. Dale J, Green J, Reid F, Glucksman E, Higgs R. Primary care in the accident and emergency department: II. Comparison of general practitioners and hospital doctors. BMJ 1995;311:427–30. https://doi.org/10.1136/bmj.311.7002.427

43. Wang M, Wild S, Hilfiker G, Chmiel C, Sidler P, Eichler K, et al. Hospital-integrated general practice: a promising way to manage walk-in patients in emergency departments. J Eval Clin Pract 2014;20:20–6. https://doi.org/10.1111/jep.12074

44. Boeke AJ, van Randwijck-Jacobze ME, de Lange-Klerk EM, Grol SM, Kramer MH, van der Horst HE. Effectiveness of GPs in accident and emergency departments. Br J Gen Pract 2010;60:e378–84. https://doi.org/10.3399/bjgp10X532369

45. Carson D, Clay H, Stern R. Primary Care and Emergency Departments: Report From the Primary. Care Foundation. Mar 2010. London: Primary Care Foundation; 2010.

46. Bickerton J, Davies J, Davies H, Apau D, Procter S. Streaming primary urgent care: a prospective approach. Prim Health Care Res Dev 2012;13:142–52. https://doi.org/10.1017/S146342361100017X

47. Uthman OA, Walker C, Lahiri S, Jenkinson D, Adekanmbi V, Robertson W, Clarke A. General practitioners providing non-urgent care in emergency department: a natural experiment. BMJ Open 2018;8:e019736. https://doi.org/10.1136/bmjopen-2017-019736

48. Ablard S, Kuczawski M, Sampson FC, Mason SM. What does the ideal urgent and emergency care system look like? A qualitative study of service user perspectives. Emerg Med J 2020;37:200–5. https://doi.org/10.1136/emermed-2019-208921

49. Ioannidis JPA. Contradicted and Initially Stronger Effects in Highly Cited Clinical Research. JAMA. 2005;294(2):218–228. doi:10.1001/jama.294.2.218

50. Salisbury C, Hollinghurst S, Montgomery A, et al. The impact of co-located NHS walk-in centres on emergency departments. *Emergency Medicine Journal* 2007;24(4):265-69.

51. Pope C, Turnbull J, Jones J, et al. Has the NHS 111 urgent care telephone service been a success. Case study and secondary data analysis in England. *BMJ open* 2017;7(5):e014815.

52. Blunt I, Edwards N, Merry L. What's Behind the A&E 'Crisis'? London: Nuffield Trust; 2015.



For peer review only - http://bmjopen.bmj.com/site/about/guidelines.xhtml

53. Oliver D. David Oliver: Why force GP streaming on NHS emergency departments? BMJ 2020;368:m992. https://doi.org/10.1136/bmj.m992

54. Edwards A. Evaluating effectiveness, safety, patient experience and system implications of different models of using GPs in or alongside Emergency Departments. *London: National Institute for Health Research (NIHR)* 2017

For peer review only



For peer review only - http://bmjopen.bmj.com/site/about/guidelines.xhtml

# GPED –Coding Framework

| | |
|---|---|
| NATIONAL CONTEXT | National - policy, pressures |
| LOCAL CONTEXT | Local – service landscape and population/specific local needs/considerations |
| TRUST ED & UC CULTURE | RESPOND OR RESIST whether staff are actively redirecting patients away from the ED to resist the flow or providing a service in response and recognition that patients have attended with health concerns. Where staff feel they must see patients and responsibility rests with them to provide health care. UCC/GP in ED vs primary care (differences to traditional primary care role. |
| PEN PORTRAIT DATA | Explanation of current system, patient journey through the ED, Layout, History of GPED, future plans, |
| PATIENTS REASONS FOR ATTENDING ED | Patient and staff explanations of why patients attend ED/Previous use of services e.g. have they seen/contacted service before ED |
| SERVICE LITERACY | Any discussions around appropriate/inappropriate attendances, perceived impact of service literacy and actual patient service literacy on use of GPED/ED |
| IMPLEMENTATION | Perceived Challenges and Facilitators to Implementation |
| PERCEIVED IMPACT | Perceived impact of GPED on patient safety, workforce and skills mix, staff interactions, performance/targets, views of GPED |
| EXPECTATIONS OF GPED (T1) | 'hypothesis' from stakeholders at all levels regarding their expectations of what would be the outcome of introduction to GPED. From T1 data, only prospective? |
| OTHER/MISCELLANEOUS INSIGHTS | Potential emerging insights which are outside the current framework but may be significant/to be reviewed with the WPC team regular meetings. |

GP ED

UWE Bristol University of the West of England

**Setting: Established Case Sites**

**Participants: Staff in ED/GPED/KI**

What is your current role in the GPED?

What model of working with GPs/primary care operates in your ED currently?

Were you involved (and in what way) in the design or initial implementation of GPED?

***- only if indicate were involved, ask planning/implementation questions***

**Planning/implementation stage:**

What can you tell us about the initial process of design and implementation of this service

- Key staff involved
- Structural/organisational changes
- Decision making/service design
- Consultation with staff/patients/external bodies

What was expected to be achieved by the change?

What were the key barriers/facilitators?

What were the key issues for staff before the introduction?

What was the attitude/approach to change from management?

**Impact:**

How do you think the GPED model is working?

- Process of selecting patients to be seen by the GP/streaming/getting the 'right' patients
- Key advantages/disadvantages
- Any safety issues

How has it impacted on overall workings of the ED?

- Has there been any impact on performance (e.g. 4 hours, hospital admission rate)
- Resources

Do you think any improvements could be made to the GPED model (aware of different service configurations in other places)?


For peer review only - http://bmjopen.bmj.com/site/about/guidelines.xhtml

What feedback have you had from patients about the GPED model (are they satisfied etc)?

Do you think the availability of this GPED model is likely to change the way the public decide how, where and when to seek care?

***For emergency care staff:***

How has GPED impacted on your own everyday working?

- Clinically (type of patients/presenting conditions)
- Working relationships with other staff (e.g. the staff who select patients to be seen by GP, the GP staff)
- Service provided to patients
- Administratively/organizationally
- Any surprises

***For general practice staff in GPED:***

How is care organised within GP component of GPED?

How does practice within GPED compare to other services (GP practice, walk-in centres):

- Clinically (types of patients/presenting conditions)
- Patient 'outcomes' (e.g. referrals, requests for testing, transfer back to ED)
- Interaction with other professional groups within GP component/ED staff
- Workload
- Any surprises

Discussion around who is employer, professional indemnity, clinical supervision/support around clinical decision making in role as GP in ED

Do you feel you act differently as a practitioner following time in ED (probe – both back in primary care and over time within ED)

Satisfaction with role of GP in ED

- Met with expectations
- Plan to continue in role
- Career plans

How do you think patients have responded to the service?


For peer review only - http://bmjopen.bmj.com/site/about/guidelines.xhtml
For peer review only

GPED

- Why they came to AE rather than GP practice
- Satisfaction with GPED

Any other comments to add about GPED

For peer review only


For peer review only - http://bmjopen.bmj.com/site/about/guidelines.xhtml

GPED

UWE Bristol University of the West of England

**Setting: Existing Case Sites**

**Participants: Patients**

What brought you to the ED on this occasion?

Tell us about what happened after you arrived?

- Who did you see first/what happened next
- Description of being selected to be seen by the GP

Did you know it was possible to be sent to a GP after coming to ED?

- Was this communicated to you
- Did you understand the process/reason you were selected for the GP
- How did you feel about being seen by a GP
- Have you any previous experience of this service (give example)

Explore reason behind attendance at ED for this consultation – why did they use ED over other potential services (walk-in centres, GP surgery)

- Knowledge of different ways to access health services and what they consider the 'appropriate' ways to use them

Would their experiences on this visit change their consultation choice in the future?

Explore awareness of increased demand on EDs/government funding made available to increase GPs in EDs

- Do they think GPs in ED good idea in principle
- What impact do they think it might have on reducing pressure on EDs
- Do they think it will change what patients do

How does practice within GPED compare to other GP services?

How satisfied are they with the visit?

- How long did you have to wait
- How satisfied are you with the outcome
- Can you think of any ways you could improve the service?
- Opportunity to provide feedback

Any other comments to add about GPED.



For peer review only - http://bmjopen.bmj.com/site/about/guidelines.xhtml

## Standards for Reporting Implementation Studies: the StaRI checklist for completion

The StaRI standard should be referenced as: Pinnock H, Barwick M, Carpenter C, Eldridge S, Grandes G, Griffiths CJ, Rycroft-Malone J, Meissner P, Murray E, Patel A, Sheikh A, Taylor SJC for the StaRI Group. Standards for Reporting Implementation Studies (StaRI) statement. *BMJ* 2017;356:i6795

The detailed Explanation and Elaboration document, which provides the rationale and exemplar text for all these items is: Pinnock H, Barwick M, Carpenter C, Eldridge S, Grandes G, Griffiths C, Rycroft-Malone J, Meissner P, Murray E, Patel A, Sheikh A, Taylor S, for the StaRI group. Standards for Reporting Implementation Studies (StaRI). Explanation and Elaboration document. *BMJ Open* 2017 2017;7:e013318

Notes: A key concept of the StaRI standards is the dual strands of describing, on the one hand, the implementation strategy and, on the other, the clinical, healthcare, or public health intervention that is being implemented. These strands are represented as two columns in the checklist.

| | |
|---|---|
| The primary focus of implementation science is the implementation strategy (column 1) and the expectation is that this will always be completed. | The evidence about the impact of the intervention on the targeted population should always be considered (column 2) and either health outcomes reported or robust evidence cited to support a known beneficial effect of the intervention on the health of individuals or populations. |

The StaRI standardsrefers to the broad range of study designs employed in implementation science. Authors should refer to other reporting standards for advice on reporting specific methodological features. Conversely, whilst all items are worthy of consideration, not all items will be applicable to, or feasible within every study.

| Checklist item | | Reported on page # | Implementation Strategy | Reported on page # | Intervention |
|---|---|---|---|---|---|
| | | | "Implementation strategy" refers to how the intervention was implemented | | "Intervention" refers to the healthcare or public health intervention that is being implemented. |
| **Title and abstract** | | | | | |
| Title | **1** | 1 | Identification as an implementation study, and description of the methodology in the title and/or keywords | | |
| Abstract | **2** | 3 | Identification as an implementation study, including a description of the implementation strategy to be tested, the evidence-based intervention being implemented, and defining the key implementation and health outcomes. | | |
| **Introduction** | | | | | |
| Introduction | **3** | 4 | Description of the problem, challenge or deficiency in healthcare or public health that the intervention being implemented aims to address. | | |
| Rationale | **4** | 4, 5 | The scientific background and rationale for the implementation strategy (including any underpinning theory/framework/model, how it is expected to achieve its effects and any pilot work). | | The scientific background and rationale for the intervention being implemented (including evidence about its effectiveness and how it is expected to achieve its effects). |

**Commented [a1]:** This doesn't work



For peer review only - http://bmjopen.bmj.com/site/about/guidelines.xhtml

| | | | | | |
|---|---|---|---|---|---|
| Aims and objectives | **5** | 4 | The aims of the study, differentiating between implementation objectives and any intervention objectives. | | |
| **Methods: description** | | | | | |
| Design | **6** | 4 - 8 | The design and key features of the evaluation, (cross referencing to any appropriate methodology reporting standards) and any changes to study protocol, with reasons | | |
| Context | **7** | 3, 6, 8, 14, 15 | The context in which the intervention was implemented. (Consider social, economic, policy, healthcare, organisational barriers and facilitators that might influence implementation elsewhere). | | |
| Targeted 'sites' | **8** | 3, 6-8 | The characteristics of the targeted 'site(s)' (e.g locations/personnel/resources etc.) for implementation and any eligibility criteria. | | The population targeted by the intervention and any eligibility criteria. |
| Description | **9** | 4, 11-19 | A description of the implementation strategy | | A description of the intervention |
| Sub-groups | **10** | N/A | Any sub-groups recruited for additional research tasks, and/or nested studies are described | | |
| **Methods: evaluation** | | | | | |
| Outcomes | **11** | 3, 6, 7, 8, 9 | Defined pre-specified primary and other outcome(s) of the implementation strategy, and how they were assessed. Document any pre-determined targets | | Defined pre-specified primary and other outcome(s) of the intervention (if assessed), and how they were assessed. Document any pre-determined targets |
| Process evaluation | **12** | N/A | Process evaluation objectives and outcomes related to the mechanism by which the strategy is expected to work | | |
| Economic evaluation | **13** | 8 | Methods for resource use, costs, economic outcomes and analysis for the implementation strategy | | Methods for resource use, costs, economic outcomes and analysis for the intervention |
| Sample size | **14** | 6-8 | Rationale for sample sizes (including sample size calculations, budgetary constraints, practical considerations, data saturation, as appropriate) | | |
| Analysis | **15** | 3, 6, 8 | Methods of analysis (with reasons for that choice) | | |
| Sub-group analyses | **16** | 6-8 | Any a priori sub-group analyses (e.g. between different sites in a multicentre study, different clinical or demographic populations), and sub-groups recruited to specific nested research tasks | | |
| **Results** | | | | | |

| | | | | | |
|---|---|---|---|---|---|
| Characteristics | **17** | 6, 7 | Proportion recruited and characteristics of the recipient population for the implementation strategy | | Proportion recruited and characteristics (if appropriate) of the recipient population for the intervention |
| Outcomes | **18** | 11-13 14-19 | Primary and other outcome(s) of the implementation strategy | | Primary and other outcome(s) of the Intervention (if assessed) |
| Process outcomes | **19** | N/A | Process data related to the implementation strategy mapped to the mechanism by which the strategy is expected to work | | |
| Economic evaluation | **20** | 13, 18-19 | Resource use, costs, economic outcomes and analysis for the implementation strategy | | Resource use, costs, economic outcomes and analysis for the intervention |
| Sub-group analyses | **21** | 11-19 | Representativeness and outcomes of subgroups including those recruited to specific research tasks | | |
| Fidelity/ adaptation | **22** | 11-19 | Fidelity to implementation strategy as planned and adaptation to suit context and preferences | | Fidelity to delivering the core components of intervention (where measured) |
| Contextual changes | **23** | 11-19 | Contextual changes (if any) which may have affected outcomes | | |
| Harms | **24** | N/A | All important harms or unintended effects in each group | | |
| **Discussion** | | | | | |
| Structured discussion | **25** | 19-24 | Summary of findings, strengths and limitations, comparisons with other studies, conclusions and implications | | |
| Implications | **26** | 20-24 | Discussion of policy, practice and/or research implications of the implementation strategy (specifically including scalability) | | Discussion of policy, practice and/or research implications of the intervention (specifically including sustainability) |
| **General** | | | | | |
| Statements | **27** | 5, 24-26 | Include statement(s) on regulatory approvals (including, as appropriate, ethical approval, confidential use of routine data, governance approval), trial/study registration (availability of protocol), funding and conflicts of interest | | |



For peer review only - http://bmjopen.bmj.com/site/about/guidelines.xhtml
